# Multifaceted roles of Meg3 in cellular senescence and atherosclerosis

**DOI:** 10.1016/j.atherosclerosis.2024.117506

**Published:** 2024-03-08

**Authors:** Xiao Cheng, Mohamed Sham Shihabudeen Haider Ali, Vijaya Bhaskar Baki, Matthew Moran, Huabo Su, Xinghui Sun

**Affiliations:** a Department of Biochemistry, University of Nebraska - Lincoln, Beadle Center, 1901 Vine St, Lincoln, NE, 68588, USA; b Vascular Biology Center, Medical College of Georgia, Augusta University, Augusta, GA, 30912, USA; c Department of Pharmacology and Toxicology, Medical College of Georgia, Augusta University, Augusta, GA, 30912, USA; d Nebraska Center for the Prevention of Obesity Diseases Through Dietary Molecules, University of Nebraska - Lincoln, USA

**Keywords:** Long noncoding RNAs, Meg3, Atherosclerosis, Cellular senescence, Vascular endothelium

## Abstract

**Background and aims:**

Long noncoding RNAs are involved in the pathogenesis of atherosclerosis. As long non-coding RNAs maternally expressed gene 3 (Meg3) prevents cellular senescence of hepatic vascular endothelium and obesity-induced insulin resistance, we decided to examine its role in cellular senescence and atherosclerosis.

**Methods and Results:**

By analyzing our data and human and mouse data from the Gene Expression Omnibus database, we found that Meg3 expression was reduced in humans and mice with cardiovascular disease, indicating its potential role in atherosclerosis. In *Ldlr*^−/−^ mice fed a Western diet for 12 weeks, Meg3 silencing by chemically modified antisense oligonucleotides attenuated the formation of atherosclerotic lesions by 34.9% and 20.1% in male and female mice, respectively, revealed by *en-face* Oil Red O staining, which did not correlate with changes in plasma lipid profiles. Real-time quantitative PCR analysis of cellular senescence markers p21 and p16 revealed that Meg3 deficiency aggravates hepatic cellular senescence but not cellular senescence at aortic roots. Human Meg3 transgenic mice were generated to examine the role of Meg3 gain-of-function in the development of atherosclerosis induced by PCSK9 overexpression. Meg3 overexpression promotes atherosclerotic lesion formation by 29.2% in Meg3 knock-in mice independent of its effects on lipid profiles. Meg3 overexpression inhibits hepatic cellular senescence, while it promotes aortic cellular senescence likely by impairing mitochondrial function and delaying cell cycle progression.

**Conclusions:**

Our data demonstrate that Meg3 promotes the formation of atherosclerotic lesions independent of its effects on plasma lipid profiles. In addition, Meg3 regulates cellular senescence in a tissue-specific manner during atherosclerosis. Thus, we demonstrated that Meg3 has multifaceted roles in cellular senescence and atherosclerosis.

## 1. Introduction

Atherosclerosis is a chronic vessel wall disease characterized by low-grade inflammation and lipid accumulation. Many studies greatly improved our understanding of the cellular and molecular mechanisms that contribute to atherosclerosis in recent years [1-3]. Among many new developments, cellular senescence is one of the exciting areas of research [4-6]. Cellular senescence of different cell types within the vessel wall plays an important role in controlling lesion progression, inflammation, and stability [7-9]. Cellular senescence is a very complex process tightly regulated in health and disease. Emerging evidence has demonstrated that long noncoding RNAs regulate cellular senescence through different mechanisms and signaling pathways such as DNA damage response, and subsequently control the initiation and progression of atherosclerosis. For example, the decreased expression of long noncoding RNA small nucleolar host gene-12 accelerated atherosclerotic lesion formation by increasing DNA damage and cellular senescence in the vascular endothelium of *Ldlr* ^−/−^ mice [10]. Similarly, long non-coding RNA NORAD attenuated endothelial cell senescence and atherosclerosis [11]. These studies demonstrated that long noncoding RNAs in the vascular endothelium are important players in the pathogenesis of atherosclerosis by regulating cellular senescence.

Maternally expressed gene 3 (Meg3) is a long noncoding RNA that has been involved in the regulation of different pathophysiological processes. For example, Meg3 silencing alleviates lipopolysaccharide-induced acute lung injury by acting as a molecular sponge of microRNA-7b to modulate inflammasome activation [12]. Meg3 promotes pyroptosis in testicular ischemia-reperfusion injury by targeting microRNA-29a [13]. In addition, Meg3 is specifically expressed in megakaryocytes derived from cord blood hematopoietic stem/progenitor cells but not adult bone marrow [14] and the DLK1-DIO3 cluster noncoding RNAs including Meg3 are upregulated in megakaryopoiesis [15], indicating Meg3 is involved in megakaryopoiesis and thrombopoiesis. Moreover, the plasma levels of Meg3 increase in patients with type 2 diabetes, suggesting it is involved in metabolic disease [16]. Indeed, it suppresses lipid accumulation and inflammation in vitro and the development of fatty liver disease in vivo through histone modification [17]. Recently, it was demonstrated that Meg3 expression is induced in mice and humans with Alzheimer’s disease [18], which contributes to the neuronal cell loss by activating necroptosis. It was reported that Meg3 expression is induced by aging and inhibition of Meg3 improves endothelial cell function both in vitro and in vivo [19]. We found that Meg3 limits DNA damage *in vitro* [20]. Moreover, we found that it prevents cellular senescence of hepatic vascular endothelium in obesity [21]. However, its role in atherosclerosis has not been previously examined. In this study, we examined the role of Meg3 in developing atherosclerosis and regulating cellular senescence using loss-of-function and gain-of-function experiments in different mouse models.

## 2. Materials and methods

### 2.1. Study approval

All mice studies were approved by the University of Nebraska – Lincoln Institutional Animal Care and Use Committee, IACUC protocol #2245 and #2246.

### 2.2. The generation of Meg3 knock-in mice

Ai9 plasmid was purchased from Addgene (Plasmid #22799). The Ai9 plasmid was linearized with FseI to remove the tdTomato gene. Human Meg3 transcript variant 1 (NR_002766.2) was synthesized and cloned into the linearized Ai9 plasmid. The gene of transcript variant 1 was used because we identified it as the most abundantly expressed gene in the vascular endothelium [22]. The plasmid was sent to the Beth Israel Deaconess Transgenic Core Facility to generate loxP-Stop-loxP-Meg3 Rosa26 knock-in (LSL-Meg3 KI) mice. The stop cassette upstream of Meg3 can be removed by Cre recombinase to enable the expression of Meg3. Meg3 knock-in mice were bred with Cdh5CreERT2 Cre mice [23,24] for two or three rounds to generate Cdh5CreERT2:LSL-Meg3 KI mice (Meg3 KI). A single injection of tamoxifen (Sigma T5648; intraperitoneal 10 mg/kg) in corn oil was performed in all control mice or Meg3 KI mice unless otherwise indicated to induce Meg3 expression in the vascular endothelium of mice at 6 weeks of age.

### 2.3. Mouse models of atherosclerosis

Two different mouse models were used to induce atherosclerosis in our studies. First, low-density-lipoprotein receptor (*Ldlr*) knockout mice (*Ldlr*^−/−^; JAX stock no. 002207) were used to examine the role of Meg3 deficiency in atherosclerosis. Seven-week-old male and female *Ldlr* ^−/−^ mice were randomly assigned into two groups and were fed a high-cholesterol diet (Research Diets D12108Ci) for 12 weeks. Mice were injected with negative control gapmeRs or Meg3 gapmeRs (*i.v.* 5 mg/kg) at week 0 and week 2, then weekly injections from week 4 on a high-cholesterol diet. *In vivo*-ready negative control A gapmeR (5′A*A*C*A*C*G*T*C*T*A*T*A*C*G*C′3; * indicates phosphorothioate backbone modification) and the Meg3 gapmeR (5′T*C*A*T*C*A*G*T*C*A*G*T*A*G*G*T′3) were purchased from Qiagen at large scale for mouse studies. The GeneGlobe ID of the Meg3 gapmeR is LG00236638 and the negative control A gapmeR is LG00199023-FZA. The Meg3 gapmeRs have been used in previous studies [19,21] from our and other groups to effectively silence Meg3 expression in mice.

Second, atherosclerosis was induced in Meg3 KI mice by a single injection of the AAV8 virus expressing a gain-of-function mutant of proprotein convertase subtilisin/kexin type 9 (PCSK9). Plasmid pAAV/D377Y-mPCSK9 was purchased from Addgene (Plasmid #58376). AAV8 virus expressing gain-of-function PCSK9 was produced by the Penn Vector Core at the University of Pennsylvania. Mice (7 weeks old) received a single dose of AAV8-PCSK9 at 1 × 10^11^ genome copies/mouse. A week later, mice were fed a Western diet (Teklad diet TD.88137) with 18.9 g/L glucose and 23.1 g/L fructose added to the drinking water for 16 weeks.

### 2.4. Atherosclerotic lesion quantification

Aortas were dissected from the mice and stained with Oil Red O (Electron Microscopy Sciences # 26079-15). Lesion areas were measured by *en-face* analysis, as previously described [11].

### 2.5. Aortic intima isolation

The thoracic aortas were dissected from the mice and carefully flushed with 1 x dPBS. Intima was peeled using TRIzol reagent (Invitrogen # 15596026), flushing through the aortas, followed by a 10-s pause, then collected in an Eppendorf tube with ~300 μl in total.

### 2.6. Lipid and lipoprotein analysis

Blood was collected through cardiac puncture. Triglycerides and total cholesterol levels in plasma were measured using kits from Thermo Scientific # TR22421 and # TR13421, and LDL cholesterol was measured using the kit from Crystal Chem #79980.

### 2.7. Reverse transcription and quantitative PCR analysis

RNA was isolated using TRIzol Reagent (Invitrogen) according to the manufacturer’s instructions. 1 μg of RNA was converted to cDNAs using the High-Capacity cDNA Reverse Transcription Kit (Applied Biosystems # 4368814). qPCR was conducted in the CFX Connect Real-Time System (BioRad) using 2x SYBR Green qPCR Master Mix (Selleck Chemicals LLC #B21203). Data were normalized by the Delta Delta Ct method. While mouse β-actin gene *(Actb), Gapdh*, and *Hprt* were used to normalize the expression of *SR-B1, Actb* was used to normalize all other qPCR data. Primers of the different genes are listed in the Supplementary Table 1.

### 2.8. Public dataset and bioinformatics analysis

Publicly available datasets GSE221911 [25] and GSE205931 [26] were downloaded from the Gene Expression Omnibus. Dataset GSE221911 is an RNA-seq profiling of blood from patients with coronary artery disease (GSE221911). The counts were converted into Counts Per Million for normalization. Data from ninety-five patients with dyslipidemia were analyzed and compared. Dataset GSE205931 is a single-cell RNA-seq analysis of mouse thoracic aorta. Mice were fed on a chow diet or a high-fat diet (Teklad TD.88137) for one month.

Seurat (4.3.0) was used to analyze single-cell RNA-seq data. Data from a chow diet sample and a high-fat diet sample were merged into one Seurat project. After quality control filtering [411 (5%) < nCount_RNA <6088 (95%); 300 < nFeature_RNA <2000; and <10% mitochondrial reads], data were normalized using the normalizedData function and scaled using the ScaleData function. After the Principal Component Analysis, the batch effects were corrected using Harmony. The Harmony-corrected data was analyzed for graph-based clustering using the top 20 principal components at a resolution of 0.15. Five cell clusters were manually annotated based on marker genes of each cell type from published single-cell RNA-seq studies of mouse arteries [27-30]. To identify differentially expressed genes in the EC cluster, the cells of the EC cluster in each sample were randomly split into three replicates, and gene expression counts were then aggregated to generate pseudo-bulk data for each sample. Aggregated gene expression counts were used as inputs in DEseq2 (1.38.3) analysis. A volcano plot was generated in RStudio to show the differentially expressed genes (fold change >1.5 and *p* value < 0.05).

### 2.9. Immunostaining and histological analysis

Livers cryostat sections were prepared as previously described [21]. Aortic roots were embedded in OCT Tissue Tek (Fisher Scientific # 4585) and cut into serial cryostat sections at 5 μm. Primary antibodies at dilutions, 1:100 for p21 (Abcam # ab188224), 1:100 for CD31 (BD Pharmingen # 550274), 1:500 for CD68 (BioLegend # 137001), 1:500 Vimentin (D21H3) XP^®^ Rabbit mAb (CST #5741), and 1:400 for Ki67 (CST # 9129S) were used for staining. Secondary antibodies were Cy3-Goat Anti-Rat IgG (code: 112-165-143) from the Jackson ImmunoResearch and DyLight^™^ 649 Goat Anti-Rabbit IgG Antibody (H + L) from the Vector Laboratories (SKU DI-1659). Images were captured using a Nikon Ti-2 inverted fluorescence microscope or an A1R-Ti2 confocal system. Images were quantified using ImageJ [31]. For Figs. 3B and 5B, we counted p21 and CD31 double-positive cells within four regions of interest in the whole liver vasculature. For the images of aortic root staining, two to three different valves of each section were used for quantification.

### 2.10. Bulk RNA-seq for the aortas from Meg3 knock-in mice

The aortic arch and thoracic aorta were carefully dissected from each mouse and perivascular fat was removed. The samples were cut into small pieces in 1.5 ml tubes containing 100 μl 1 x dPBS on ice. The minced tissues were transferred into pre-chilled homogenization tubes with six metal beads in each tube and 900 μl of TRIzol reagent (Invitrogen) was added to each sample for homogenization. After homogenization, the total RNA was isolated according to the manufacturer’s instructions and sent to Novogene for sequencing and standard data analysis to identify differentially expressed genes.

Gene set enrichment analysis: Gene Ontology enrichment analyses and Hallmark terms enrichment analyses were performed on the lists of genes using the Gene Set Enrichment Analysis (GSEA) software (GSEA v4.2.3 for Windows) [32,33]. The reference gene sets are “h.all.v2023.2.Hs.symbols.gmt” and “c5.go.bp.v2023.2.symbols.gmt” in the Molecular Signatures Database. Package ggplot2 (version 3.3.5) was used to plot gene ontology plots in R (version 4.1.2).

### 2.11. Statistical analysis

GraphPad Prism 9.2 software package and R statistical packages were used for statistical analysis. The Shapiro–Wilk test was used to check normality. For two-group comparisons, we used the two-tailed unpaired Student’s t-test for data with normal distribution, and the F-test for equality of variances was used before using the *t*-test; the nonparametric Mann-Whitney *U* test was used for data without passing a normality test. For repeated measures, we performed statistical analysis with a two-way repeated measures ANOVA comparing two groups. All data in graphs were presented as mean ± SEM, and a *p* < 0.05 was considered statistically significant (*p < 0.05, **p < 0.01, ***p < 0.001).

## 3. Results

### 3.1. Meg3 expression was reduced in humans and mice with cardiovascular disease

**Fig. 1.**
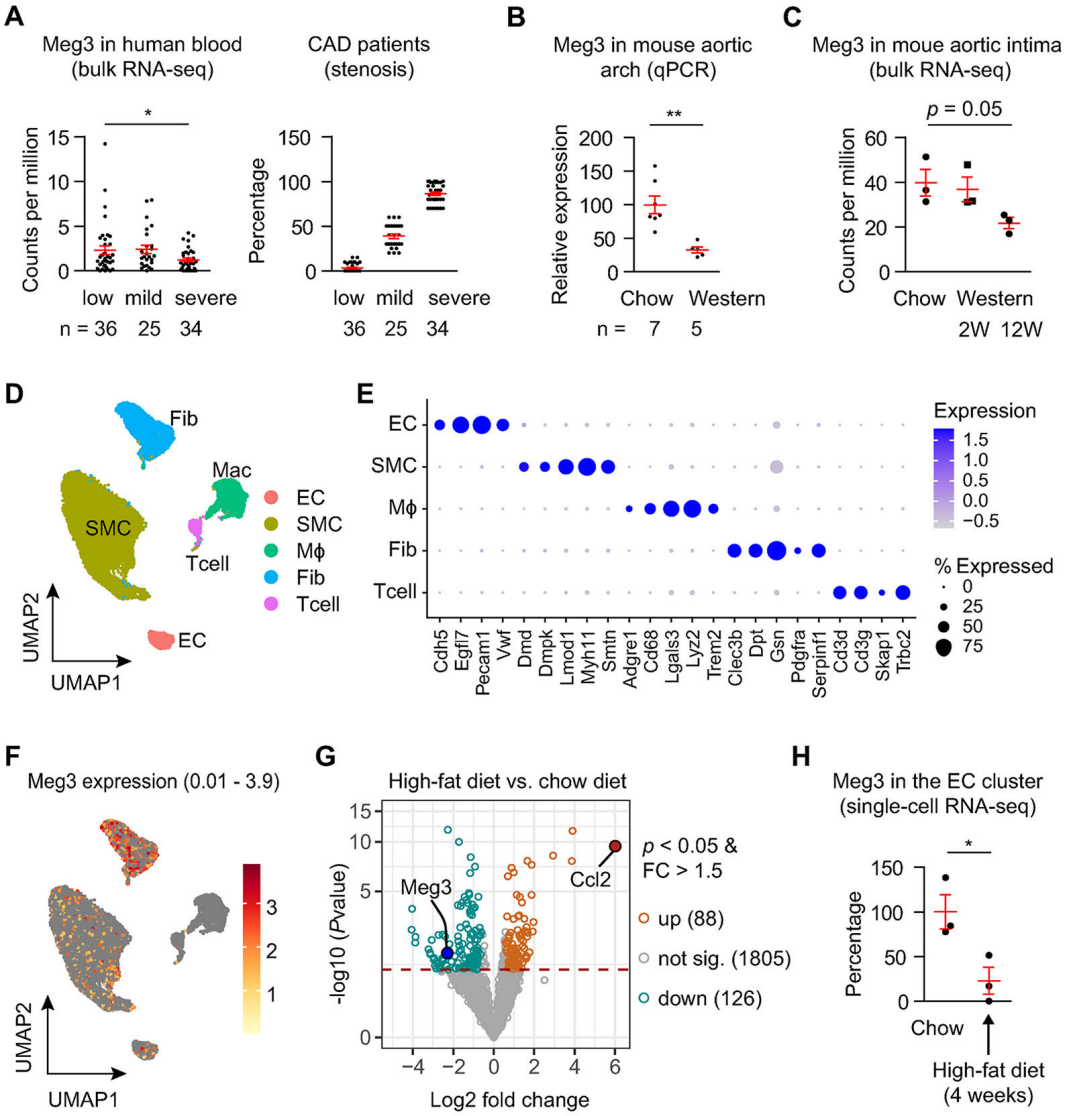
Meg3 expression is reduced in mice and humans with cardiovascular diseases. (A) Meg3 expression in the blood of patients with coronary artery disease by RNA-sequencing. Patients were in three groups with low (n = 36), mild (n = 25), and severe (n = 34) stenosis. (B) Meg3 expression in aortic arches from *Ldlr* ^−/−^ mice fed a chow diet (n = 7) or high-cholesterol diet (n = 5) by qPCR. (C) Meg3 expression in aortic intima from *Ldlr* ^−/−^ mice fed a chow diet or high-cholesterol diet for 2 weeks (2W) or 12 weeks (12W) by RNA-seq (n = 3 per group). (D) A Uniform Manifold Approximation and Projection (UMAP) shows cell clusters in mouse aortas by cell type. Public dataset GSE205931 downloaded from the Gene Expression Omnibus (GEO) database repository was used for analysis in (D–F). It is a single-cell RNA-seq analysis of mouse thoracic aorta. Mice were fed a chow diet or a high-fat diet (Teklad TD.88137) for one month. (E) A dot plot shows the marker genes by cell type. (F) A feature plot shows the expression of Meg3 in different cell clusters. Cells with Meg3 expression less than 0.01 are shown in grey. The maximum Meg3 expression is 3.97 in the endothelial cell cluster, which was selected to be shown in the feature plot. (G) Differentially expressed genes identified by pseudo-bulk analysis of the endothelial cell cluster identified by single-cell RNA-seq in aortas of mice fed a chow diet or high-cholesterol diet. (H) Meg3 expression in the endothelial cell cluster by single-cell RNA-seq in aortas of mice fed a chow diet or high-cholesterol diet (n = 3 per condition). Data represent mean ± SEM; *p < 0.05, **p < 0.01. Two-tailed unpaired Student’s t-test with unequal variances was used for (A) and (B), and two-tailed unpaired Student’s t-test with equal variances was used for (C) and (H).

To examine whether Meg3 is involved in the pathogenesis of atherosclerosis, we first examined Meg3 expression in humans with coronary artery disease (CAD). In CAD patients with severe stenosis, the blood levels of Meg3 were reduced from 2.3 to 1.2 counts per millions by RNA-seq (Fig. 1A). Moreover, the levels of Meg3 expression were reduced by 67.2% in the aortic arches of *Ldlr* ^−/−^ mice fed a Western diet containing 1.25% cholesterol (Fig. 1B). Consistent with the results, data analysis of our previous study [10] revealed that Meg3 expression was reduced from 39.7 to 21.7 counts per millions by RNA-seq in aortic intima of atherosclerotic *Ldlr* ^−/−^ mice (Fig. 1C). Furthermore, we analyzed a public dataset of single-cell RNA-seq [26]. Cell clusters were annotated into endothelial cells (EC), smooth muscle cells (SMC), macrophages (MΦ), fibroblasts (Fib), and T cells (Tcell) (Fig. 1D and E). Meg3 expression is highest in fibroblasts and lowest in macrophages and T cells. At the same time, it had comparable and middle expression in smooth muscle cells and ECs (Fig. 1F). Cells with Meg3 expression lower than 0.01 are in grey (Fig. 1F). We performed a pseudo-bulk analysis of the single-cell RNA-seq data and identified 214 differentially expressed genes in ECs (Fig. 1G). Meg3 is one of the differentially expressed genes, and its expression was reduced by 77.3% in the ECs of the aortas isolated from mice fed on a high-fat diet (Fig. 1H). Together, these data demonstrated that Meg3 expression was reduced in humans and mice with cardiovascular disease, indicating its potential role in the pathogenesis of atherosclerosis.

### 3.2. Meg3 deficiency reduces the formation of atherosclerotic lesions

**Fig. 2.**
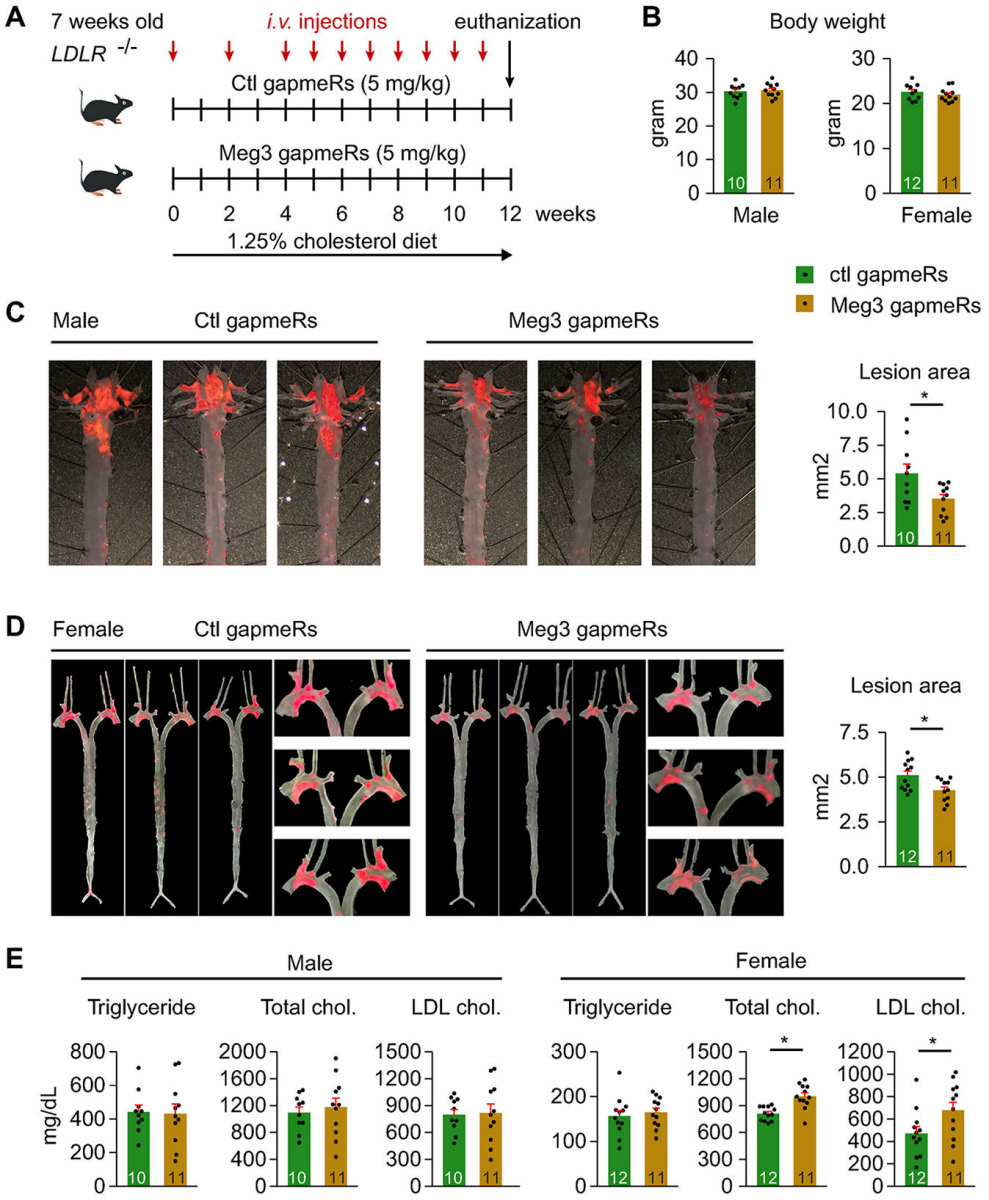
Meg3 deficiency reduces atherosclerotic lesion formation in *Ldlr*^−/−^ mice. (A) Schematic diagram of the injection regimens for negative control and Meg3 gapmeRs. (B) The body weights of mice at the endpoints are shown in grams. (C) Atherosclerotic lesion areas were revealed by Oil Red O staining in the aortas of male mice treated with negative gapmeRs and Meg3 gapmeRs. (D) Atherosclerotic lesion areas were revealed by Oil Red O staining in the aortas of female mice treated with negative gapmeRs and Meg3 gapmeRs. (E) Plasma levels of total cholesterol, LDL cholesterol, and triglycerides in male and female mice. Data represent mean ± SEM; n = 10–12 mice per group; *p < 0.05. Two-tailed unpaired Student’s t-test was used for (C) (unequal variances), (D) (equal variances), and (E) (equal variances).

To assess whether Meg3 is involved in atherosclerosis development, *Ldlr* ^−/−^ mice were fed a Western diet (1.25% cholesterol) for 12 weeks. Mice were injected with chemically modified antisense oligonucleotides (gapmeRs) at 5 mg/kg on Day1 of the Western diet followed by intravenous injection as indicated in Fig. 2A. Meg3 knockdown has no effects on body weights in either females or males throughout the experiments (Fig. 2B; Supplementary Fig. S1A). Quantitative PCR revealed that mice injected with Meg3 gapmeRs displayed markedly reduced Meg3 expression in the aortic intima and media (Supplementary Fig. S1B). In male mice, Oil Red O staining of the aortas revealed a 34.9% reduction in lesion areas in Meg3 gapmeR-treated mice than in control gapmeR-treated mice (Fig. 2C). In female mice, the lesion areas were reduced by 20.1% in Meg3 gapmeR-treated mice (Fig: 2D). Meg3 deficiency did not alter plasma lipid contents including total cholesterol, LDL cholesterol, and triglyceride in male mice (Fig. 2E). However, it induced the plasma levels of total cholesterol and LDL cholesterol by 23.7% and 43.5% (Fig. 2E), respectively, in female mice. In addition, we found that the expression of endothelial scavenger receptor class B type 1 (SR-B1), a receptor mediating the delivery of LDL into arteries [34], was reduced in the aortic intima when Meg3 expression is silenced (Supplementary Fig. S1C). This at least in part explains the decreased lesion formation in Meg3 gapmeR-treated female mice despite the increased plasma levels of total cholesterol and LDL cholesterol. These results demonstrate that Meg3 deficiency attenuates the formation of atherosclerotic lesions in *Ldlr* ^−/−^ mice.

### 3.3. Meg3 deficiency induces cellular senescence in the liver but not the aorta

**Fig. 3.**
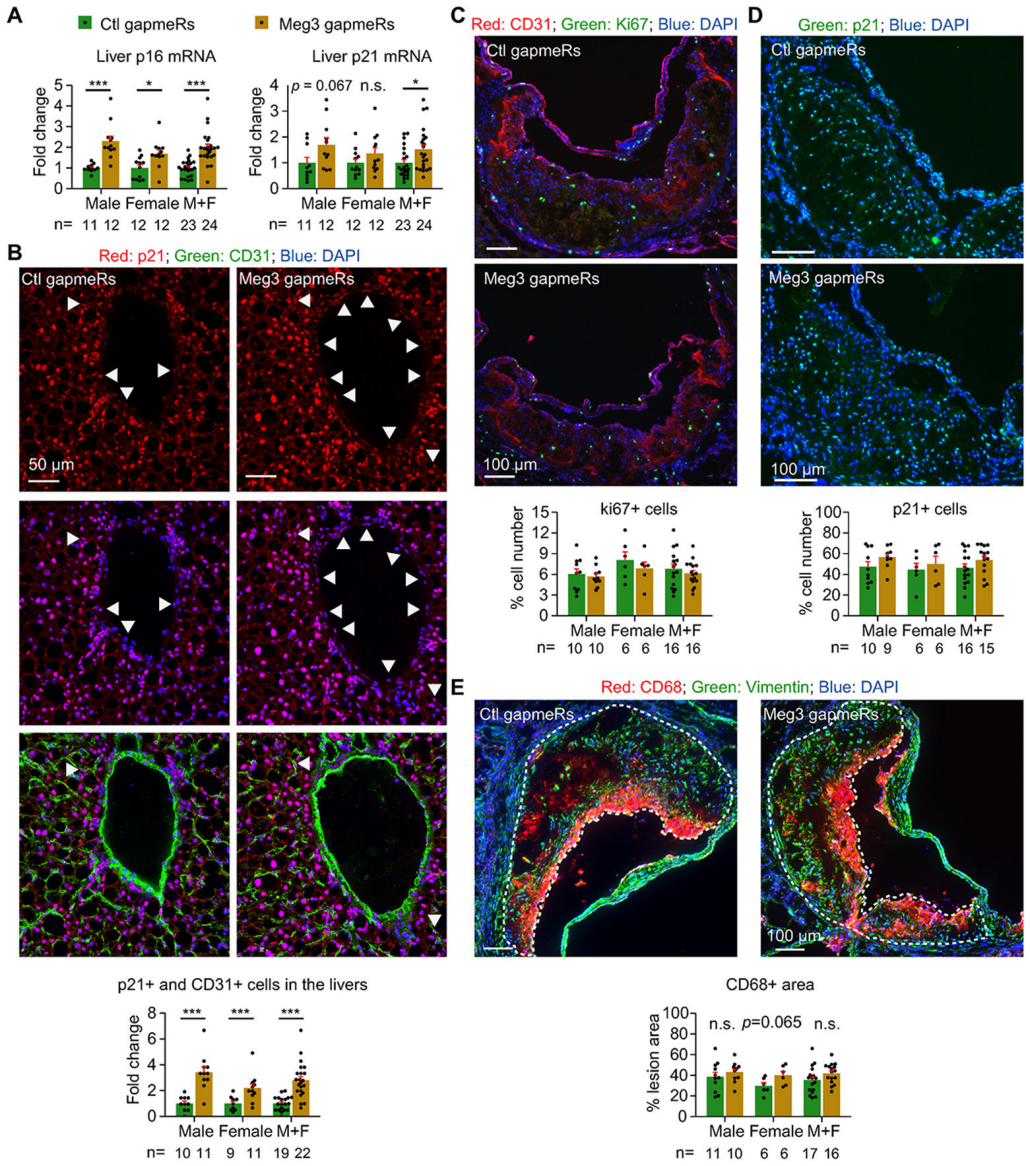
Meg3 deficiency causes cellular senescence in the liver but not the aorta. (A) Quantifications of p21 and p16 at mRNA levels by qPCR in the livers of female and male mice. (B) Immunostaining and quantification of p21 and CD31 double-positive cells on liver sections of mice treated with control or Meg3 gapmeRs. Images from four different views per liver were used for quantification. (C) Histological analysis of cross-sections of the aortic roots from mice stained with CD31, Ki67, and DAPI. Images from 2 to 3 valves per mouse sample were used for quantification. Ki67-positive cells were normalized by DAPI-positive cells. (D) Histological analysis of cross-sections of the aortic roots from mice stained with p21 and DAPI. Images from 2 to 3 valves per mouse sample were used for quantification. p21-positive cells were normalized by DAPI-positive cells. (E) Histological analysis of cross-sections of the aortic roots from mice stained with CD68, vimentin, and DAPI. Images from 2 to 3 valves per mouse sample were used for quantification. The CD68-positive area was normalized by the total lesion area outlined by the white dotted lines. Data represent mean ± SEM; n = 6–12 mice per group separated by sex; *p < 0.05, ***p < 0.0001. Two-tailed unpaired Student’s t-test with unequal variances was used for p16 of males in (A), p21 and CD31 double-positive cells of males in (B) or the combined mice (M + F) in (B); two-tailed unpaired Student’s t-test with equal variances was used for p16 of females in (A), p21 in males or females (A), p21 and CD31 double-positive cells of females in (B), and CD68^+^ area of females in (E); and the Mann-Whitney *U* test was used for p16 and p21 of the combined mice (M + F) in (A).

We previously showed that Meg3 deficiency aggravated cellular senescence of hepatic endothelium in mice fed a 60% high-fat diet [21]. Consistent with the results, the expression of cyclin-dependent kinase inhibitor p16 was increased by 2.3-, 1.7-fold, and 2.0-fold at mRNA levels in the livers of Meg3 gapmeR-treated male, female, and all *Ldlr* ^−/−^ mice, respectively (Fig. 3A). The expression of cyclin-dependent kinase inhibitor p21 was increased by 1.6-, 1.4-fold, and 1.5-fold at mRNA levels in the livers of Meg3 gapmeR-tread male, female, and all mice, respectively, but does not reach significance when data are separated by sex (Fig. 3A). These changes were associated with 92.0% reduction in Meg3 expression in the hepatic endothelium (Supplementary Fig. S1B). Moreover, we observed an increase in the number of p21-positive cells in the hepatic endothelium of mice that received Meg3 gapmeRs (Fig. 3B; arrowheads indicate p21 and p31 double-positive cells). While Meg3 silencing had no effects on the expression of CD68 and TNF-α at mRNA levels, it induced the expression of IL6 mRNAs in the livers, suggesting Meg3 knockdown resulted in hepatic inflammation (Supplementary Fig. S1D). Our data demonstrate that Meg3 deficiency aggravates the cellular senescence of hepatic endothelium in *Ldlr* ^−/−^ mice fed a high-cholesterol diet. Thus, we decided to examine whether Meg3 deficiency contributes to cellular senescence at the aortic roots by staining the nuclear proteins Ki67 and p21 (Fig. 3C and D). Surprisingly, neither the number of Ki67 nor p21 positive cells differs between the two groups of mice at the aortic roots (Fig. 3C and D). Consistent with the results, the levels of p16 and p21 mRNAs were not changed by Meg3 deficiency in the aortic intima and media (Supplementary Fig. S2A). These data suggest that Meg3 deficiency does not aggravate the cellular senescence at the aortic roots. To determine whether Meg3 deficiency regulates macrophage accumulation, we examined macrophage marker CD68 by staining at the aortic roots, where vimentin was stained to visualize the tissue morphology. As shown in Fig. 3E, the CD68 positive areas were not different between the two groups of mice, suggesting that macrophage accumulation is not affected by Meg3 deficiency. In addition, the mRNA levels of CD68, TNF-α, and IL6 were not changed by Meg3 deficiency in the aortic intima and media (Supplementary Fig. S2B). Our data demonstrate that Meg3 deficiency has an organ-specific role in regulating cellular senescence.

### 3.4. Human Meg3 knock-in promotes the development of atherosclerosis in mice

**Fig. 4.**
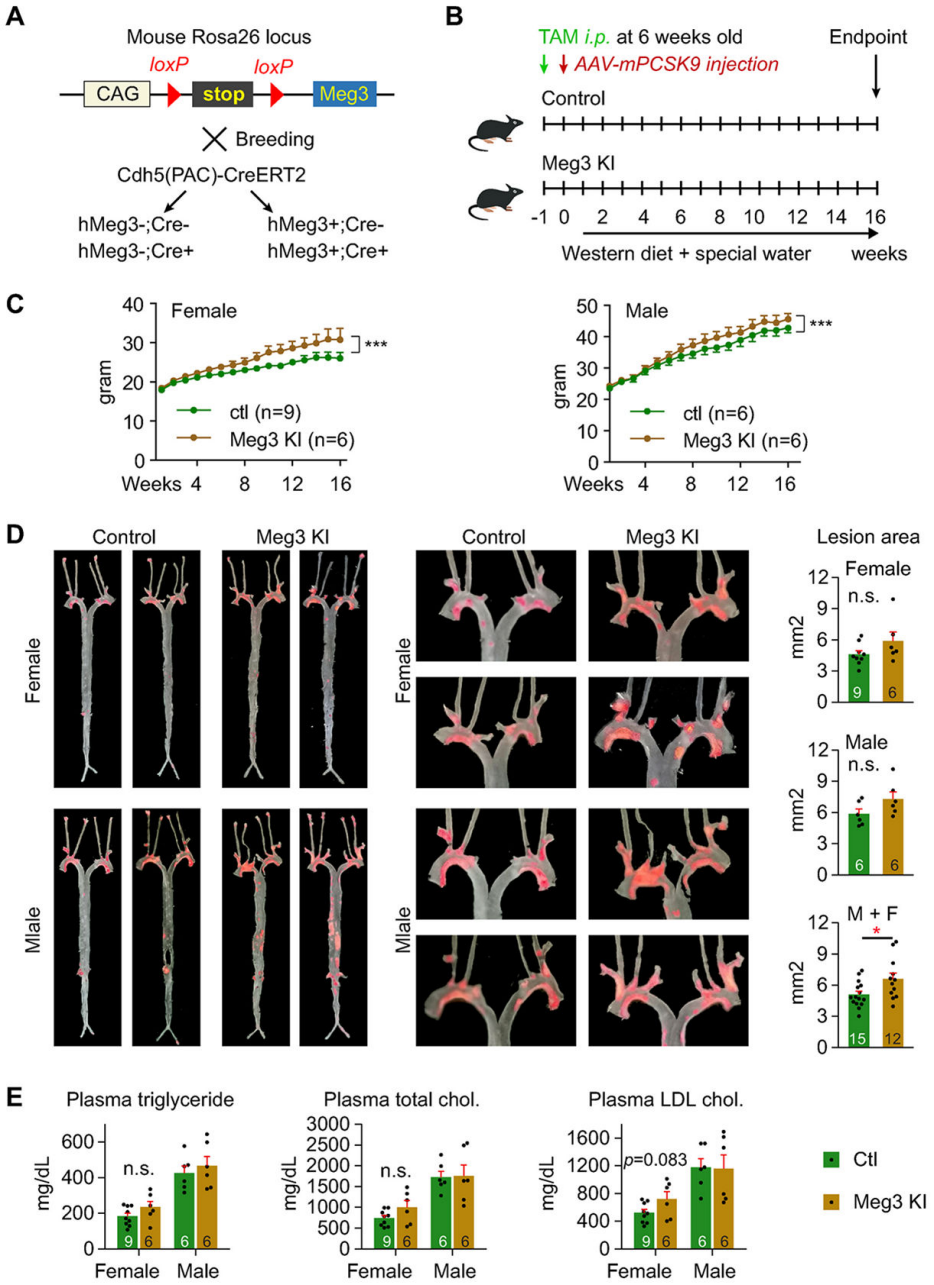
Meg3 knock-in mice develop more atherosclerotic lesions. (A) Schematic diagram of control and human Meg3 knock-in (KI) mice. (B) Schematic diagram of tamoxifen injection, AAV-mPCSK9 injection, western diet, and special water supplemented with fructose and sucrose. (C) Body weights of female and male mice. (D) Atherosclerotic lesion areas were revealed by Oil Red O staining in the aortas of female and male mice. (E) Plasma levels of total cholesterol, LDL cholesterol, and triglycerides in female and male mice. Data represent mean ± SEM; n = 6–9 mice per group separated by sex; *p < 0.05, ***p < 0.001. A two-way repeated measures ANOVA was used for the statistical analysis in (C); two-tailed unpaired Student’s t-test with equal variances was used for comparisons in (D) and (E).

Human Meg3 (hMeg3) transgenic mice were generated to examine the role of gain-of-function of Meg3 in the development of atherosclerosis (Fig. 4A). A floxed stop cassette was inserted between the promoter and hMeg3 gene. Four groups of mice were generated by crossing the hMeg3 transgenic mice with Cdh5(PAC)-CreERT2 mice [23,24], and they are: hMeg3-;Cre−, hMeg3-;Cre+, hMeg3+; Cre−, and hMeg3+; Cre+ (Fig. 4A). These mice were born with the expected Mendelian ratios and did not exhibit any obvious abnormalities. We first examined whether the excision of the stop cassette can turn on the hMeg3 expression. We found a single injection of tamoxifen (*i.p.* 10 mg/kg) induced hMeg3 expression by 59.9- and 7.4-fold in the aortic intima and liver endothelial cells of hMeg3+; Cre + mice, respectively, compared with that in corn-oil treated hMeg3+; Cre + mice (Supplementary Fig. S3A). Thus, we decided to treat mice with tamoxifen at 10 mg/kg to remove the stop cassette and turn on hMeg3 expression in the following studies. The control group of mice were administered tamoxifen at 10 mg/kg as well. The overexpression of PCSK9 was used to induce atherosclerosis (Fig. 4B). We switched to an AAV8-mPCSK9-induced mouse model of atherosclerosis because the breeding pairs of and Meg3 KI mice often did not produce a litter or produce a smaller litter. One week after the tamoxifen injection, both the control mice and Meg3 KI mice received a single injection of AAV-mPCSK9 and fed a high-cholesterol diet with drinking water supplemented with fructose and sucrose for 16 weeks. Both female and male Meg3 KI mice had increased body weights (Fig. 4C). We examined hMeg3 and mouse Meg3 expression in the aortic intima and media (Supplementary Fig. S3B) and found that hMeg3 expression is induced by 77.2- and 7.5-fold in the aortic intima and media of hMeg3+; Cre + mice, respectively, than that in hMeg3+; Cre-mice (Supplementary Fig. S3B). Surprisingly, hMeg3 is expressed in aortic intima and media in the absence of Cre expression (Supplementary Fig. S3B). It is not due to false amplification of genomic DNA, as hMeg3 expression was detected in DNAase-treated RNA samples compared with non-treated samples (Supplementary Fig. S3C), despite its expression being reduced in DNAase-treated RNA samples when it has extremely low expression (e.g. in hMeg3+; Cre-mice). These data suggest that the CAG promoter can drive the expression of hMeg3 at a low level even without the removal of the loxP-flanked stop cassette. Because of that, we decided not to include hMeg3+; Cre-mice in the Meg3 KI group, so comparisons were performed between the control group (hMeg3-;Cre− and hMeg3-;Cre+) and the Meg3 KI group (hMeg3+; Cre+). Meg3 KI mice displayed a 29.2% increase in lesion areas revealed by en-face Oil Red O staining of aortas compared with the control mice when mice were not separated by sex (Fig. 4D). However, lesion areas were not different between control and Meg3 KI mice in either male or female mice (Fig. 4D). Plasma levels of triglycerides, total cholesterol, and LDL cholesterol were not different between control and Meg3 KI groups of either female or male mice (Fig. 4E). These results demonstrated that Meg3 gain-of-function promotes atherosclerotic lesion formation in mice independent of any effects on lipid homeostasis.

### 3.5. Human Meg3 knock-in has mild effects on hepatic cellular senescence and inflammation and aortic cellular senescence

**Fig. 5.**
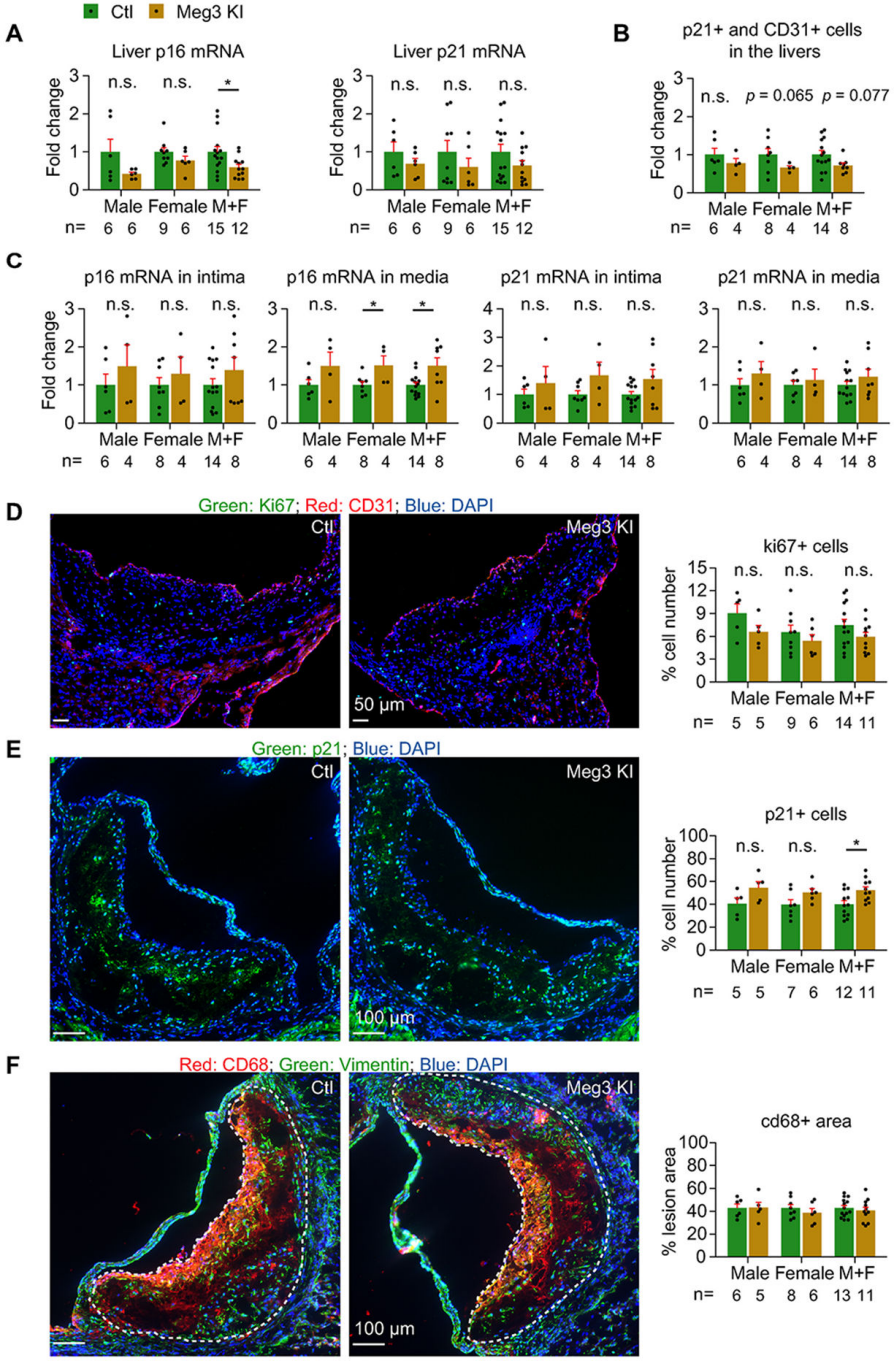
Human Meg3 knock-in has mild effects on hepatic and aortic cellular senescence. (A) Quantification of p16 and p21 mRNAs by qPCR in the livers of control and Meg3 KI mice. (B) Quantification of p21-positive cells in the hepatic endothelium. Images from four different views per liver were used for quantification. (C) Quantification of p16 and p21 mRNAs by qPCR in the aortic intima and media of control and Meg3 KI mice. (D) Immunostaining of Ki67 and CD31 on the aortic root sections of control and Meg3 KI mice. Images from 2 to 3 valves per mouse sample were used for quantification. Ki67-positive cells were normalized by DAPI-positive cells. (E) Immunostaining of p21 on the aortic root sections of control and Meg3 KI mice. Images from 2 to 3 valves per mouse sample were used for quantification. p21-positive cells were normalized by DAPI-positive cells. (F) Immunostaining of CD68 and vimentin on the aortic root sections of control and Meg3 KI mice. Images from 2 to 3 valves per mouse sample were used for quantification. The CD68-positive areas were normalized by the total lesion areas outlined by the white dotted lines. Data represent mean ± SEM; n = 4–9 mice per group separated by sex; *p < 0.05. Two-tailed unpaired Student’s t-test with unequal variances was used for p16 of males or the combined mice (M + F) in (A), p21 and CD31 double-positive cells of females in (B), and p16 mRNA in media of the combined mice (M + F) in (F); two-tailed unpaired Student’s t-test with equal variances was used for p16 of females in (A), p21 of males in (A), p21 and CD31 double-positive cells of males or the combined mice (M + F) in (B), p21-positive cells of all comparisons in (D), and p16 mRNA in media of male or female mice in (F); and the Mann-Whitney *U* test was used for p21 in females or the combined mice (M + F) in (A). The statistical analysis for some non-significant comparisons in (C), (E), and (F) is not described.

Cyclin-dependent kinase inhibitors p21 and p16 are markers of cellular senescence [35-38]. The mRNA levels of p16 were reduced by 40.6% in the livers of Meg3 KI mice. But the difference was not observed in mice separated by sex (Fig. 5A). The expression of p21 tends to be decreased at mRNA levels in the livers of Meg3 KI mice (Fig. 5A). Dual staining of p21 and CD31 revealed that the number of p21 and CD31 double-positive cells tends to be reduced by 34.4% (*p* = 0.065) and 28.6% (*p* = 0.077) in the livers of female and all Meg3 KI mice, respectively (Fig. 5B). To examine the effects of Meg3 on hepatic inflammation, we detected the mRNA levels of CD68, TNFα, and IL6. While Meg3 overexpression tends to decrease IL6 mRNA levels in the liver, it reduces the expression of CD68 and TNF-α at mRNA levels when mice were not separated by sex (Supplementary Fig. S4), suggesting Meg3 overexpression inhibits hepatic inflammation. Next, we examined cellular senescence at aortic roots by qPCR analysis of p16 and p21 and by immunostaining of p21 and Ki67. We found that the expression of p16 mRNA but not p21 was induced 1.5-fold in the aortic media of either female or all Meg3 KI mice (Fig. 5C). The numbers of Ki67-positive cells are not different between all comparisons (Fig. 5D). The number of p21-positive cells is increased by 1.3-fold at the aortic roots between control and Meg3 KI mice when mice are not separated by sex (Fig. 5E). These data suggest that Meg3 overexpression likely promotes aortic cellular senescence. Lastly, we examined the effects of Meg3 overexpression on macrophage accumulation, TNFα expression, and IL6 expression in aortic intima and media. The expression of TNFα mRNAs and macrophage marker CD68 mRNAs was not different between any comparisons (Supplementary Fig. S5). While the expression of IL6 mRNAs tends to be increased in the aortic media of Meg3 KI mice, it was increased by 1.6-fold in the aortic intima of Meg3 KI mice when data are not separated by sex (Supplementary Fig. S5). Moreover, we found that Meg3 overexpression did not alter macrophage content revealed by CD68 staining at the aortic roots of mice (Fig. 5F). These data indicate that Meg3 overexpression promotes the inflammatory response characterized by IL6 mRNA expression in the aorta.

**Fig. 6.**
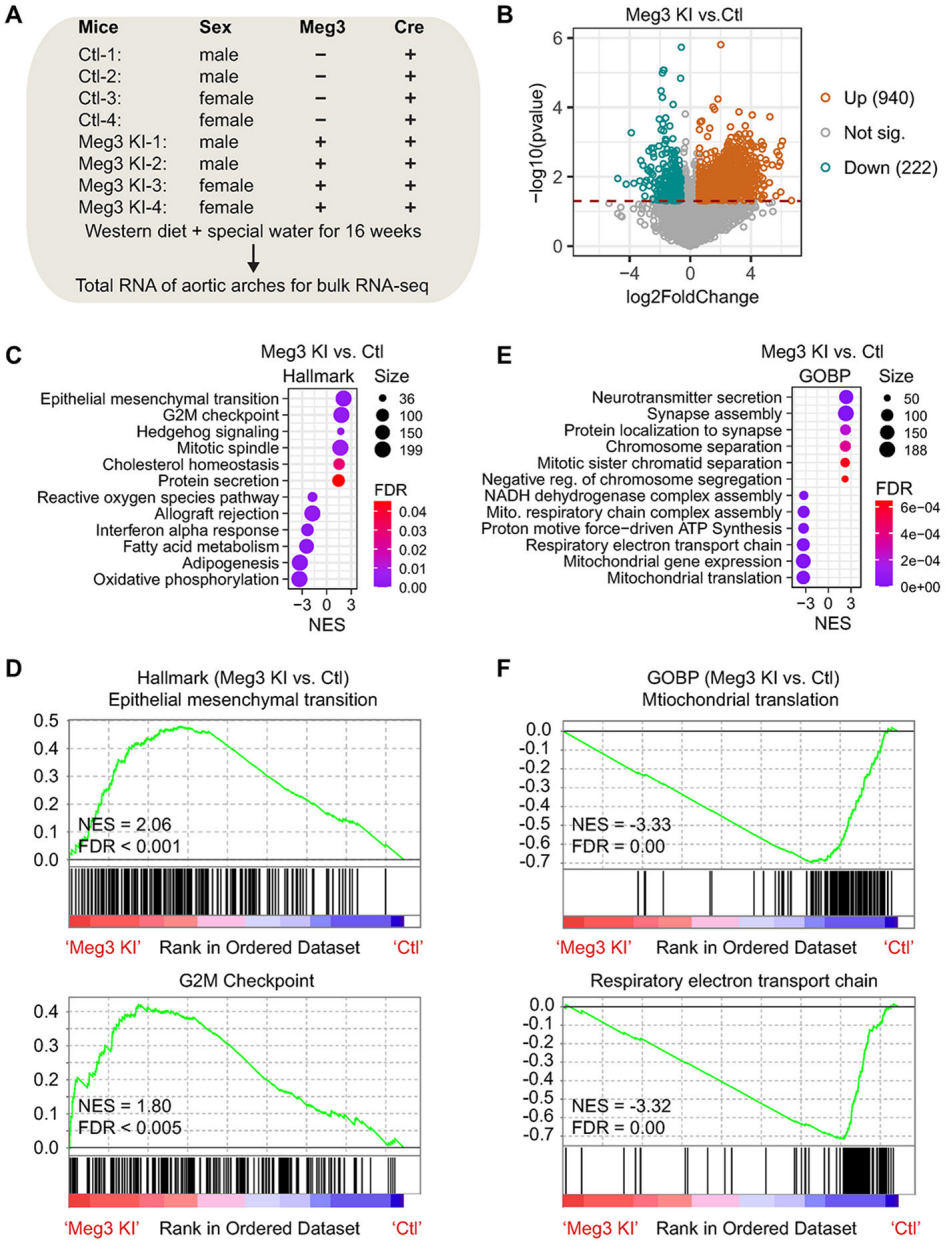
Human Meg3 knock-in alters the transcriptome in mouse aortas. (A) Schematic diagram of mice and RNA samples for bulk RNA-seq. (B) A Volcano plot shows the differentially expressed genes in the aortas of control and Meg3 KI mice. (C) Gene Set Enrichment Analysis revealed top Hallmark terms that are enriched in Meg3 KI or control mice. (D) Enrichment plots of two top Hallmark terms. (E) Gene Set Enrichment Analysis revealed top Gene Ontology Biological Process (GOBP) terms that are enriched in Meg3 KI or control mice. (F) Enrichment plots of two top Gene Ontology Biological Process terms.

To examine the effects of Meg3 overexpression on the transcriptome in the aorta, we did bulk RNA-seq (Fig. 6A). It identified 940 up-regulated genes and 222 down-regulated genes in the aortas of Meg3 KI mice (Fig. 6B; Supplementary Table 2). Gene Set Enrichment Analysis revealed that the top up-regulated Hallmark terms are Epithelial mesenchymal transition, G2M checkpoint, Hedgehog signaling, and Mitotic spindle among others in the aortas of Meg3 KI mice, while the top down-regulated Hallmark terms are oxidative phosphorylation, adipogenesis, and others (Fig 6C and D). The top up-regulated Gene Ontology biological process terms include chromosome separation, mitotic sister chromatid separation, and others; and the top down-regulated terms include mitochondrial translation, mitochondrial gene expression, respiratory, electron transport chain, and others (Fig. 6E and F). Our data indicate Meg3 overexpression impairs mitochondrial function and delays cell cycle progression which warrants future studies.

In summary, our data suggest that Meg3 overexpression inhibits hepatic cellular senescence but promotes aortic cellular senescence and inflammation and the formation of atherosclerotic lesions in Meg3 KI mice likely through the regulation of mitochondrial function and cell cycle progression independent of macrophage accumulation.

## 4. Discussion

Our studies indicate that Meg3 has complex roles in regulating cellular senescence of the vascular endothelium and the development of atherosclerosis in response to nutritional stress. Using gapmeRs to knock down Meg3 expression, we found that Meg3 deficiency resulted in the cellular senescence of hepatic endothelium but had no effects on it in the aortic endothelium. In addition, Meg3 deficiency reduced the formation of atherosclerotic lesions. Consistent with the results, Meg3 knock-in mice displayed an increase in atherosclerotic lesion formation in the aortas, and Meg3 overexpression attenuates the cellular senescence in hepatic endothelium while tends to increase aortic cellular senescence. Our data demonstrate that Meg3 has tissue-specific roles in cellular senescence and Meg3 expression promotes the development of atherosclerosis.

Cell senescence is a stable form of cell cycle arrest characterized by a number of hallmarks, including mitochondrial dysfunction, DNA damage, increased expression of cyclin-dependent kinase inhibitors (e.g., p16 and p21), senescence-associated β-galactosidase activity, and senescence-associated secretory phenotype [39-42]. Senescent cells can be detected by examining a combination of non-exclusive markers [35-37,39,43-45], such as DNA damage, cell proliferation, senescence-associated β-galactosidase activity, increased expression of cyclin-dependent kinase inhibitors, decreased Lamin B1 expression, mitochondrial dysfunction, and others. Among these senescent markers, cyclin-dependent kinase inhibitor p16 is commonly used as an in vivo marker of senescence [35-37]. We have previously demonstrated that Meg3 deficiency induced the DNA damage response and cellular senescence in human umbilical vein endothelial cells and caused obesity-induced insulin resistance by promoting the cellular senescence of hepatic endothelium in obese mice [21]. In the current study, Meg3 deficiency did not cause cellular senescence in the aortas in the mouse model of atherosclerosis, although it still caused the cellular senescence of the hepatic endothelium. The discrepancy could result from at least two reasons. First, a high-cholesterol diet could overwrite Meg3’s effect on the cellular senescence of the aortic endothelium. Second, unknown mechanisms or molecules could be present in aortas that compensate for the deficiency of Meg3 expression. In this study, we mainly assessed cellular senescence by examining the expression of p21 and p16. However, their mRNA levels do not always correlate with their protein levels [46,47] and the expression of typical housekeeping genes for normalization is altered in senescence [48]. We normalized our quantitative PCR data using either Gapdh or β-actin genes and found that the effects of Meg3 on p16 and p21 expression were not altered. Despite we examined p21 protein expression by immunostaining, the assessment of other senescent markers such as Lamin B1 expression, or the phosphorylation of the histone H2AX will strengthen our results.

It remains uncharacterized how Meg3 modulates the formation of atherosclerotic lesions. Hyperlipidemia is a key factor that drives the development of atherosclerosis. It is counterintuitive that decreased lesion formation in the aorta is associated with increased total and LDL plasma cholesterol in Meg3 gapmeR-treated mice. Potential explanations are: 1) cholesterol efflux is increased in the aorta of *Ldlr*^−/−^ mice treated with Meg3 gapmeRs and 2) the delivery of LDL into arteries through the endothelial scavenger receptor class B type 1 (SR-B1) [34] is attenuated in the aorta when Meg3 expression is silenced. It is known there are important sex differences in cholesterol metabolism [49]. Meg3 silencing increased the levels of total cholesterol and LDL cholesterol in female but not male *Ldlr*^−/−^ mice, suggesting Meg3 has a sex-specific role in regulating cholesterol metabolism. Our data suggest that the role of Meg3 in atherosclerosis is independent of lipid homeostasis and macrophage infiltration. Several studies revealed that Meg3 is involved in the regulation of Nlrp3 inflammasome and pyroptosis [12,50,51]. We speculate that Meg3 exacerbates the activation of Nlrp3 inflammasome and subsequent pyroptosis in the aortas of mice fed a high-cholesterol diet. In support of the speculation, Meg3 overexpression in the vascular endothelium likely impairs mitochondrial function and delays cell cycle progression (Fig. 6). Increased oxidative stress due to mitochondrial dysfunction can sensitize gasdermin cleavage, enhance their function, and cause pyroptosis [52,53]. Future studies are required to investigate the possibility.

Our study has several additional limitations. We did not examine Meg3 expression by quantitative PCR in human arteries and the hepatic endothelium of human livers. In addition, there are weaknesses in studying human lncRNAs in mice despite strengths [54]. Human lncRNAs (e.g. human Meg3 in this study) may not fully recapitulate its spatiotemporal pattern expression profile in the vascular endothelium of mice, because of the absence of human-specific regulatory cassettes or cofactors [54].

**Fig. 7.**
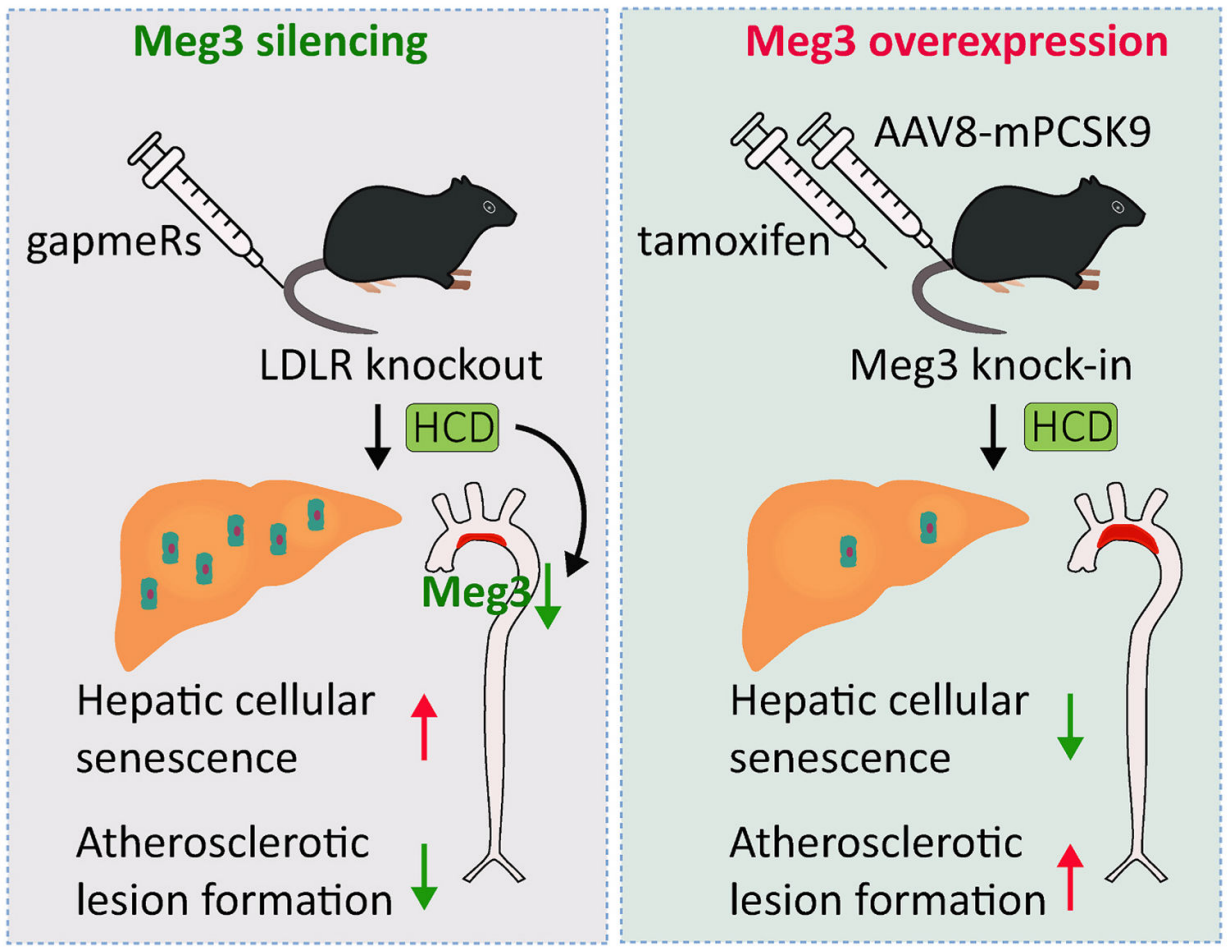
Multifaceted roles of Meg3 in cellular senescence and atherosclerosis. Meg3 silencing induces cellular senescence in the liver and attenuates the formation of atherosclerotic lesions in *Ldlr*^−/−^ mice. Human Meg3 knock-in reduces hepatic cellular senescence and accelerates the formation of atherosclerotic lesions induced by PCSK9 overexpression.

In conclusion, we demonstrated that Meg3 promotes the development of atherosclerosis in response to a high-cholesterol diet (Fig. 7). Moreover, our data suggest that Meg3 has a tissue-specific role in regulating the cellular senescence of vascular endothelium (Fig. 7). Future studies will provide great insight into the mechanisms by which Meg3 regulates atherosclerosis and cellular senescence of the vascular endothelium in a tissue-specific manner.

## Supplementary Material

supplemental materials

## References

[1] Libby P, The changing landscape of atherosclerosis, Nature 592 (2021) 524–533. 33883728 10.1038/s41586-021-03392-8

[2] Farina FM, Weber C, Santovito D, The emerging landscape of non-conventional RNA functions in atherosclerosis, Atherosclerosis (2023). 10.1016/j.atherosclerosis.2023.01.009 36725418

[3] Bjorkegren JLM, Lusis AJ, Atherosclerosis: recent developments, Cell 185 (2022) 1630–1645. 35504280 10.1016/j.cell.2022.04.004 PMC9119695

[4] Suda M, Paul KH, Minamino T, , Senescent cells: a therapeutic target in cardiovascular diseases, Cells (2023) 12. 10.3390/cells12091296 PMC10177324 37174697

[5] Vellasamy DM, Lee SJ, Goh KW, , Targeting immune senescence in atherosclerosis, Int. J. Mol. Sci 23 (2022). 10.3390/ijms232113059 PMC9658319 36361845

[6] Sun X, Feinberg MW, Vascular endothelial senescence: pathobiological insights, emerging long noncoding RNA targets, challenges and therapeutic opportunities, Front. Physiol 12 (2021) 693067. 34220553 10.3389/fphys.2021.693067 PMC8242592

[7] Grootaert MOJ, Finigan A, Figg NL, , SIRT6 protects smooth muscle cells from senescence and reduces atherosclerosis, Circ. Res 128 (2021) 474–491. 33353368 10.1161/CIRCRESAHA.120.318353 PMC7899748

[8] Kotla S, Le NT, Vu HT, , Endothelial senescence-associated secretory phenotype (SASP) is regulated by Makorin-1 ubiquitin E3 ligase, Metab., Clin. Exp 100 (2019) 153962. 31476350 10.1016/j.metabol.2019.153962 PMC7059097

[9] Childs BG, Baker DJ, Wijshake T, , Senescent intimal foam cells are deleterious at all stages of atherosclerosis, Science 354 (2016) 472–477. 27789842 10.1126/science.aaf6659 PMC5112585

[10] Haemmig S, Yang D, Sun X, , Long noncoding RNA SNHG12 integrates a DNA-PK-mediated DNA damage response and vascular senescence, Sci. Transl. Med (2020) 12. 10.1126/scitranslmed.aaw1868 32075942

[11] Bian W, Jing X, Yang Z, , Downregulation of LncRNA NORAD promotes Ox-LDL-induced vascular endothelial cell injury and atherosclerosis, Aging 12 (2020) 6385–6400. 32267831 10.18632/aging.103034 PMC7185106

[12] Liao H, Zhang S, Qiao J, Silencing of long non-coding RNA MEG3 alleviates lipopolysaccharide-induced acute lung injury by acting as a molecular sponge of microRNA-7b to modulate NLRP3, Aging 12 (2020) 20198–20211. 32852284 10.18632/aging.103752 PMC7655187

[13] Ning JZ, He KX, Cheng F, , Long non-coding RNA MEG3 promotes pyroptosis in testicular ischemia-reperfusion injury by targeting MiR-29a to modulate PTEN expression, Front. Cell Dev. Biol 9 (2021) 671613. 34222244 10.3389/fcell.2021.671613 PMC8249820

[14] Liu Y, Zhao J, Wang Y, , Augmented production of platelets from cord blood with euchromatic histone lysine methyltransferase inhibition, Stem. Cells Transl. Med 11 (2022) 946–958. 35880582 10.1093/stcltm/szac048 PMC9492236

[15] Schwarzer A, Emmrich S, Schmidt F, , The non-coding RNA landscape of human hematopoiesis and leukemia, Nat. Commun 8 (2017) 218. 28794406 10.1038/s41467-017-00212-4 PMC5550424

[16] Alrefai AA, Khader HF, Elbasuony HA, , Evaluation of the expression levels of lncRNAs H19 and MEG3 in patients with type 2 diabetes mellitus, Mol. Biol. Rep 50 (2023) 6075–6085. 37294471 10.1007/s11033-023-08569-0

[17] Zou D, Liu L, Zeng Y, , LncRNA MEG3 up-regulates SIRT6 by ubiquitinating EZH2 and alleviates nonalcoholic fatty liver disease, Cell Death Dis. 8 (2022) 103. 10.1038/s41420-022-00889-7 PMC8901640 35256601

[18] Balusu S, Horre K, Thrupp N, , MEG3 activates necroptosis in human neuron xenografts modeling Alzheimer’s disease, Science 381 (2023) 1176–1182. 37708272 10.1126/science.abp9556 PMC7615236

[19] Boon RA, Hofmann P, Michalik KM, , Long noncoding RNA Meg3 controls endothelial cell aging and function: implications for regenerative angiogenesis, J. Am. Coll. Cardiol 68 (2016) 2589–2591. 27931619 10.1016/j.jacc.2016.09.949

[20] Lan Y, Li YJ, Li DJ, , Long noncoding RNA MEG3 prevents vascular endothelial cell senescence by impairing miR-128-dependent Girdin downregulation, Am. J. Physiol. Cell Physiol 316 (2019) C830–C843. 30576236 10.1152/ajpcell.00262.2018

[21] Cheng X, Shihabudeen Haider Ali MS, Moran M, , Long non-coding RNA Meg3 deficiency impairs glucose homeostasis and insulin signaling by inducing cellular senescence of hepatic endothelium in obesity, Redox Biol. 40 (2021) 101863. 33508742 10.1016/j.redox.2021.101863 PMC7844131

[22] Shihabudeen Haider Ali MS, Cheng X, Moran M, , LncRNA Meg3 protects endothelial function by regulating the DNA damage response, Nucleic Acids Res. 47 (2019) 1505–1522. 30476192 10.1093/nar/gky1190 PMC6379667

[23] Sorensen I, Adams RH, Gossler A, DLL1-mediated Notch activation regulates endothelial identity in mouse fetal arteries, Blood 113 (2009) 5680–5688. 19144989 10.1182/blood-2008-08-174508

[24] Wang Y, Nakayama M, Pitulescu ME, , Ephrin-B2 controls VEGF-induced angiogenesis and lymphangiogenesis, Nature 465 (2010) 483–486. 20445537 10.1038/nature09002

[25] McCaffrey TA, Toma I, Yang Z, , RNAseq profiling of blood from patients with coronary artery disease: signature of a T cell imbalance, J. Mol. Cell. Cardiol 4 (2023). 10.1016/j.jmccpl.2023.100033 PMC10256136 37303712

[26] Ord T, Lonnberg T, Nurminen V, , Dissecting the polygenic basis of atherosclerosis via disease-associated cell state signatures, Am. J. Hum. Genet 110 (2023) 722–740. 37060905 10.1016/j.ajhg.2023.03.013 PMC10183377

[27] Cochain C, Vafadarnejad E, Arampatzi P, , Single-cell RNA-seq reveals the transcriptional landscape and heterogeneity of aortic macrophages in murine atherosclerosis, Circ. Res 122 (2018) 1661–1674. 29545365 10.1161/CIRCRESAHA.117.312509

[28] Li F, Yan K, Wu L, , Single-cell RNA-seq reveals cellular heterogeneity of mouse carotid artery under disturbed flow, Cell Death Dis. 7 (2021) 180. 10.1038/s41420-021-00567-0 PMC8290019 34282126

[29] Kalluri AS, Vellarikkal SK, Edelman ER, , Single-cell analysis of the normal mouse aorta reveals functionally distinct endothelial cell populations, Circulation 140 (2019) 147–163. 31146585 10.1161/CIRCULATIONAHA.118.038362 PMC6693656

[30] de Winther MPJ, Back M, Evans P, , Translational opportunities of single-cell biology in atherosclerosis, Eur. Heart J 44 (2023) 1216–1230. 36478058 10.1093/eurheartj/ehac686 PMC10120164

[31] Schneider CA, Rasband WS, Eliceiri KW, NIH Image to ImageJ: 25 years of image analysis, Nat. Methods 9 (2012) 671–675. 22930834 10.1038/nmeth.2089 PMC5554542

[32] Subramanian A, Tamayo P, Mootha VK, , Gene set enrichment analysis: a knowledge-based approach for interpreting genome-wide expression profiles, Proc. Natl. Acad. Sci. U. S. A 102 (2005) 15545–15550. 16199517 10.1073/pnas.0506580102 PMC1239896

[33] Mootha VK, Lindgren CM, Eriksson KF, , PGC-1alpha-responsive genes involved in oxidative phosphorylation are coordinately downregulated in human diabetes, Nat. Genet 34 (2003) 267–273. 12808457 10.1038/ng1180

[34] Huang L, Chambliss KL, Gao X, , SR-B1 drives endothelial cell LDL transcytosis via DOCK4 to promote atherosclerosis, Nature 569 (2019) 565–569. 31019307 10.1038/s41586-019-1140-4 PMC6631346

[35] Krishnamurthy J, Torrice C, Ramsey MR, , Ink4a/Arf expression is a biomarker of aging, J. Clin. Invest 114 (2004) 1299–1307. 15520862 10.1172/JCI22475 PMC524230

[36] Liu Y, Sanoff HK, Cho H, , Expression of p16(INK4a) in peripheral blood T-cells is a biomarker of human aging, Aging Cell 8 (2009) 439–448. 19485966 10.1111/j.1474-9726.2009.00489.x PMC2752333

[37] Sharpless NE, Sherr CJ, Forging a signature of in vivo senescence, Nat. Rev. Cancer 15 (2015) 397–408. 26105537 10.1038/nrc3960

[38] Yosef R, Pilpel N, Papismadov N, , p21 maintains senescent cell viability under persistent DNA damage response by restraining JNK and caspase signaling, EMBO J. 36 (2017) 2280–2295. 28607003 10.15252/embj.201695553 PMC5538795

[39] Hernandez-Segura A, Nehme J, Demaria M, Hallmarks of cellular senescence, Trends Cell Biol. 28 (2018) 436–453. 29477613 10.1016/j.tcb.2018.02.001

[40] Herranz N, Gil J, Mechanisms and functions of cellular senescence, J. Clin. Invest 128 (2018) 1238–1246. 29608137 10.1172/JCI95148 PMC5873888

[41] Baker DJ, Petersen RC, Cellular senescence in brain aging and neurodegenerative diseases: evidence and perspectives, J. Clin. Invest 128 (2018) 1208–1216. 29457783 10.1172/JCI95145 PMC5873891

[42] Sun X, Harris EN, New aspects of hepatic endothelial cells in physiology and nonalcoholic fatty liver disease, Am. J. Physiol. Cell Physiol 318 (2020) C1200–C1213. 32374676 10.1152/ajpcell.00062.2020 PMC7311747

[43] Debacq-Chainiaux F, Erusalimsky JD, Campisi J, , Protocols to detect senescence-associated beta-galactosidase (SA-betagal) activity, a biomarker of senescent cells in culture and in vivo, Nat. Protoc 4 (2009) 1798–1806. 20010931 10.1038/nprot.2009.191

[44] Dimri GP, Lee X, Basile G, , A biomarker that identifies senescent human cells in culture and in aging skin in vivo, Proc. Natl. Acad. Sci. U. S. A 92 (1995) 9363–9367. 7568133 10.1073/pnas.92.20.9363 PMC40985

[45] Wang AS, Dreesen O, Biomarkers of cellular senescence and skin aging, Front. Genet 9 (2018) 247. 30190724 10.3389/fgene.2018.00247 PMC6115505

[46] Casella G, Munk R, Kim KM, , Transcriptome signature of cellular senescence, Nucleic Acids Res. 47 (2019) 7294–7305. 31251810 10.1093/nar/gkz555 PMC6698740

[47] Gonzalez-Gualda E, Baker AG, Fruk L, , A guide to assessing cellular senescence in vitro and in vivo, FEBS J. 288 (2021) 56–80. 32961620 10.1111/febs.15570

[48] Hernandez-Segura A, Rubingh R, Demaria M, Identification of stable senescence-associated reference genes, Aging Cell 18 (2019) e12911. 30710410 10.1111/acel.12911 PMC6413663

[49] Palmisano BT, Zhu L, Eckel RH, , Sex differences in lipid and lipoprotein metabolism, Mol. Metabol 15 (2018) 45–55. 10.1016/j.molmet.2018.05.008 PMC6066747 29858147

[50] Meng J, Ding T, Chen Y, , LncRNA-Meg3 promotes Nlrp3-mediated microglial inflammation by targeting miR-7a-5p, Int. Immunopharm 90 (2021) 107141. 10.1016/j.intimp.2020.107141 33189612

[51] Liang J, Wang Q, Li JQ, , Long non-coding RNA MEG3 promotes cerebral ischemia-reperfusion injury through increasing pyroptosis by targeting miR-485/AIM2 axis, Exp. Neurol 325 (2020) 113139. 31794744 10.1016/j.expneurol.2019.113139

[52] Devant P, Borsic E, Ngwa EM, , Gasdermin D pore-forming activity is redox-sensitive, Cell Rep. 42 (2023) 112008. 36662620 10.1016/j.celrep.2023.112008 PMC9947919

[53] Wang Y, Shi P, Chen Q, , Mitochondrial ROS promote macrophage pyroptosis by inducing GSDMD oxidation, J. Mol. Cell Biol 11 (2019) 1069–1082. 30860577 10.1093/jmcb/mjz020 PMC6934151

[54] Ghanam AR, Bryant WB, Miano JM, Of mice and human-specific long noncoding RNAs, Mamm. Genome 33 (2022) 281–292. 35106622 10.1007/s00335-022-09943-2 PMC8806012

