# Supplementary material for: Multifaceted roles of Meg3 in cellular senescence and atherosclerosis
Source: Atherosclerosis. Author manuscript; Available in PMC 2024 May 13. (PMC11088985; doi:10.1016/j.atherosclerosis.2024.117506)
Supplement: supplemental materials [file NIHMS1982811-supplement-supplemental_materials.pdf]

Supplementary Figure S1

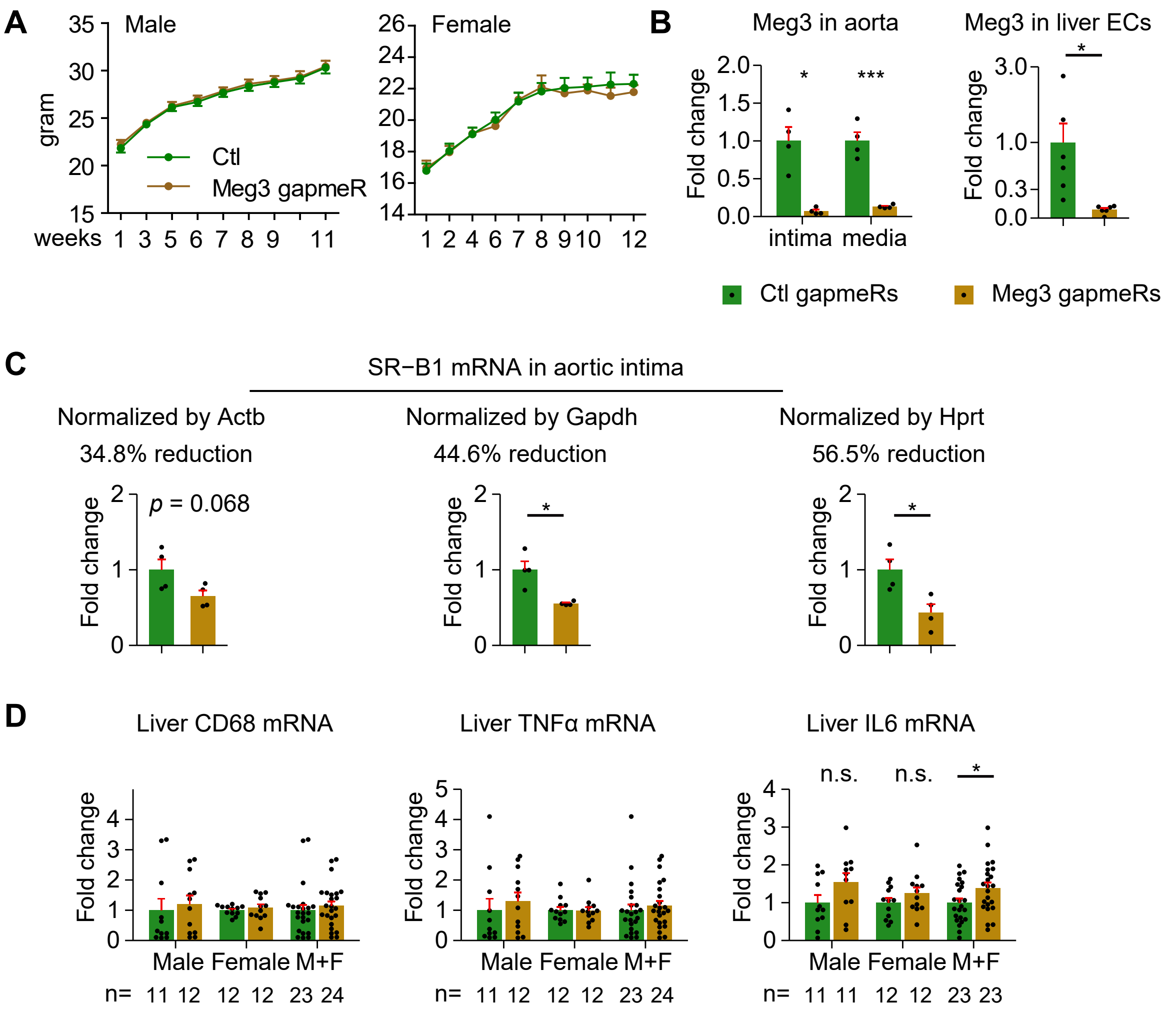

Supplementary Figure S2

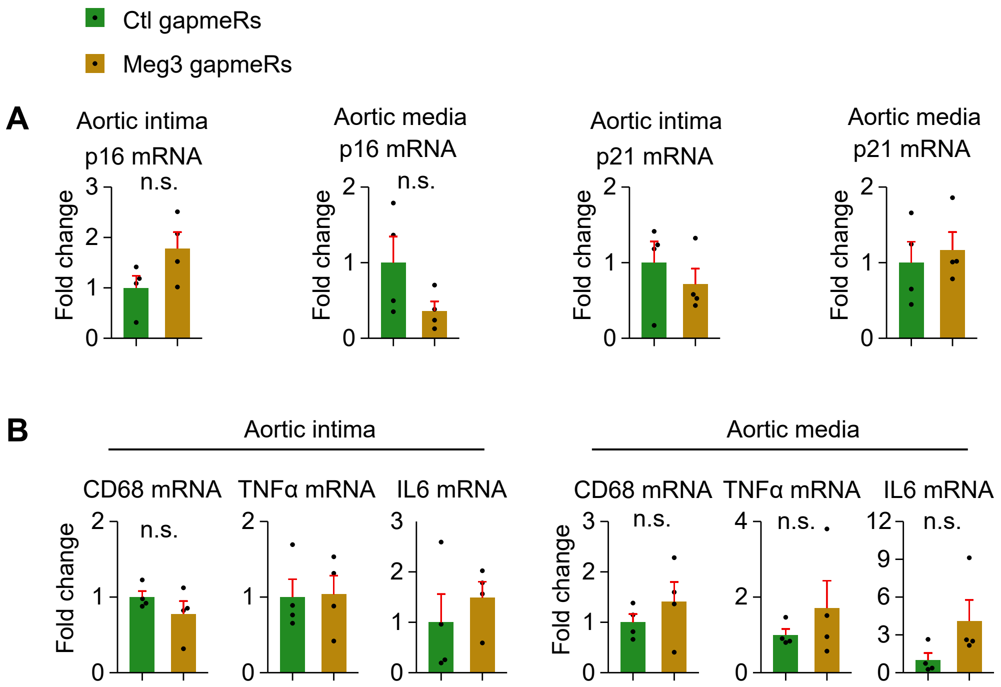

Supplementary Figure S3

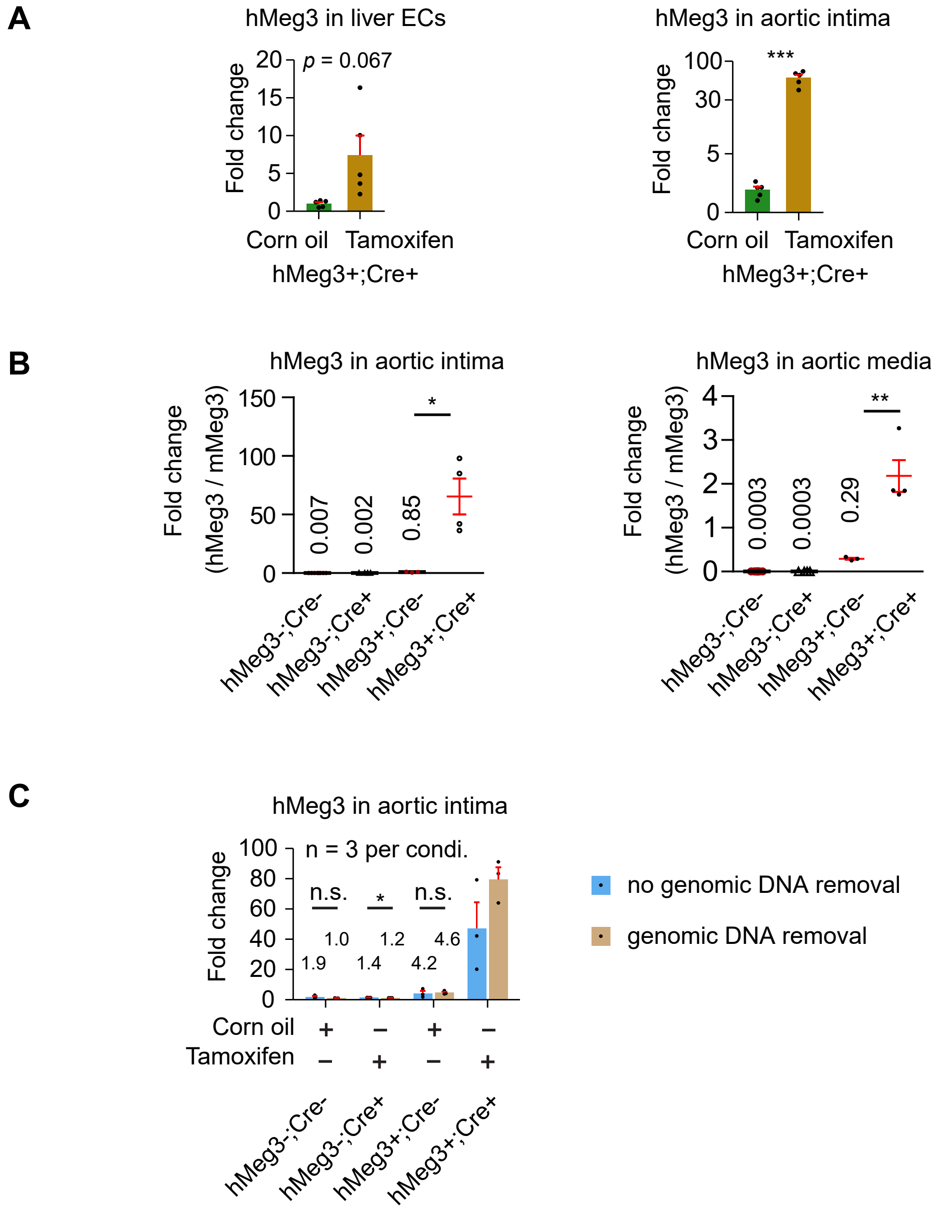

Supplementary Figure S4

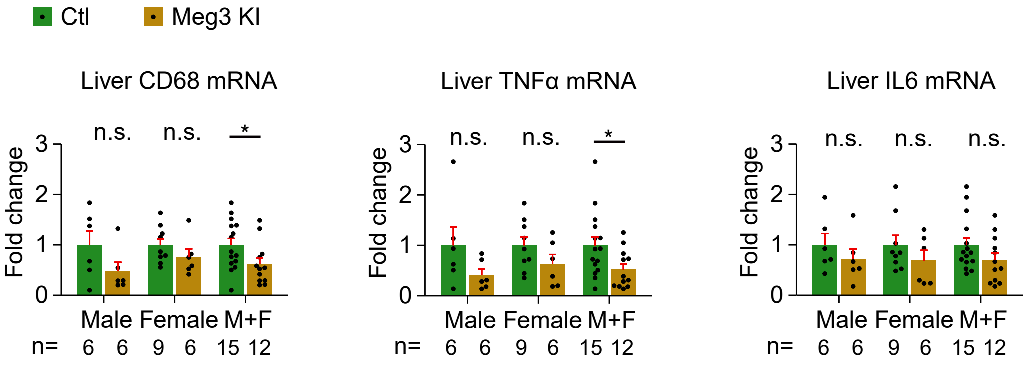

Supplementary Figure S5

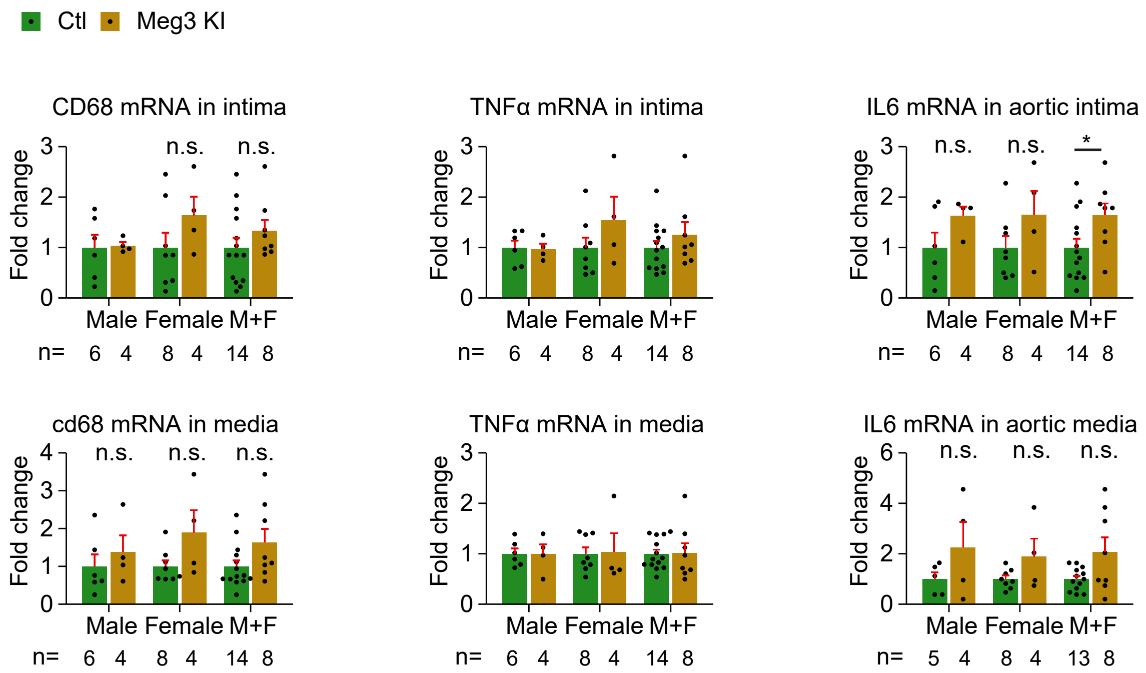

Supplementary Table 1

Gene	Sequences (5’-3’)
hMeg3 forward	GGGCATTAAGCCCTGACCTT
hMeg3 reverse	CCTTGGGGAGGGAAACACTC
mMeg3 forward	TCACCTCCAATTTCCCCTCC
mMeg3 reverse	GCAAGCCAAGCCTTAAACCT
mCDKN1A(p21) forward	CCTGGTGATGTCCGACCTG
mCDKN1A(p21) reverse	CCATGAGCGCATCGCAATC
mCDKN2A(p16) forward	CGCAGGTTCTTGGTCACTGT
mCDKN2A(p16) reverse	TGTTCACGAAAGCCAGAGCG
mCD68 forward	TGTCTGATCTTGCTAGGACCG
mCD68 reverse	GAGAGTAACGGCCTTTTTGTGA
mTNFα forward	CCCTCACACTCAGATCATCTTCT
mTNFα reverse	GCTACGACGTGGGCTACAG
mActb forward	GGCTGTATTCCCCTCCATCG
mActb reverse	CCAGTTGGTAACAATGCCATGT
mIL6 forward	TAGTCCTTCCTACCCCAATTTCC
mIL6 reverse	TTGGTCCTTAGCCACTCCTTC
mGapdh forward	AGGTCGGTGTGAACGGATTTG
mGapdh reverse	TGTAGACCATGTAGTTGAGGTCA
mHprt forward	TCAGTCAACGGGGGACATAAA
mHprt reverse	GGGGCTGTACTGCTTAACCAG
mScarb1 forward	TGTACTGCCTAACATCTTGGTCC
mScarb1 reverse	ACTGTGCGGTTCATAAAAGCA

Supplementary Table 2

gene_id	Meg3 KI	Ctl	log2FoldChange	pvalue	gene_name
ENSMUSG00000064246	945.82	234.72	2.01	0.0000	Chil1
ENSMUSG00000105504	438.61	661.37	-0.59	0.0000	Gbp5
ENSMUSG00000030468	27.35	94.79	-1.78	0.0000	Siglecg
ENSMUSG00000095040	7.14	23.56	-1.74	0.0000	1700001J03Rik
ENSMUSG00000080935	9.45	34.36	-1.84	0.0000	Got2-ps1
ENSMUSG00000007877	8628.40	13450.30	-0.64	0.0000	Tcap
ENSMUSG00000104213	82.50	314.76	-1.93	0.0000	Ighd
ENSMUSG00000030724	56.33	202.31	-1.84	0.0000	Cd19
ENSMUSG00000045657	43.54	12.20	1.82	0.0001	Pcdhb10
ENSMUSG00000074934	255.94	90.33	1.50	0.0001	Grem1
ENSMUSG00000078763	53.93	118.67	-1.13	0.0001	Slfn1
ENSMUSG00000027485	91.73	30.13	1.59	0.0001	Bpifb1
ENSMUSG00000039579	116.73	12.59	3.21	0.0001	Grin3a
ENSMUSG00000028360	115.54	6.85	4.08	0.0001	Slc44a5
ENSMUSG00000034783	7.75	0.00	5.24	0.0002	Cd207
ENSMUSG00000014030	37.63	116.33	-1.62	0.0002	Pax5
ENSMUSG00000005540	23.60	98.54	-2.04	0.0002	Fcer2a
ENSMUSG00000052056	503.31	632.07	-0.33	0.0003	Zfp217
ENSMUSG00000003379	79.35	278.58	-1.81	0.0003	Cd79a
ENSMUSG00000035095	90.05	53.39	0.76	0.0003	Fam167a
ENSMUSG00000049100	196.78	15.96	3.62	0.0003	Pcdh10
ENSMUSG00000067017	218.70	138.66	0.66	0.0003	Capza1-ps1
ENSMUSG00000028020	278.38	44.40	2.65	0.0003	Glrb
ENSMUSG00000040592	77.12	197.42	-1.35	0.0003	Cd79b
ENSMUSG00000040133	522.74	214.95	1.28	0.0004	Gpr176
ENSMUSG00000042474	31.01	105.40	-1.76	0.0004	Fcmr
ENSMUSG00000087008	4.41	16.61	-1.91	0.0004	Gm5530
ENSMUSG00000018924	5.27	77.59	-3.89	0.0004	Alox15
ENSMUSG00000058153	196.22	15.91	3.62	0.0005	Sez6l
ENSMUSG00000060780	109.96	7.28	3.91	0.0005	Lrrtm1
ENSMUSG00000033854	146.95	15.84	3.21	0.0005	Kcnk10
ENSMUSG00000030677	148.28	97.01	0.60	0.0005	Kif22
ENSMUSG00000028194	1280.23	200.89	2.67	0.0006	Ddah1
ENSMUSG00000107152	11.52	1.42	3.04	0.0007	Gm43152
ENSMUSG00000022780	85.51	38.36	1.14	0.0007	Meltf
ENSMUSG00000075334	48.22	5.76	3.08	0.0007	Rprm
ENSMUSG00000029245	179.21	17.43	3.36	0.0007	Epha5
ENSMUSG00000024673	34.59	108.44	-1.64	0.0007	Ms4a1
ENSMUSG00000024399	80.88	180.44	-1.15	0.0008	Ltb
ENSMUSG00000027297	919.16	65.39	3.81	0.0008	Ltk
ENSMUSG00000024907	299.62	31.79	3.24	0.0008	Gal
ENSMUSG00000026581	63.75	194.04	-1.60	0.0008	Sell
ENSMUSG00000039954	661.03	112.59	2.55	0.0008	Stk32a
ENSMUSG00000029378	204.11	3.01	6.07	0.0009	Areg

ENSMUSG00000027524	1266.82	48.81	4.70	0.0010	Edn3
ENSMUSG00000070695	97.95	5.97	4.03	0.0010	Cntnap5a
ENSMUSG00000068606	13.13	42.48	-1.69	0.0010	Gm4841
ENSMUSG00000045136	2499.71	447.03	2.48	0.0010	Tubb2b
ENSMUSG00000055471	62.19	2.21	4.81	0.0010	Alk
ENSMUSG00000058420	588.48	107.10	2.46	0.0011	Syt17
ENSMUSG00000030854	78.49	6.11	3.67	0.0011	Ptpn5
ENSMUSG00000029712	113.17	6.77	4.05	0.0011	Actl6b
ENSMUSG00000020848	1128.58	174.33	2.69	0.0012	Doc2b
ENSMUSG00000050423	5.38	22.46	-2.07	0.0012	Ppp1r3g
ENSMUSG00000055629	159.71	18.99	3.07	0.0012	B4galnt4
ENSMUSG00000025932	622.52	87.67	2.83	0.0012	Eya1
ENSMUSG00000040867	428.60	73.41	2.54	0.0012	Begain
ENSMUSG00000025855	2880.84	524.68	2.46	0.0012	Prkar1b
ENSMUSG00000034683	214.30	3.44	5.97	0.0013	Ppp1r1c
ENSMUSG00000064364	5.76	34.43	-2.56	0.0013	mt-Th
ENSMUSG00000045994	50.48	5.32	3.23	0.0013	B3gat1
ENSMUSG00000026247	247.62	12.23	4.34	0.0014	Ecel1
ENSMUSG00000021062	891.25	135.38	2.72	0.0014	Rab15
ENSMUSG00000109505	9.05	0.25	4.74	0.0014	Gm44677
ENSMUSG00000029608	648.04	36.23	4.16	0.0014	Rph3a
ENSMUSG00000022075	1445.04	1771.95	-0.29	0.0014	Rhobtb2
ENSMUSG00000057716	435.62	65.51	2.73	0.0014	Tmem178b
ENSMUSG00000103411	1.93	10.37	-2.42	0.0014	Gm18300
ENSMUSG00000062991	111.48	12.07	3.20	0.0015	Nrg1
ENSMUSG00000037747	187.84	17.76	3.40	0.0015	Phyhipl
ENSMUSG00000020098	393.03	66.94	2.55	0.0015	Pcbd1
ENSMUSG00000025867	5336.35	934.67	2.51	0.0015	Cplx2
ENSMUSG00000075478	129.61	17.29	2.91	0.0015	Slitrk1
ENSMUSG00000013974	8.66	25.28	-1.52	0.0015	Mcemp1
ENSMUSG00000093789	17.71	0.30	5.71	0.0015	Methig1
ENSMUSG00000032172	538.27	95.90	2.49	0.0015	Olfm2
ENSMUSG00000059412	956.55	186.88	2.36	0.0016	Fxyd2
ENSMUSG00000028967	4297.65	2810.63	0.61	0.0016	Errfi1
ENSMUSG00000025468	97.91	8.34	3.54	0.0016	Caly
ENSMUSG00000000673	23.85	56.57	-1.24	0.0016	Haao
ENSMUSG00000024502	68.69	5.92	3.53	0.0016	Jakmip2
ENSMUSG00000046341	30.79	10.07	1.62	0.0017	Gm11223
ENSMUSG00000111293	7.62	1.04	2.80	0.0017	Gm34006
ENSMUSG00000111854	1.37	8.89	-2.73	0.0017	Gm48740
ENSMUSG00000056258	603.31	100.31	2.59	0.0017	Kcnq3
ENSMUSG00000069515	214.21	399.06	-0.90	0.0017	Lyz1
ENSMUSG00000052581	47.62	5.47	3.12	0.0017	Lrrtm4
ENSMUSG00000022240	304.82	64.43	2.24	0.0017	Ctnnd2
ENSMUSG00000108348	86.37	8.31	3.38	0.0017	Pnma8c
ENSMUSG00000030519	452.67	29.08	3.96	0.0017	Apba2
ENSMUSG00000071753	49.87	8.18	2.60	0.0018	Cdr1os

ENSMUSG00000032181	780.34	120.30	2.70	0.0018	Scg3
ENSMUSG00000099839	11.73	0.00	5.84	0.0018	Gm29374
ENSMUSG00000037979	802.47	148.09	2.44	0.0018	Ccdc92
ENSMUSG00000033214	119.63	13.02	3.20	0.0018	Slitrk5
ENSMUSG00000020598	382.19	50.39	2.92	0.0018	Nrcam
ENSMUSG00000037217	1688.52	282.35	2.58	0.0018	Syn1
ENSMUSG00000047880	16.11	50.19	-1.64	0.0019	Cxcr5
ENSMUSG00000086583	1346.58	1761.33	-0.39	0.0019	Gm15500
ENSMUSG00000030669	6.92	38.54	-2.45	0.0019	Calca
ENSMUSG00000060716	44.11	16.95	1.36	0.0019	Plekhh1
ENSMUSG00000014453	31.65	68.33	-1.10	0.0019	Blk
ENSMUSG00000050751	248.59	28.28	3.13	0.0019	Pgbd5
ENSMUSG00000036502	116.67	15.79	2.89	0.0019	Tmem255a
ENSMUSG00000113136	13.77	28.84	-1.06	0.0020	Gm19951
ENSMUSG00000019852	323.67	46.39	2.80	0.0020	Arfgef3
ENSMUSG00000060314	40.23	5.59	2.87	0.0020	Zfp941
ENSMUSG00000100596	50.78	6.32	3.00	0.0021	Gm29502
ENSMUSG00000028804	31.63	5.95	2.42	0.0021	Csmd2
ENSMUSG00000021301	66.40	9.22	2.85	0.0021	Hecw1
ENSMUSG00000032446	4.04	15.36	-1.86	0.0021	Eomes
ENSMUSG00000027438	1531.08	260.88	2.55	0.0021	Napb
ENSMUSG00000042734	854.52	414.66	1.04	0.0022	Ttc9
ENSMUSG00000024524	390.12	61.94	2.65	0.0022	Gnal
ENSMUSG00000028051	228.91	31.06	2.88	0.0022	Hcn3
ENSMUSG00000032204	10.37	24.63	-1.22	0.0022	Aqp9
ENSMUSG00000037548	66.58	138.12	-1.05	0.0022	H2-DMb2
ENSMUSG00000011589	165.09	22.66	2.86	0.0022	Fsd1
ENSMUSG00000033676	123.53	10.27	3.59	0.0022	Gabrb3
ENSMUSG00000018899	1364.25	1820.11	-0.42	0.0023	Irf1
ENSMUSG00000063239	23.29	1.23	4.18	0.0023	Grm4
ENSMUSG00000015222	673.05	120.62	2.48	0.0023	Map2
ENSMUSG00000007944	102.02	11.47	3.15	0.0024	Ttc9b
ENSMUSG00000040901	40.54	1.64	4.62	0.0024	Kcnk18
ENSMUSG00000028354	53.99	11.83	2.19	0.0024	Fmn2
ENSMUSG00000051243	1217.13	343.46	1.83	0.0024	Islr2
ENSMUSG00000022021	71.45	47.20	0.60	0.0024	Diaph3
ENSMUSG00000044117	305.34	18.95	4.01	0.0024	Bmerb1
ENSMUSG00000003863	441.33	88.92	2.31	0.0025	Ppfia3
ENSMUSG00000019796	1076.68	185.81	2.53	0.0025	Lrp11
ENSMUSG00000001517	188.74	138.78	0.44	0.0025	Foxm1
ENSMUSG00000019874	425.41	63.52	2.74	0.0026	Fabp7
ENSMUSG00000040258	55.71	11.60	2.25	0.0026	Nxph4
ENSMUSG00000024109	171.58	21.54	2.99	0.0026	Nrxn1
ENSMUSG00000120458	1.34	7.87	-2.41	0.0027	
ENSMUSG00000032076	1178.42	368.40	1.68	0.0027	Cadm1
ENSMUSG00000021198	83.18	13.37	2.64	0.0027	Unc79
ENSMUSG00000026018	94.46	21.59	2.13	0.0027	Ica1l

ENSMUSG00000048483	60.19	5.56	3.43	0.0027	Zdhhc22
ENSMUSG00000097322	10.91	2.22	2.28	0.0027	A530083I20Rik
ENSMUSG00000039419	348.18	52.97	2.71	0.0028	Cntnap2
ENSMUSG00000026764	970.22	201.35	2.27	0.0028	Kif5c
ENSMUSG00000003153	1164.71	235.06	2.31	0.0028	Slc2a3
ENSMUSG00000067889	390.95	70.05	2.48	0.0028	Sptbn2
ENSMUSG00000056290	42.59	100.06	-1.23	0.0028	Ms4a4b
ENSMUSG00000054072	591.35	1193.07	-1.01	0.0029	Iigp1
ENSMUSG00000029126	2742.11	770.30	1.83	0.0029	Nsg1
ENSMUSG00000025318	459.41	32.62	3.81	0.0029	Jph3
ENSMUSG00000074892	25.81	2.62	3.28	0.0029	B3galt5
ENSMUSG00000109904	360.75	242.07	0.57	0.0030	Gm45819
ENSMUSG00000063506	249.50	116.51	1.09	0.0031	Arhgap22
ENSMUSG00000095794	1.21	11.05	-3.26	0.0031	Igkv6-17
ENSMUSG00000043460	120.80	11.34	3.41	0.0031	Elfn2
ENSMUSG00000113216	43.95	11.59	1.92	0.0031	Gm40841
ENSMUSG00000022208	1099.69	224.51	2.29	0.0032	Jph4
ENSMUSG00000029875	48.84	10.63	2.19	0.0032	Ccdc184
ENSMUSG00000034579	102.67	19.41	2.40	0.0032	Pla2g3
ENSMUSG00000032269	4631.75	146.92	4.98	0.0033	Htr3a
ENSMUSG00000031760	485.43	90.43	2.43	0.0033	Mt3
ENSMUSG00000044528	150.09	24.59	2.61	0.0033	Tram1l1
ENSMUSG00000028137	980.66	95.65	3.36	0.0033	Celf3
ENSMUSG00000088008	11.55	24.43	-1.10	0.0033	Gm25492
ENSMUSG00000043670	195.65	24.12	3.02	0.0034	Diras1
ENSMUSG00000035042	77.85	131.23	-0.75	0.0034	Ccl5
ENSMUSG00000049670	548.09	137.49	1.99	0.0034	Morn4
ENSMUSG00000023033	101.85	16.38	2.63	0.0034	Scn8a
ENSMUSG00000039716	125.17	24.93	2.33	0.0034	Dock3
ENSMUSG00000087620	17.98	0.49	5.05	0.0036	5330434G04Rik
ENSMUSG00000047261	2337.89	131.96	4.15	0.0036	Gap43
ENSMUSG00000022658	723.19	60.35	3.58	0.0036	Tagln3
ENSMUSG00000061451	648.50	188.53	1.78	0.0037	Tmem151a
ENSMUSG00000050132	208.98	44.08	2.24	0.0037	Sarm1
ENSMUSG00000039126	1861.92	568.79	1.71	0.0037	Prune2
ENSMUSG00000024771	14.13	0.24	5.39	0.0038	Lipk
ENSMUSG00000001120	2375.58	679.62	1.81	0.0038	Pcbp3
ENSMUSG00000028786	11.53	0.00	5.82	0.0038	Tmem54
ENSMUSG00000094162	34.06	116.57	-1.78	0.0038	Ccl19-ps3
ENSMUSG00000063063	369.25	24.38	3.92	0.0038	Ctnna2
ENSMUSG00000039860	108.25	11.07	3.29	0.0038	Srrm3
ENSMUSG00000043463	134.23	47.22	1.51	0.0038	Rab9b
ENSMUSG00000046204	108.29	10.73	3.33	0.0038	Pnma2
ENSMUSG00000036510	95.26	1.56	5.92	0.0038	Cdh8
ENSMUSG00000075122	161.67	102.98	0.65	0.0038	Cd80
ENSMUSG00000026188	89.13	20.57	2.11	0.0038	Tmem169
ENSMUSG00000021087	4014.14	357.45	3.49	0.0039	Rtn1

ENSMUSG00000036295	239.34	50.78	2.24	0.0039	Lrrn3
ENSMUSG00000039838	301.91	14.98	4.33	0.0039	Slc45a1
ENSMUSG00000092083	32.05	1.55	4.34	0.0039	Kcnb2
ENSMUSG00000085007	65.77	12.70	2.37	0.0039	Smim43
ENSMUSG00000114515	15.02	33.36	-1.15	0.0039	Aldoa
ENSMUSG00000120754	21.29	42.53	-1.00	0.0039	
ENSMUSG00000023011	2212.63	447.13	2.31	0.0040	Faim2
ENSMUSG00000024500	812.57	52.02	3.96	0.0040	Ppp2r2b
ENSMUSG00000037541	143.46	37.24	1.94	0.0040	Shank2
ENSMUSG00000033707	45.87	13.56	1.76	0.0040	Lrrc24
ENSMUSG00000026616	10.80	26.56	-1.29	0.0041	Cr2
ENSMUSG00000085008	37.21	1.72	4.45	0.0041	Dbhos
ENSMUSG00000005705	55.76	9.85	2.51	0.0042	Agrp
ENSMUSG00000045733	318.39	90.92	1.81	0.0043	Sprn
ENSMUSG00000039717	59.62	6.25	3.24	0.0043	Ralyl
ENSMUSG00000042540	0.00	3.82	-4.54	0.0043	Acot5
ENSMUSG00000069763	635.82	502.46	0.34	0.0043	Tmem100
ENSMUSG00000042751	610.36	167.50	1.86	0.0043	Nmnat2
ENSMUSG00000060240	1354.33	334.51	2.02	0.0044	Cend1
ENSMUSG00000011751	159.09	26.70	2.57	0.0045	Sptbn4
ENSMUSG00000096351	40.94	6.42	2.66	0.0045	Samd11
ENSMUSG00000038916	174.44	11.25	3.95	0.0046	Soga3
ENSMUSG00000117351	0.66	5.79	-3.12	0.0046	Gm31615
ENSMUSG00000037681	125.75	37.75	1.73	0.0046	Esyt3
ENSMUSG00000054459	126.31	12.84	3.30	0.0047	Vsnl1
ENSMUSG00000033597	752.82	171.48	2.13	0.0047	Caskin1
ENSMUSG00000034127	840.56	76.96	3.45	0.0048	Tspan8
ENSMUSG00000044199	95.12	156.24	-0.71	0.0048	S1pr4
ENSMUSG00000042532	212.27	44.58	2.25	0.0048	Golga7b
ENSMUSG00000029223	10546.56	2973.64	1.83	0.0048	Uchl1
ENSMUSG00000041245	30.28	4.70	2.69	0.0048	Wnk3
ENSMUSG00000022619	1584.35	112.30	3.82	0.0048	Mapk8ip2
ENSMUSG00000052105	359.82	132.38	1.44	0.0049	Mtcl1
ENSMUSG00000046338	63.65	11.79	2.43	0.0049	Gpat2
ENSMUSG00000038893	577.94	767.80	-0.41	0.0049	Fam117a
ENSMUSG00000055567	187.74	11.78	3.99	0.0049	Unc80
ENSMUSG00000033510	97.73	10.59	3.20	0.0049	Otud7a
ENSMUSG00000102051	1.73	10.51	-2.61	0.0049	Ly6a2
ENSMUSG00000039059	39.11	4.92	2.99	0.0049	Hrh3
ENSMUSG00000034156	160.13	44.88	1.83	0.0049	Tspoap1
ENSMUSG00000048978	753.69	42.35	4.15	0.0049	Nrsn1
ENSMUSG00000031872	397.55	117.10	1.76	0.0050	Bean1
ENSMUSG00000032011	5047.60	1962.41	1.36	0.0050	Thy1
ENSMUSG00000057182	144.67	25.49	2.50	0.0050	Scn3a
ENSMUSG00000025189	82.86	12.03	2.78	0.0051	Cnnm1
ENSMUSG00000022025	199.50	33.22	2.59	0.0051	Cnmd
ENSMUSG00000027201	422.39	319.62	0.40	0.0051	Myef2

ENSMUSG00000045284	69.12	15.70	2.14	0.0051	Dcaf12l1
ENSMUSG00000032262	146.12	48.68	1.59	0.0051	Elovl4
ENSMUSG00000104850	3.80	11.68	-1.60	0.0051	Gm42901
ENSMUSG00000031847	34.12	16.16	1.07	0.0051	1700030J22Rik
ENSMUSG00000000792	147.35	26.86	2.45	0.0052	Slc5a5
ENSMUSG00000048895	184.59	53.39	1.79	0.0052	Cdk5r1
ENSMUSG00000025789	75.66	43.55	0.80	0.0052	St8sia2
ENSMUSG00000086881	17.32	32.90	-0.94	0.0052	Gm13594
ENSMUSG00000079157	160.16	11.41	3.80	0.0053	Nalf1
ENSMUSG00000063919	155.61	43.27	1.85	0.0053	Srrm4
ENSMUSG00000027994	484.70	349.63	0.47	0.0053	Mcub
ENSMUSG00000049281	626.43	185.55	1.75	0.0053	Scn3b
ENSMUSG00000024261	1902.09	109.81	4.11	0.0053	Syt4
ENSMUSG00000030683	552.37	104.57	2.40	0.0053	Sez6l2
ENSMUSG00000037725	104.08	61.10	0.76	0.0053	Ckap2
ENSMUSG00000062380	8823.57	887.51	3.31	0.0054	Tubb3
ENSMUSG00000049511	1062.36	694.98	0.61	0.0054	Htr1b
ENSMUSG00000020340	1343.18	448.12	1.58	0.0054	Cyfip2
ENSMUSG00000070304	630.05	147.00	2.10	0.0054	Scn2b
ENSMUSG00000048385	51.42	5.30	3.28	0.0054	Scrt1
ENSMUSG00000058740	653.68	146.33	2.16	0.0055	Kcnt1
ENSMUSG00000008590	809.55	28.20	4.84	0.0055	Htr3b
ENSMUSG00000056071	63.53	130.86	-1.04	0.0055	S100a9
ENSMUSG00000038791	3.99	17.20	-2.08	0.0055	Scgb3a2
ENSMUSG00000019429	82.08	6.38	3.68	0.0055	Ffar3
ENSMUSG00000036564	9254.77	2475.46	1.90	0.0055	Ndrg4
ENSMUSG00000031202	145.00	50.39	1.52	0.0056	Rab39b
ENSMUSG00000025576	97.20	14.57	2.73	0.0056	Rbfox3
ENSMUSG00000056004	398.85	176.32	1.18	0.0056	Elapor2
ENSMUSG00000030283	205.02	30.17	2.77	0.0057	St8sia1
ENSMUSG00000000120	10736.44	638.98	4.07	0.0057	Ngfr
ENSMUSG00000043909	830.43	695.18	0.26	0.0057	Trp53bp1
ENSMUSG00000032890	414.11	88.40	2.23	0.0057	Rims3
ENSMUSG00000000627	223.39	48.02	2.22	0.0057	Sema4f
ENSMUSG00000070348	1132.67	925.56	0.29	0.0057	Ccnd1
ENSMUSG00000044499	55.26	4.79	3.55	0.0057	Hs3st5
ENSMUSG00000061171	127.59	78.95	0.68	0.0057	Slc38a11
ENSMUSG00000079598	85.52	14.94	2.51	0.0058	Clec2l
ENSMUSG00000007021	1623.54	179.72	3.17	0.0058	Syngr3
ENSMUSG00000097767	356.95	67.33	2.41	0.0058	Miat
ENSMUSG00000024044	1484.98	480.44	1.63	0.0059	Epb41l3
ENSMUSG00000028337	604.66	206.22	1.55	0.0059	Coro2a
ENSMUSG00000047786	587.39	115.26	2.35	0.0059	Lix1
ENSMUSG00000006711	92.17	7.70	3.58	0.0059	D130043K22Rik
ENSMUSG00000040495	23.25	2.47	3.23	0.0059	Chrm4
ENSMUSG00000046793	43.57	2.13	4.35	0.0060	Gpr61
ENSMUSG00000027612	80.31	8.65	3.21	0.0060	Mmp24

ENSMUSG00000062588	13.96	31.31	-1.18	0.0060	Gm6104
ENSMUSG00000052852	1655.70	436.48	1.92	0.0061	Reep1
ENSMUSG00000052572	475.11	133.03	1.84	0.0061	Dlg2
ENSMUSG00000120584	1.01	6.42	-2.80	0.0061	Gm26641
ENSMUSG00000023439	363.91	27.55	3.72	0.0061	Gnb3
ENSMUSG00000040035	1515.33	122.58	3.63	0.0062	Disp2
ENSMUSG00000020297	2286.60	198.09	3.53	0.0062	Nsg2
ENSMUSG00000021303	236.74	70.02	1.76	0.0062	Gng4
ENSMUSG00000025013	53.27	7.92	2.75	0.0062	Tll2
ENSMUSG00000042401	343.81	27.70	3.64	0.0062	Crtac1
ENSMUSG00000059857	290.66	85.85	1.76	0.0062	Ntng1
ENSMUSG00000095407	48.66	9.60	2.33	0.0062	Tmem200c
ENSMUSG00000074997	3.01	0.00	3.89	0.0063	Pin1rt1
ENSMUSG00000015981	220.15	60.29	1.87	0.0063	Stk32c
ENSMUSG00000029757	266.46	23.27	3.52	0.0063	Dync1i1
ENSMUSG00000118661	10.06	31.80	-1.63	0.0063	Muc6
ENSMUSG00000038193	2239.37	637.10	1.81	0.0063	Hand2
ENSMUSG00000033569	76.34	8.03	3.25	0.0063	Adgrb3
ENSMUSG00000040183	235.16	58.10	2.02	0.0063	Ankrd6
ENSMUSG00000033615	1939.68	104.32	4.22	0.0064	Cplx1
ENSMUSG00000031258	21.63	41.55	-0.93	0.0064	Xkrx
ENSMUSG00000034028	27.05	46.72	-0.81	0.0064	Cd226
ENSMUSG00000082229	89.53	7.81	3.52	0.0064	Nap1l2
ENSMUSG00000050822	898.26	64.39	3.80	0.0064	Slc29a4
ENSMUSG00000019590	8575.58	3543.48	1.27	0.0064	Cyb561
ENSMUSG00000026204	2092.47	265.47	2.98	0.0065	Ptprn
ENSMUSG00000055733	169.62	52.32	1.69	0.0065	Nap1l3
ENSMUSG00000014602	4497.96	1337.88	1.75	0.0065	Kif1a
ENSMUSG00000007946	965.39	51.40	4.23	0.0065	Phox2a
ENSMUSG00000020704	484.99	32.37	3.90	0.0067	Asic2
ENSMUSG00000037580	1173.75	352.31	1.74	0.0067	Gch1
ENSMUSG00000042388	229.63	33.97	2.75	0.0067	Dlgap3
ENSMUSG00000062444	624.57	50.68	3.62	0.0067	Ap3b2
ENSMUSG00000031391	2030.56	661.16	1.62	0.0067	L1cam
ENSMUSG00000030110	2953.48	270.22	3.45	0.0067	Ret
ENSMUSG00000114488	7.35	0.55	3.71	0.0068	Gm5802
ENSMUSG00000018451	563.94	134.03	2.07	0.0068	6330403K07Rik
ENSMUSG00000039372	181.49	12.97	3.80	0.0068	Marchf4
ENSMUSG00000083443	68.80	97.32	-0.51	0.0069	Gm15519
ENSMUSG00000065460	0.86	5.65	-2.68	0.0069	Mir133a-2
ENSMUSG00000016346	600.48	37.82	3.99	0.0069	Kcnq2
ENSMUSG00000025432	1619.98	151.39	3.42	0.0070	Avil
ENSMUSG00000023972	268.25	172.55	0.64	0.0070	Ptk7
ENSMUSG00000051497	21.27	2.04	3.40	0.0070	Kcnj16
ENSMUSG00000068105	12.66	38.89	-1.60	0.0070	Tnfrsf13c
ENSMUSG00000040412	53.70	7.31	2.86	0.0070	Elapor1
ENSMUSG00000043068	476.43	120.53	1.98	0.0070	Fam89a

ENSMUSG00000026452	947.44	98.36	3.27	0.0070	Syt2
ENSMUSG00000033491	74.49	19.80	1.91	0.0070	Prss35
ENSMUSG00000021314	577.98	60.85	3.25	0.0070	Amph
ENSMUSG00000110710	220.84	41.49	2.41	0.0070	C78859
ENSMUSG00000042750	869.68	55.15	3.98	0.0070	Bex2
ENSMUSG00000029120	794.71	58.27	3.77	0.0070	Ppp2r2c
ENSMUSG00000048814	440.19	139.01	1.66	0.0070	Lonrf2
ENSMUSG00000005640	367.84	38.32	3.26	0.0071	Insrr
ENSMUSG00000034324	129.44	21.57	2.59	0.0071	Tmem132c
ENSMUSG00000006930	453.70	139.08	1.70	0.0071	Hap1
ENSMUSG00000058297	6690.96	1902.44	1.81	0.0071	Spock2
ENSMUSG00000097885	150.92	190.04	-0.34	0.0071	5031434O11Rik
ENSMUSG00000023484	6200.60	401.60	3.95	0.0071	Prph
ENSMUSG00000034818	92.74	33.59	1.46	0.0071	Celf5
ENSMUSG00000102577	11.44	25.13	-1.15	0.0072	Gm37969
ENSMUSG00000055430	1097.92	302.97	1.86	0.0072	Nap1l5
ENSMUSG00000037922	60.45	102.55	-0.75	0.0073	Bank1
ENSMUSG00000049353	1.17	7.45	-2.56	0.0073	Rd3
ENSMUSG00000010066	178.75	43.25	2.05	0.0074	Cacna2d2
ENSMUSG00000029636	338.54	114.29	1.57	0.0074	Wasf3
ENSMUSG00000074657	2473.93	381.48	2.70	0.0075	Kif5a
ENSMUSG00000036699	261.49	41.35	2.66	0.0075	Zcchc12
ENSMUSG00000022376	62.99	26.07	1.27	0.0075	Adcy8
ENSMUSG00000058672	5250.09	2234.88	1.23	0.0075	Tubb2a
ENSMUSG00000016763	444.86	130.69	1.77	0.0075	Scube1
ENSMUSG00000054162	478.00	33.92	3.82	0.0075	Spock3
ENSMUSG00000097039	215.04	110.68	0.96	0.0076	Pvt1
ENSMUSG00000049176	52.23	9.32	2.47	0.0076	Frmpd4
ENSMUSG00000028965	93.20	60.17	0.62	0.0076	Tnfrsf9
ENSMUSG00000027500	5152.55	616.94	3.06	0.0076	Stmn2
ENSMUSG00000086361	0.27	3.75	-3.77	0.0076	Gm6569
ENSMUSG00000025870	433.18	331.54	0.38	0.0077	Arl10
ENSMUSG00000028782	358.05	99.51	1.85	0.0077	Adgrb2
ENSMUSG00000084235	38.41	20.02	0.96	0.0077	Gm15421
ENSMUSG00000108847	3.85	0.00	4.22	0.0078	B830042I05Rik
ENSMUSG00000027350	2520.65	166.80	3.92	0.0078	Chgb
ENSMUSG00000053825	100.18	23.40	2.10	0.0078	Ppfia2
ENSMUSG00000021587	154.34	26.21	2.56	0.0078	Pcsk1
ENSMUSG00000049122	59.20	15.52	1.93	0.0078	Frmd3
ENSMUSG00000044453	6.25	19.60	-1.66	0.0078	Ffar1
ENSMUSG00000056222	421.99	29.89	3.82	0.0078	Spock1
ENSMUSG00000022883	247.83	137.38	0.85	0.0078	Robo1
ENSMUSG00000042763	182.94	40.69	2.17	0.0078	Maneal
ENSMUSG00000004267	2640.77	850.64	1.63	0.0079	Eno2
ENSMUSG00000060924	109.78	52.78	1.06	0.0079	Csmd1
ENSMUSG00000069540	4.67	13.82	-1.54	0.0079	Gm4925
ENSMUSG00000068744	152.23	99.20	0.61	0.0079	Psrc1

ENSMUSG00000028832	1664.70	748.37	1.15	0.0080	Stmn1
ENSMUSG00000029334	51.34	20.53	1.32	0.0080	Prkg2
ENSMUSG00000108049	36.39	18.30	1.00	0.0080	Gm44168
ENSMUSG00000030000	656.17	54.67	3.58	0.0080	Add2
ENSMUSG00000085095	18.06	34.96	-0.97	0.0080	Gm15635
ENSMUSG00000032281	495.28	138.50	1.84	0.0080	Acsbg1
ENSMUSG00000036304	594.78	340.98	0.80	0.0080	Zdhhc23
ENSMUSG00000032908	488.03	40.54	3.59	0.0081	Sgpp2
ENSMUSG00000063646	44.51	8.50	2.39	0.0081	Jakmip1
ENSMUSG00000024064	52.97	7.62	2.80	0.0081	Galnt14
ENSMUSG00000041078	35.89	2.78	3.66	0.0082	Grid1
ENSMUSG00000037944	44.31	81.10	-0.86	0.0083	Ccr7
ENSMUSG00000033256	655.96	224.90	1.54	0.0083	Shf
ENSMUSG00000036067	237.13	77.79	1.61	0.0083	Slc2a6
ENSMUSG00000053046	292.21	58.81	2.31	0.0083	Brsk2
ENSMUSG00000037428	822.27	47.28	4.12	0.0083	Vgf
ENSMUSG00000010086	265.96	46.18	2.52	0.0083	Rnf112
ENSMUSG00000082248	4.23	13.02	-1.58	0.0084	Gm13161
ENSMUSG00000028972	1.27	6.41	-2.47	0.0084	Car6
ENSMUSG00000085925	32.22	4.96	2.70	0.0084	Rtl1
ENSMUSG00000054679	47.73	5.42	3.12	0.0084	Srsf12
ENSMUSG00000025927	242.55	52.76	2.20	0.0085	Tfap2b
ENSMUSG00000019831	208.85	85.92	1.28	0.0085	Wasf1
ENSMUSG00000027581	2327.97	170.92	3.77	0.0085	Stmn3
ENSMUSG00000044628	163.77	28.93	2.50	0.0085	Rnf208
ENSMUSG00000068327	442.30	24.82	4.15	0.0085	Tlx2
ENSMUSG00000120285	13.07	0.49	4.59	0.0085	
ENSMUSG00000033061	166.36	13.45	3.63	0.0086	Resp18
ENSMUSG00000010825	48.33	4.23	3.49	0.0086	Grid2ip
ENSMUSG00000030268	405.75	140.43	1.53	0.0086	Bcat1
ENSMUSG00000100510	977.69	341.80	1.52	0.0086	Hand2os1
ENSMUSG00000051682	21.64	45.29	-1.05	0.0086	Treml4
ENSMUSG00000020182	5231.47	334.61	3.97	0.0086	Ddc
ENSMUSG00000039096	602.34	732.16	-0.28	0.0086	Rsad1
ENSMUSG00000022212	90.62	19.60	2.21	0.0086	Cpne6
ENSMUSG00000036731	28.85	6.10	2.22	0.0087	Cysrt1
ENSMUSG00000034799	563.96	196.80	1.52	0.0087	Unc13a
ENSMUSG00000113926	0.22	3.34	-3.62	0.0087	Gm3565
ENSMUSG00000029168	1010.30	277.56	1.86	0.0087	Dpysl5
ENSMUSG00000045318	295.48	109.94	1.42	0.0087	Adra2c
ENSMUSG00000055368	11914.61	752.94	3.98	0.0088	Slc6a2
ENSMUSG00000081225	161.46	7.65	4.39	0.0088	Cyp2j12
ENSMUSG00000029657	2725.73	2049.29	0.41	0.0088	Hsph1
ENSMUSG00000025777	314.14	30.92	3.34	0.0088	Gdap1
ENSMUSG00000012520	598.20	38.15	3.97	0.0088	Phox2b
ENSMUSG00000029121	736.48	76.74	3.26	0.0088	Crmp1
ENSMUSG00000023236	502.66	45.65	3.46	0.0089	Scg5

ENSMUSG00000020396	925.26	61.97	3.90	0.0089	Nefh
ENSMUSG00000076937	15.28	49.42	-1.70	0.0089	Iglc2
ENSMUSG00000039485	1359.53	490.80	1.47	0.0090	Tspyl4
ENSMUSG00000020135	864.26	237.54	1.86	0.0090	Apc2
ENSMUSG00000097789	172.00	66.25	1.38	0.0090	Gm2115
ENSMUSG00000025037	3052.83	1219.83	1.32	0.0090	Maoa
ENSMUSG00000034522	2027.66	2565.90	-0.34	0.0091	Zfp395
ENSMUSG00000034891	333.63	18.75	4.15	0.0092	Sncb
ENSMUSG00000025272	429.61	170.98	1.33	0.0092	Tro
ENSMUSG00000050473	88.85	10.12	3.12	0.0092	Slc35d3
ENSMUSG00000043366	192.80	37.85	2.35	0.0092	Olfr78
ENSMUSG00000031428	1431.14	222.91	2.68	0.0092	Zcchc18
ENSMUSG00000021032	157.20	49.94	1.65	0.0092	Ngb
ENSMUSG00000045083	24.19	2.23	3.44	0.0092	Lingo2
ENSMUSG00000004612	23.82	41.12	-0.77	0.0093	Nkg7
ENSMUSG00000074006	946.67	64.71	3.87	0.0093	Omp
ENSMUSG00000010154	196.87	47.69	2.05	0.0093	Spire2
ENSMUSG00000074715	24.85	13.78	0.86	0.0093	Ccl28
ENSMUSG00000032017	38.90	3.83	3.34	0.0093	Grik4
ENSMUSG00000055022	513.12	60.93	3.07	0.0094	Cntn1
ENSMUSG00000047420	214.86	126.85	0.76	0.0094	Fam180a
ENSMUSG00000075316	213.68	15.58	3.78	0.0095	Scn9a
ENSMUSG00000039037	301.11	60.26	2.32	0.0095	St6galnac5
ENSMUSG00000050875	573.48	78.68	2.86	0.0096	Minar2
ENSMUSG00000045763	2692.94	1154.47	1.22	0.0096	Basp1
ENSMUSG00000062252	175.37	17.40	3.33	0.0096	Lhfpl5
ENSMUSG00000107336	10.96	21.89	-0.98	0.0096	Gm43461
ENSMUSG00000094652	0.27	7.50	-4.78	0.0097	Ighv1-42
ENSMUSG00000048070	1705.20	95.08	4.16	0.0097	Pirt
ENSMUSG00000032589	354.08	101.34	1.80	0.0097	Bsn
ENSMUSG00000107373	27.99	3.04	3.20	0.0098	Gm42668
ENSMUSG00000050640	122.46	21.45	2.51	0.0098	Tmem150c
ENSMUSG00000028270	1613.24	2056.17	-0.35	0.0098	Gbp2
ENSMUSG00000021209	110.47	44.79	1.30	0.0098	Ppp4r4
ENSMUSG00000053192	1030.73	370.79	1.47	0.0098	Mllt11
ENSMUSG00000029053	322.25	97.55	1.72	0.0099	Prkcz
ENSMUSG00000026605	79.30	51.99	0.61	0.0099	Cenpf
ENSMUSG00000034145	93.20	10.37	3.16	0.0099	Tmem63c
ENSMUSG00000005045	982.37	157.41	2.64	0.0099	Chd5
ENSMUSG00000041544	39.79	5.96	2.72	0.0099	Disp3
ENSMUSG00000071847	1021.45	782.35	0.38	0.0100	Apcdd1
ENSMUSG00000036766	697.19	72.58	3.26	0.0100	Dner
ENSMUSG00000024897	3412.09	1269.02	1.43	0.0100	Apba1
ENSMUSG00000037202	10.53	24.76	-1.20	0.0101	Prf1
ENSMUSG00000069072	754.49	57.06	3.72	0.0101	Slc7a14
ENSMUSG00000030270	131.68	18.76	2.81	0.0101	Cpne9
ENSMUSG00000037335	606.69	41.86	3.86	0.0101	Hand1

ENSMUSG00000000889	13175.28	690.50	4.25	0.0101	Dbh
ENSMUSG00000057606	495.08	54.64	3.18	0.0101	Colq
ENSMUSG00000022456	346.60	93.86	1.88	0.0101	Septin3
ENSMUSG00000060962	497.07	46.88	3.41	0.0101	Dmkn
ENSMUSG00000055202	67.95	41.17	0.72	0.0102	Zfp811
ENSMUSG00000032291	47.10	3.86	3.59	0.0102	Crabp1
ENSMUSG00000017390	780.05	119.76	2.70	0.0102	Aldoc
ENSMUSG00000021700	1281.09	87.66	3.87	0.0103	Rab3c
ENSMUSG00000028251	525.26	653.04	-0.31	0.0103	Tstd3
ENSMUSG00000022044	319.36	109.63	1.54	0.0104	Stmn4
ENSMUSG00000035105	390.94	557.82	-0.51	0.0104	Egln3
ENSMUSG00000010505	55.84	4.45	3.64	0.0105	Myt1
ENSMUSG00000021066	494.49	153.70	1.69	0.0105	Atl1
ENSMUSG00000040276	648.54	66.31	3.29	0.0105	Pacsin1
ENSMUSG00000118426	40.85	2.53	3.99	0.0105	Gm2102
ENSMUSG00000028524	414.25	165.91	1.32	0.0105	Sgip1
ENSMUSG00000027966	278.32	77.29	1.85	0.0106	Col11a1
ENSMUSG00000028873	138.79	92.90	0.57	0.0106	Cdca8
ENSMUSG00000022496	6.83	15.46	-1.19	0.0107	Tnfrsf17
ENSMUSG00000044912	33.00	3.82	3.09	0.0107	Syt16
ENSMUSG00000087648	2.71	10.21	-1.97	0.0108	E130018N17Rik
ENSMUSG00000039278	2352.28	167.15	3.81	0.0108	Pcsk1n
ENSMUSG00000026576	5010.28	1668.96	1.59	0.0108	Atp1b1
ENSMUSG00000087157	16.04	0.74	4.36	0.0108	Sox5os2
ENSMUSG00000071658	677.12	60.49	3.48	0.0109	Gng3
ENSMUSG00000035085	187.53	287.72	-0.62	0.0109	1700020L24Rik
ENSMUSG00000025094	1822.75	271.47	2.75	0.0109	Slc18a2
ENSMUSG00000066189	113.42	4.14	4.77	0.0109	Cacng3
ENSMUSG00000024846	88.73	49.19	0.84	0.0110	Cst6
ENSMUSG00000020178	220.52	358.45	-0.70	0.0110	Adora2a
ENSMUSG00000042269	7.43	0.57	3.74	0.0110	Cibar2
ENSMUSG00000028289	182.34	123.91	0.55	0.0110	Epha7
ENSMUSG00000030651	1.96	12.12	-2.62	0.0111	Art2b
ENSMUSG00000064366	30.12	95.99	-1.68	0.0111	mt-Tl2
ENSMUSG00000031284	250.23	99.24	1.33	0.0111	Pak3
ENSMUSG00000090071	545.30	37.77	3.85	0.0111	Cdk5r2
ENSMUSG00000069814	141.55	41.95	1.75	0.0112	Ccdc92b
ENSMUSG00000049036	44.35	14.64	1.59	0.0112	Tmem121
ENSMUSG00000064361	7.26	28.02	-1.96	0.0112	mt-Tr
ENSMUSG00000030109	89.13	26.95	1.73	0.0112	Slc6a12
ENSMUSG00000046844	1956.03	322.80	2.60	0.0113	Vat1l
ENSMUSG00000036095	604.84	286.22	1.08	0.0113	Dgkb
ENSMUSG00000046613	132.64	25.61	2.37	0.0113	Vwa5b2
ENSMUSG00000074274	10.16	1.96	2.38	0.0115	D930028M14Rik
ENSMUSG00000019997	56434.49	34710.28	0.70	0.0115	Ccn2
ENSMUSG00000033740	23.93	10.91	1.15	0.0115	St18
ENSMUSG00000024268	818.21	121.83	2.75	0.0115	Celf4

ENSMUSG00000042078	354.92	21.99	4.01	0.0115	Svop
ENSMUSG00000035200	910.77	56.67	4.01	0.0115	Chrnb4
ENSMUSG00000029322	361.70	500.46	-0.46	0.0116	Plac8
ENSMUSG00000056947	245.42	67.65	1.86	0.0116	Mab21l1
ENSMUSG00000020641	324.51	610.85	-0.91	0.0116	Rsad2
ENSMUSG00000078922	40.94	106.02	-1.37	0.0116	Tgtp1
ENSMUSG00000039264	126.28	283.62	-1.17	0.0116	Gimap3
ENSMUSG00000040264	1.40	13.79	-3.33	0.0116	Gbp2b
ENSMUSG00000062044	487.95	185.90	1.39	0.0116	Lmtk3
ENSMUSG00000028937	3152.13	1212.75	1.38	0.0117	Acot7
ENSMUSG00000021485	50.09	29.81	0.74	0.0117	Mxd3
ENSMUSG00000031285	12.88	1.32	3.26	0.0118	Dcx
ENSMUSG00000056856	75.51	21.12	1.83	0.0118	Jakmip3
ENSMUSG00000004113	383.43	31.30	3.61	0.0118	Cacna1b
ENSMUSG00000066392	32.26	5.54	2.54	0.0119	Nrxn3
ENSMUSG00000028072	4592.72	270.69	4.08	0.0120	Ntrk1
ENSMUSG00000029420	649.62	51.50	3.66	0.0120	Rimbp2
ENSMUSG00000061576	556.33	33.30	4.06	0.0120	Dpp6
ENSMUSG00000109695	161.90	222.06	-0.46	0.0121	Gm31166
ENSMUSG00000006575	1060.59	273.65	1.95	0.0121	Rundc3a
ENSMUSG00000018865	2583.90	155.54	4.05	0.0121	Sult4a1
ENSMUSG00000023473	252.24	67.81	1.90	0.0121	Celsr3
ENSMUSG00000107108	7.59	0.00	5.21	0.0121	Gm9936
ENSMUSG00000035640	1006.78	360.12	1.48	0.0121	Cbarp
ENSMUSG00000034336	563.10	31.65	4.15	0.0121	Ina
ENSMUSG00000026650	4.92	0.33	3.85	0.0122	Meig1
ENSMUSG00000034825	548.98	41.95	3.71	0.0124	Nrip3
ENSMUSG00000069227	159.44	28.98	2.46	0.0124	Gprin1
ENSMUSG00000027273	2606.73	188.28	3.79	0.0124	Snap25
ENSMUSG00000022211	536.64	188.22	1.51	0.0124	Carmil3
ENSMUSG00000051111	1613.05	126.22	3.68	0.0125	Sv2c
ENSMUSG00000009394	3689.41	429.47	3.10	0.0125	Syn2
ENSMUSG00000024675	46.75	79.53	-0.75	0.0125	Ms4a4c
ENSMUSG00000017943	318.34	28.42	3.48	0.0125	Gdap1l1
ENSMUSG00000037492	67.75	8.56	2.98	0.0126	Zmat4
ENSMUSG00000098062	0.64	4.94	-2.90	0.0126	Gm26931
ENSMUSG00000035692	344.75	478.09	-0.47	0.0126	Isg15
ENSMUSG00000009093	1.85	7.75	-2.11	0.0126	Gstt4
ENSMUSG00000027469	234.00	168.46	0.47	0.0127	Tpx2
ENSMUSG00000057455	835.32	64.78	3.69	0.0127	Rit2
ENSMUSG00000030428	563.01	196.39	1.52	0.0127	Ttyh1
ENSMUSG00000039375	35.65	10.76	1.73	0.0127	Wdr17
ENSMUSG00000005338	2332.89	966.64	1.27	0.0128	Cadm3
ENSMUSG00000040680	4.19	12.56	-1.57	0.0128	Kremen2
ENSMUSG00000032177	2245.10	2862.09	-0.35	0.0128	Pde4a
ENSMUSG00000031965	635.62	278.97	1.19	0.0129	Tbx20
ENSMUSG00000032303	1766.89	94.60	4.22	0.0131	Chrna3

ENSMUSG00000000214	6426.36	415.31	3.95	0.0131	Th
ENSMUSG00000089798	7.63	1.22	2.72	0.0131	1700028K03Rik
ENSMUSG00000029088	618.70	246.43	1.33	0.0131	Kcnip4
ENSMUSG00000021127	3545.77	4293.77	-0.28	0.0132	Zfp36l1
ENSMUSG00000098019	6.92	16.61	-1.24	0.0132	Gm2546
ENSMUSG00000045382	1177.16	713.62	0.72	0.0132	Cxcr4
ENSMUSG00000028931	1681.48	688.07	1.29	0.0132	Kcnab2
ENSMUSG00000034958	395.99	36.73	3.43	0.0133	Atcay
ENSMUSG00000118280	5.36	0.49	3.28	0.0133	Gm41804
ENSMUSG00000034730	293.21	80.23	1.87	0.0133	Adgrb1
ENSMUSG00000041907	105.75	22.73	2.21	0.0134	Gpr45
ENSMUSG00000005547	3.24	17.25	-2.38	0.0135	Cyp2a5
ENSMUSG00000047766	466.01	295.72	0.65	0.0135	Lrrc49
ENSMUSG00000072694	591.78	324.25	0.87	0.0135	1500011B03Rik
ENSMUSG00000036062	862.49	280.22	1.62	0.0135	Phf24
ENSMUSG00000029819	1353.22	343.15	1.98	0.0135	Npy
ENSMUSG00000051652	523.91	77.86	2.75	0.0135	Lrrc3
ENSMUSG00000027801	224.85	24.34	3.21	0.0136	Tm4sf4
ENSMUSG00000047013	138.48	37.23	1.89	0.0136	Fbxo41
ENSMUSG00000015484	440.63	27.12	4.02	0.0136	Fam163a
ENSMUSG00000015437	11.14	23.26	-1.04	0.0137	Gzmb
ENSMUSG00000003469	183.04	38.43	2.25	0.0137	Phyhip
ENSMUSG00000117730	14.16	27.55	-0.96	0.0137	Gm5503
ENSMUSG00000042216	498.74	61.58	3.02	0.0138	Sgsm1
ENSMUSG00000038860	927.95	376.06	1.30	0.0139	Garnl3
ENSMUSG00000055421	188.25	99.70	0.92	0.0139	Pcdh9
ENSMUSG00000039431	258.62	103.80	1.32	0.0139	Mtmr7
ENSMUSG00000036959	796.60	933.97	-0.23	0.0139	Bcorl1
ENSMUSG00000047139	2734.26	1154.01	1.24	0.0140	Cd24a
ENSMUSG00000019894	368.73	40.28	3.19	0.0140	Slc6a15
ENSMUSG00000114612	0.60	5.03	-2.99	0.0140	Gm47550
ENSMUSG00000031144	1808.08	494.25	1.87	0.0140	Syp
ENSMUSG00000033688	1.18	6.17	-2.30	0.0141	Inhca
ENSMUSG00000023328	2445.88	362.37	2.75	0.0141	Ache
ENSMUSG00000022054	1605.75	128.81	3.64	0.0142	Nefm
ENSMUSG00000027797	947.48	515.33	0.88	0.0143	Dclk1
ENSMUSG00000026825	3168.03	1646.41	0.94	0.0143	Dnm1
ENSMUSG00000022762	66.50	11.65	2.51	0.0144	Ncam2
ENSMUSG00000032773	258.96	26.52	3.28	0.0144	Chrm1
ENSMUSG00000064365	3.76	13.51	-1.86	0.0144	mt-Ts2
ENSMUSG00000004360	187.82	58.42	1.68	0.0144	9330159F19Rik
ENSMUSG00000095571	0.38	5.78	-3.72	0.0145	Ighv5-17
ENSMUSG00000085615	23.55	38.42	-0.72	0.0146	A330035P11Rik
ENSMUSG00000078853	644.80	1056.14	-0.71	0.0146	Igtp
ENSMUSG00000050967	269.46	99.63	1.43	0.0146	Creg2
ENSMUSG00000062542	572.62	60.22	3.25	0.0146	Syt9
ENSMUSG00000021765	463.69	361.08	0.36	0.0146	Fst

ENSMUSG00000061080	628.53	280.33	1.16	0.0146	Lsamp
ENSMUSG00000020897	100.73	66.70	0.60	0.0147	Aurkb
ENSMUSG00000052584	160.84	61.81	1.38	0.0147	Serp2
ENSMUSG00000000308	349.51	35.53	3.30	0.0148	Ckmt1
ENSMUSG00000042258	557.58	189.92	1.55	0.0148	Isl1
ENSMUSG00000045502	52.22	101.62	-0.96	0.0148	Hcar2
ENSMUSG00000076534	0.38	8.02	-4.22	0.0148	Igkv12-89
ENSMUSG00000021268	9294.65	5102.42	0.87	0.0148	Meg3
ENSMUSG00000034532	28.84	9.10	1.66	0.0148	Fbxo16
ENSMUSG00000021803	50.10	32.53	0.62	0.0148	Cdhr1
ENSMUSG00002076173	34.98	53.28	-0.60	0.0149	
ENSMUSG00000056215	18.89	3.43	2.48	0.0149	Lrguk
ENSMUSG00000041959	5076.28	4180.31	0.28	0.0149	S100a10
ENSMUSG00000053550	108.07	19.95	2.44	0.0149	Shisa7
ENSMUSG00000045349	62.56	27.43	1.18	0.0149	Sh2d5
ENSMUSG00000006651	2420.08	990.51	1.29	0.0150	Aplp1
ENSMUSG00000107314	5.22	0.24	3.94	0.0150	Gm20488
ENSMUSG00000110755	6.01	20.66	-1.81	0.0150	BC049987
ENSMUSG00000031145	643.89	896.90	-0.48	0.0150	Prickle3
ENSMUSG00000026180	13.51	26.96	-0.97	0.0150	Cxcr2
ENSMUSG00000046240	98.23	31.98	1.62	0.0150	Hepacam
ENSMUSG00000020333	163.13	43.94	1.89	0.0150	Acsl6
ENSMUSG00000009292	183.38	79.05	1.21	0.0151	Trpm2
ENSMUSG00000038704	14.82	2.88	2.39	0.0151	Aspdh
ENSMUSG00000087149	81.34	29.05	1.47	0.0151	Itih5l-ps
ENSMUSG00000040797	309.54	63.42	2.29	0.0151	Iqsec3
ENSMUSG00000020261	2538.01	1131.91	1.16	0.0152	Slc36a1
ENSMUSG00000102252	1591.98	642.21	1.31	0.0152	Snrpn
ENSMUSG00000040809	49.83	84.89	-0.77	0.0152	Chil3
ENSMUSG00000002064	1901.77	1676.65	0.18	0.0152	Sdf2
ENSMUSG00000083579	3.74	0.30	3.50	0.0152	Gm15538
ENSMUSG00000048216	87.71	28.48	1.62	0.0153	Gpr85
ENSMUSG00000103608	5.98	0.00	4.86	0.0153	4930442P19Rik
ENSMUSG00000030774	680.51	376.96	0.85	0.0154	Pak1
ENSMUSG00000048899	58.60	11.90	2.30	0.0154	Rimkla
ENSMUSG00000020471	785.07	921.55	-0.23	0.0154	Pold2
ENSMUSG00000068794	263.71	88.79	1.57	0.0155	Col28a1
ENSMUSG00000028778	304.79	26.58	3.52	0.0155	Hcrtr1
ENSMUSG00000015599	344.39	40.15	3.10	0.0155	Ttbk1
ENSMUSG00000035964	339.63	28.06	3.60	0.0155	Tmem59l
ENSMUSG00000118669	2040.44	902.10	1.18	0.0155	Arvcf
ENSMUSG00000074506	69.85	185.74	-1.42	0.0156	Gm10705
ENSMUSG00000044349	2303.41	276.32	3.06	0.0156	Snhg11
ENSMUSG00000043004	830.55	350.78	1.24	0.0156	Gng2
ENSMUSG00000041431	105.08	75.18	0.48	0.0157	Ccnb1
ENSMUSG00000042761	246.24	19.14	3.68	0.0157	Mrap2
ENSMUSG00000058248	160.70	47.24	1.76	0.0157	Kcnh1

ENSMUSG00000091636	81.35	3.67	4.46	0.0157	Akain1
ENSMUSG00000029778	884.57	298.48	1.57	0.0158	Adcyap1r1
ENSMUSG00000022415	3013.17	1097.21	1.46	0.0159	Syngr1
ENSMUSG00000032599	2198.84	1668.65	0.40	0.0160	Ip6k2
ENSMUSG00000085795	3399.53	4481.50	-0.40	0.0160	Zfp703
ENSMUSG00000026051	749.23	457.21	0.71	0.0160	Ecrg4
ENSMUSG00000029086	151.85	59.40	1.35	0.0161	Prom1
ENSMUSG00000047988	15.20	5.44	1.47	0.0161	4933428G20Rik
ENSMUSG00000058620	216.79	22.71	3.25	0.0161	Adra2b
ENSMUSG00000037259	249.34	90.22	1.46	0.0161	Dzank1
ENSMUSG00000046480	110.36	481.88	-2.13	0.0161	Scn4b
ENSMUSG00000054850	155.79	51.89	1.58	0.0162	Smim10l2a
ENSMUSG00000086363	34.38	4.52	2.93	0.0162	A330102I10Rik
ENSMUSG00000079283	229.03	293.13	-0.35	0.0162	2310009B15Rik
ENSMUSG00000048581	304.89	365.97	-0.27	0.0162	E130311K13Rik
ENSMUSG00000035864	3771.25	248.12	3.93	0.0162	Syt1
ENSMUSG00000083424	3.16	0.25	3.21	0.0163	Rpl35a-ps4
ENSMUSG00000028718	37.24	21.13	0.80	0.0163	Stil
ENSMUSG00000027514	238.55	332.20	-0.48	0.0163	Zbp1
ENSMUSG00000042873	314.39	101.79	1.63	0.0164	Lhfpl4
ENSMUSG00000042671	109.63	5.04	4.43	0.0164	Rgs8
ENSMUSG00000092229	9.99	3.79	1.46	0.0164	Gm5977
ENSMUSG00000042182	211.46	77.26	1.45	0.0165	Bend6
ENSMUSG00000116255	0.27	2.82	-3.36	0.0165	Gm20564
ENSMUSG00000120155	33.74	2.48	3.76	0.0165	
ENSMUSG00000028755	131.32	51.94	1.33	0.0166	Cda
ENSMUSG00000038576	147.47	66.10	1.15	0.0166	Susd4
ENSMUSG00000055761	11.43	0.79	3.82	0.0166	Nkain3
ENSMUSG00000045589	112.24	3.92	4.83	0.0167	Frrs1l
ENSMUSG00000063388	19.42	48.78	-1.33	0.0167	BC023105
ENSMUSG00000025194	49.65	8.81	2.50	0.0167	Abcc2
ENSMUSG00000026797	4795.82	2212.17	1.12	0.0167	Stxbp1
ENSMUSG00000102796	32.03	4.22	2.93	0.0168	Gm37711
ENSMUSG00000118018	24.45	1.31	4.20	0.0168	
ENSMUSG00000025892	361.85	188.74	0.94	0.0168	Gria4
ENSMUSG00000115180	4.67	11.26	-1.29	0.0168	Gm30556
ENSMUSG00000066150	2897.88	1096.70	1.40	0.0169	Slc31a1
ENSMUSG00000062591	3084.41	1342.78	1.20	0.0169	Tubb4a
ENSMUSG00000035365	23.70	12.30	0.97	0.0169	Parpbp
ENSMUSG00000026147	236.07	55.25	2.09	0.0169	Col9a1
ENSMUSG00000088208	0.16	2.79	-3.36	0.0169	Gm23751
ENSMUSG00000028626	427.31	101.85	2.07	0.0170	Col9a2
ENSMUSG00000070802	531.11	214.90	1.30	0.0170	Pnma8b
ENSMUSG00000025658	142.63	26.78	2.41	0.0170	Cnksr2
ENSMUSG00000036528	840.93	378.17	1.15	0.0171	Ppfibp2
ENSMUSG00000074794	2872.66	2210.34	0.38	0.0171	Arrdc3
ENSMUSG00000079339	19.38	40.23	-1.05	0.0172	Ifit1bl1

ENSMUSG00000021364	22.37	2.61	3.11	0.0172	Elovl2
ENSMUSG00000097509	4.44	13.58	-1.61	0.0172	B230322F03Rik
ENSMUSG00000082292	57.59	122.03	-1.08	0.0172	Gm12250
ENSMUSG00000094088	0.97	5.96	-2.72	0.0173	Ighv1-64
ENSMUSG00000000794	393.18	186.97	1.07	0.0174	Kcnn3
ENSMUSG00000027805	2117.45	876.57	1.27	0.0174	Pfn2
ENSMUSG00000054763	59.62	14.28	2.06	0.0175	Defb42
ENSMUSG00000056553	525.84	44.18	3.57	0.0175	Ptprn2
ENSMUSG00000015829	90.04	26.10	1.79	0.0175	Tnr
ENSMUSG00000045440	225.46	15.43	3.87	0.0175	Insm2
ENSMUSG00000027419	102.87	21.91	2.23	0.0175	Pcsk2
ENSMUSG00000120324	2.94	10.81	-1.84	0.0175	
ENSMUSG00000086429	1424.46	752.66	0.92	0.0176	Gt(ROSA)26Sor
ENSMUSG00000028528	323.15	36.12	3.16	0.0176	Dnajc6
ENSMUSG00000103469	179.32	113.32	0.66	0.0176	Gm9910
ENSMUSG00000034227	52.02	12.05	2.11	0.0176	Foxj1
ENSMUSG00000018395	859.10	607.78	0.50	0.0176	Kif3a
ENSMUSG00000030600	210.01	84.67	1.31	0.0177	Lrfn1
ENSMUSG00000004961	351.68	49.05	2.84	0.0177	Syt5
ENSMUSG00000022269	111.26	5.72	4.28	0.0177	Marchf11
ENSMUSG00000003872	46.86	19.80	1.23	0.0178	Lin7b
ENSMUSG00000032507	578.87	374.28	0.63	0.0178	Fbxl2
ENSMUSG00000025427	92.68	32.70	1.50	0.0178	Rnf165
ENSMUSG00000037843	431.86	35.91	3.59	0.0179	Vstm2l
ENSMUSG00000022454	36.52	12.89	1.50	0.0179	Nell2
ENSMUSG00000050908	176.14	97.73	0.85	0.0179	Tvp23a
ENSMUSG00000020436	60.75	3.08	4.29	0.0180	Gabrg2
ENSMUSG00000029219	1877.83	129.97	3.85	0.0180	Slc10a4
ENSMUSG00000024403	503.31	227.60	1.14	0.0180	Atp6v1g2
ENSMUSG00000031016	1137.51	790.02	0.53	0.0181	Wee1
ENSMUSG00000028926	878.59	510.67	0.78	0.0181	Cdk14
ENSMUSG00000006342	2519.32	1178.67	1.10	0.0181	Susd2
ENSMUSG00000019843	1474.21	1287.78	0.20	0.0181	Fyn
ENSMUSG00000073293	117.00	46.03	1.34	0.0181	Nudt10
ENSMUSG00000053414	192.79	59.61	1.69	0.0182	Hunk
ENSMUSG00000025582	77.75	34.94	1.15	0.0182	Nptx1
ENSMUSG00000005628	37.12	128.19	-1.79	0.0182	Tmod4
ENSMUSG00000109398	42.23	22.07	0.94	0.0182	Gm3854
ENSMUSG00000107295	4.67	0.63	3.03	0.0183	Gm42896
ENSMUSG00000105594	34.70	3.90	3.15	0.0183	Gm43398
ENSMUSG00000000632	149.22	31.77	2.23	0.0183	Sez6
ENSMUSG00000035246	108.50	37.54	1.53	0.0184	Pcyt1b
ENSMUSG00000041141	161.24	12.89	3.64	0.0184	Pnma8a
ENSMUSG00000117011	10.64	2.60	1.98	0.0185	E130008D07Rik
ENSMUSG00000029861	329.79	132.63	1.31	0.0185	Fam131b
ENSMUSG00000023064	8942.37	5281.16	0.76	0.0185	Sncg
ENSMUSG00000044197	1955.32	2525.12	-0.37	0.0185	Gpr146

ENSMUSG00000073433	544.98	95.39	2.51	0.0185	Arhgdig
ENSMUSG00000008489	561.45	41.08	3.77	0.0185	Elavl2
ENSMUSG00000001444	12.87	24.18	-0.91	0.0186	Tbx21
ENSMUSG00000096847	291.20	35.74	3.02	0.0186	Tmem151b
ENSMUSG00000043999	36.60	11.50	1.65	0.0186	Gpr75
ENSMUSG00000103175	0.26	3.68	-3.73	0.0187	Gm37169
ENSMUSG00000021194	747.11	84.99	3.14	0.0187	Chga
ENSMUSG00000021696	133.78	60.27	1.15	0.0188	Elovl7
ENSMUSG00000097391	143.25	84.66	0.76	0.0189	Mirg
ENSMUSG00000048600	73.77	39.01	0.92	0.0189	Gm5763
ENSMUSG00000072235	10600.71	4586.51	1.21	0.0189	Tuba1a
ENSMUSG00000031906	792.25	513.53	0.62	0.0190	Smpd3
ENSMUSG00000054423	650.31	46.69	3.80	0.0190	Cadps
ENSMUSG00000013611	11.72	1.34	3.11	0.0190	Snx31
ENSMUSG00000116560	0.64	6.51	-3.12	0.0191	Gm2808
ENSMUSG00000030077	788.83	254.54	1.63	0.0191	Chl1
ENSMUSG00000068099	30.45	2.51	3.60	0.0191	Smim45
ENSMUSG00000051920	42.96	23.66	0.86	0.0192	Rspo2
ENSMUSG00000102418	86.17	58.22	0.56	0.0193	Sh2d1b1
ENSMUSG00000020151	292.45	146.85	0.99	0.0193	Ptprr
ENSMUSG00000079363	402.69	716.17	-0.83	0.0193	Gbp4
ENSMUSG00000057982	888.04	692.53	0.36	0.0194	Zfp809
ENSMUSG00000045912	40.23	14.22	1.49	0.0195	C2cd4c
ENSMUSG00000113669	4.64	11.52	-1.27	0.0195	Gm36723
ENSMUSG00000052951	23.04	4.46	2.37	0.0195	C130021I20Rik
ENSMUSG00000092341	6027.99	7844.09	-0.38	0.0195	Malat1
ENSMUSG00000025738	779.42	308.35	1.34	0.0196	Fbxl16
ENSMUSG00000057818	24.71	6.17	2.00	0.0197	1600029O15Rik
ENSMUSG00000027525	349.49	174.95	1.00	0.0197	Phactr3
ENSMUSG00000021661	797.03	655.30	0.28	0.0197	Ankra2
ENSMUSG00000009630	3605.04	3078.27	0.23	0.0197	Ppp2cb
ENSMUSG00000019785	82.45	5.06	4.02	0.0197	Clvs2
ENSMUSG00000041144	46.74	30.01	0.65	0.0197	Dnah7b
ENSMUSG00000059586	636.51	557.21	0.19	0.0198	Nsmce2
ENSMUSG00000054978	92.87	123.71	-0.42	0.0199	Kbtbd13
ENSMUSG00000022416	10.75	30.31	-1.48	0.0199	Cacna1i
ENSMUSG00000044708	235.83	88.46	1.41	0.0199	Kcnj10
ENSMUSG00000026683	90.21	62.76	0.52	0.0200	Nuf2
ENSMUSG00000121096	1096.25	1258.62	-0.20	0.0201	
ENSMUSG00000059742	53.80	17.85	1.59	0.0201	Kcnh7
ENSMUSG00000051527	25.35	3.98	2.68	0.0202	Usp29
ENSMUSG00000045062	78.23	51.57	0.59	0.0202	Pcdhb7
ENSMUSG00000100658	48.46	69.20	-0.50	0.0202	F730311O21Rik
ENSMUSG00000090093	67.65	89.95	-0.41	0.0205	Gm14399
ENSMUSG00000066760	22.52	1.80	3.67	0.0205	Psg16
ENSMUSG00000032297	762.58	64.80	3.56	0.0206	Celf6
ENSMUSG00000038587	3611.35	1790.00	1.01	0.0206	Akap12

ENSMUSG00000005718	231.67	291.40	-0.33	0.0206	Tfap4
ENSMUSG00000027298	1190.57	544.78	1.13	0.0206	Tyro3
ENSMUSG00000085936	44.70	25.26	0.81	0.0207	2610307P16Rik
ENSMUSG00000032661	65.82	104.20	-0.65	0.0208	Oas3
ENSMUSG00000067438	235.16	16.12	3.86	0.0208	Hmx1
ENSMUSG00000032020	446.22	224.78	0.99	0.0208	Ubash3b
ENSMUSG00000106892	8.03	17.93	-1.12	0.0209	Gm42791
ENSMUSG00000026833	3658.52	1887.41	0.95	0.0209	Olfm1
ENSMUSG00000014846	8292.02	4068.62	1.03	0.0210	Tppp3
ENSMUSG00000067599	1.72	7.52	-2.00	0.0210	Klra7
ENSMUSG00002076304	1.34	5.83	-1.99	0.0210	7SK
ENSMUSG00000045328	68.65	47.12	0.55	0.0210	Cenpe
ENSMUSG00000079553	121.18	87.40	0.47	0.0211	Kifc1
ENSMUSG00000026109	886.43	462.04	0.94	0.0211	Tmeff2
ENSMUSG00000097451	2311.29	1202.95	0.94	0.0211	Rian
ENSMUSG00002076153	4.31	0.58	2.94	0.0211	7SK
ENSMUSG00000060212	82.98	22.07	1.91	0.0211	Pcnx2
ENSMUSG00000021448	20.01	4.24	2.25	0.0212	Shc3
ENSMUSG00000025227	335.41	173.01	0.95	0.0212	Mfsd13a
ENSMUSG00000040907	2366.08	867.88	1.45	0.0212	Atp1a3
ENSMUSG00000035898	342.97	268.74	0.35	0.0212	Uba6
ENSMUSG00000111837	30.74	8.10	1.93	0.0213	Gm47135
ENSMUSG00000056632	12.71	0.00	5.96	0.0213	Dsg3
ENSMUSG00000032452	117.14	50.74	1.21	0.0213	Clstn2
ENSMUSG00000040405	0.00	4.41	-4.73	0.0214	Havcr1
ENSMUSG00000040722	1617.96	761.04	1.09	0.0214	Scamp5
ENSMUSG00000064357	4069.13	11781.69	-1.53	0.0216	mt-Atp6
ENSMUSG00000120765	7.73	20.61	-1.43	0.0216	
ENSMUSG00000076618	0.22	2.82	-3.37	0.0216	Ighj4
ENSMUSG00000098149	0.85	4.45	-2.36	0.0217	Gapdh-ps14
ENSMUSG00000033031	84.45	56.82	0.58	0.0217	Cip2a
ENSMUSG00000055067	455.49	317.07	0.52	0.0217	Smyd3
ENSMUSG00000044042	502.65	283.32	0.83	0.0218	Fmn1
ENSMUSG00000022629	919.41	260.14	1.82	0.0218	Kif21a
ENSMUSG00000025909	19.19	6.94	1.49	0.0218	Sntg1
ENSMUSG00000033882	3.00	0.25	3.13	0.0218	Rbm46
ENSMUSG00000042589	164.10	66.79	1.30	0.0218	Cux2
ENSMUSG00000086171	15.45	0.98	3.91	0.0219	Pcsk2os1
ENSMUSG00000072966	504.91	275.76	0.87	0.0219	Gprasp2
ENSMUSG00000003410	462.20	53.65	3.11	0.0219	Elavl3
ENSMUSG00000060621	12.17	1.53	2.95	0.0219	Nkpd1
ENSMUSG00000032940	63.52	17.77	1.83	0.0220	Rbm11
ENSMUSG00000000861	23.71	39.59	-0.75	0.0221	Bcl11a
ENSMUSG00000070298	4.86	0.79	2.55	0.0221	Trcg1
ENSMUSG00000032564	9.17	1.15	3.01	0.0221	Cpne4
ENSMUSG00000024256	14.12	1.42	3.33	0.0221	Adcyap1
ENSMUSG00000108255	30.94	6.18	2.33	0.0222	Gm16499

ENSMUSG00000062309	38.90	11.04	1.82	0.0222	Rpp25
ENSMUSG00000040093	528.29	677.46	-0.36	0.0222	Bmf
ENSMUSG00000054409	20.89	2.75	2.92	0.0222	Tmem74
ENSMUSG00000030393	94.42	66.89	0.49	0.0222	Zik1
ENSMUSG00000089417	10.15	21.01	-1.06	0.0223	Gm22009
ENSMUSG00000104706	0.16	2.68	-3.32	0.0223	Gm43421
ENSMUSG00000017737	231.72	314.10	-0.44	0.0223	Mmp9
ENSMUSG00000021670	749.35	396.31	0.92	0.0223	Hmgcr
ENSMUSG00000033768	721.92	271.93	1.41	0.0224	Nrxn2
ENSMUSG00000040875	559.75	298.88	0.90	0.0224	Osbpl10
ENSMUSG00000094910	347.57	133.02	1.38	0.0225	D430019H16Rik
ENSMUSG00000027560	63.93	19.27	1.73	0.0228	Dok5
ENSMUSG00000003411	1265.11	143.52	3.14	0.0228	Rab3b
ENSMUSG00000027993	1095.14	754.49	0.54	0.0228	Trim2
ENSMUSG00000045179	9.75	0.49	4.17	0.0228	Sox3
ENSMUSG00000085133	109.61	53.02	1.05	0.0228	B930095G15Rik
ENSMUSG00000024558	94.09	48.50	0.95	0.0228	Mapk4
ENSMUSG00000036006	115.07	197.19	-0.78	0.0229	Ripor2
ENSMUSG00000068923	4095.31	1938.95	1.08	0.0229	Syt11
ENSMUSG00000044647	41.72	9.19	2.18	0.0229	Csrnp3
ENSMUSG00000034226	93.18	43.24	1.10	0.0229	Rhov
ENSMUSG00000038486	1419.55	562.18	1.34	0.0229	Sv2a
ENSMUSG00000091694	16.21	37.95	-1.23	0.0229	Apol11b
ENSMUSG00000028488	197.02	24.70	3.00	0.0230	Sh3gl2
ENSMUSG00000041020	584.75	342.81	0.77	0.0231	Map7d2
ENSMUSG00000043811	55.61	21.97	1.34	0.0231	Rtn4r
ENSMUSG00000085411	0.66	3.89	-2.56	0.0231	Gm14319
ENSMUSG00000051359	2127.51	1033.95	1.04	0.0231	Ncald
ENSMUSG00000048756	2457.04	1971.11	0.32	0.0231	Foxo3
ENSMUSG00000057802	3.04	9.19	-1.57	0.0232	Gm10030
ENSMUSG00000042428	552.91	295.26	0.91	0.0232	Mgat3
ENSMUSG00000015697	1123.28	998.18	0.17	0.0233	Setdb1
ENSMUSG00000054934	39.12	8.88	2.14	0.0233	Kcnmb4
ENSMUSG00000035984	49.81	29.17	0.75	0.0233	Nme5
ENSMUSG00000113167	1.94	7.96	-1.96	0.0234	Gm48482
ENSMUSG00000050711	787.93	91.12	3.11	0.0234	Scg2
ENSMUSG00000086527	4.00	11.65	-1.54	0.0235	Gm15856
ENSMUSG00000110601	4.56	11.97	-1.41	0.0235	Gm45834
ENSMUSG00000068551	1377.57	1608.48	-0.22	0.0235	Zfp467
ENSMUSG00000056128	14.30	25.37	-0.83	0.0235	Gm9991
ENSMUSG00000045648	81.64	3.27	4.64	0.0235	Vwc2l
ENSMUSG00000046031	59.90	98.21	-0.72	0.0236	Calhm6
ENSMUSG00000114041	11.10	4.49	1.30	0.0236	Gm9269
ENSMUSG00000032454	4.48	0.88	2.43	0.0236	Rbp2
ENSMUSG00000057363	946.41	803.04	0.24	0.0237	Uxs1
ENSMUSG00000113178	11.23	45.52	-2.02	0.0237	Mylf-ps
ENSMUSG00000037347	264.35	219.46	0.27	0.0237	Chst7

ENSMUSG00000020096	17.54	7.47	1.24	0.0237	Tbata
ENSMUSG00000045288	17.07	4.72	1.88	0.0237	Ush1g
ENSMUSG00000033419	655.46	53.83	3.61	0.0238	Snap91
ENSMUSG00000051242	165.55	103.23	0.68	0.0238	Pcdhb9
ENSMUSG00000086745	13.70	3.55	1.94	0.0238	Tspan2os
ENSMUSG00000078875	1.97	9.64	-2.22	0.0238	Gm14419
ENSMUSG00000049336	103.68	34.30	1.59	0.0239	Tenm2
ENSMUSG00000041817	70.49	19.29	1.87	0.0239	Fam169a
ENSMUSG00000021215	6289.75	4803.68	0.39	0.0239	Net1
ENSMUSG00000053111	10.30	1.17	3.17	0.0240	Fank1
ENSMUSG00000044067	85.12	4.59	4.20	0.0240	Gpr22
ENSMUSG00000068264	720.97	861.75	-0.26	0.0240	Ap5s1
ENSMUSG00000106947	4.66	0.74	2.54	0.0240	1700025A08Rik
ENSMUSG00000029291	1630.16	747.85	1.12	0.0241	Rufy3
ENSMUSG00000094091	2.99	0.25	3.16	0.0242	Ankrd40cl
ENSMUSG00000074595	4.75	0.80	2.52	0.0242	Wfdc6a
ENSMUSG00000073940	3372.18	9694.83	-1.52	0.0242	Hbb-bt
ENSMUSG00000040855	382.50	201.79	0.92	0.0242	Reps2
ENSMUSG00000114278	181.31	24.38	2.89	0.0243	Gm49027
ENSMUSG00000039154	136.24	60.35	1.17	0.0243	Shd
ENSMUSG00000047643	40.83	26.98	0.61	0.0244	Gm5454
ENSMUSG00000028546	337.36	27.93	3.59	0.0244	Elavl4
ENSMUSG00000030302	535.12	249.94	1.10	0.0245	Atp2b2
ENSMUSG00000045689	46.64	26.24	0.81	0.0246	Pcdhb4
ENSMUSG00000062760	71.56	15.84	2.17	0.0246	Shisal1
ENSMUSG00000033618	102.64	70.97	0.52	0.0248	Map3k13
ENSMUSG00000097387	0.64	3.77	-2.54	0.0248	4930563E18Rik
ENSMUSG00000053038	42.71	27.21	0.64	0.0252	Gm6180
ENSMUSG00000063600	9.18	2.05	2.11	0.0253	Egfem1
ENSMUSG00000078920	466.93	611.07	-0.39	0.0253	Ifi47
ENSMUSG00000059361	166.81	17.29	3.27	0.0254	Nrsn2
ENSMUSG00000103901	44.24	28.70	0.64	0.0255	Gm37499
ENSMUSG00000109973	18.46	7.96	1.18	0.0255	Gm45397
ENSMUSG00000056073	55.52	19.41	1.53	0.0255	Grik2
ENSMUSG00000034007	467.54	365.39	0.36	0.0255	Scaper
ENSMUSG00000007908	42.47	6.90	2.61	0.0255	Hmgcll1
ENSMUSG00000071113	4.96	0.82	2.58	0.0256	Mboat4
ENSMUSG00000026435	539.02	357.63	0.59	0.0257	Slc45a3
ENSMUSG00000028789	912.34	444.86	1.04	0.0258	Azin2
ENSMUSG00000026959	171.58	16.00	3.42	0.0258	Grin1
ENSMUSG00000087030	4.31	13.41	-1.67	0.0258	Gm16143
ENSMUSG00000028252	691.81	569.04	0.28	0.0258	Ccnc
ENSMUSG00000029384	4.36	12.23	-1.50	0.0259	2010109A12Rik
ENSMUSG00000022034	34.27	20.27	0.78	0.0259	Esco2
ENSMUSG00000027239	601.44	425.10	0.50	0.0261	Mdk
ENSMUSG00000032624	419.95	345.68	0.28	0.0262	Eml4
ENSMUSG00000022055	1245.26	129.77	3.26	0.0262	Nefl

ENSMUSG00000038555	1327.15	621.62	1.09	0.0263	Reep2
ENSMUSG00000041263	758.33	503.16	0.59	0.0264	Rusc1
ENSMUSG00000089812	161.56	106.68	0.59	0.0265	Gm15867
ENSMUSG00000042745	2447.22	1853.06	0.40	0.0265	Id1
ENSMUSG00000090100	659.67	416.38	0.66	0.0265	Ttbk2
ENSMUSG00000002190	131.33	14.16	3.21	0.0265	Clgn
ENSMUSG00000039347	1525.32	734.72	1.05	0.0266	Atp6v0e2
ENSMUSG00000109598	17.06	4.69	1.88	0.0267	Gm36356
ENSMUSG00000046006	17.07	30.43	-0.81	0.0268	Gapt
ENSMUSG00000038530	3335.99	1862.22	0.84	0.0269	Rgs4
ENSMUSG00000040009	364.53	157.77	1.21	0.0269	Gnaz
ENSMUSG00000053137	530.40	232.52	1.19	0.0270	Mapk11
ENSMUSG00000015656	45816.46	38235.98	0.26	0.0271	Hspa8
ENSMUSG00000098934	0.00	2.34	-3.81	0.0272	Gvin-ps4
ENSMUSG00000045532	51.19	8.40	2.60	0.0272	C1ql1
ENSMUSG00000046687	480.52	292.23	0.72	0.0273	Gm5424
ENSMUSG00000048644	651.74	278.54	1.23	0.0273	Ctxn1
ENSMUSG00000023995	3.78	10.88	-1.55	0.0274	Tspo2
ENSMUSG00000034855	65.32	114.57	-0.82	0.0274	Cxcl10
ENSMUSG00000033578	578.21	272.18	1.09	0.0274	Tmem35a
ENSMUSG00000022102	357.31	449.28	-0.33	0.0274	Dok2
ENSMUSG00000056458	56.45	31.19	0.85	0.0275	Mok
ENSMUSG00000070803	44.52	62.53	-0.49	0.0275	Cited4
ENSMUSG00000049404	614.61	398.95	0.62	0.0276	Rarres1
ENSMUSG00000042489	65.46	40.15	0.71	0.0276	Clspn
ENSMUSG00000037169	95.36	27.23	1.81	0.0276	Mycn
ENSMUSG00000020701	346.99	125.16	1.47	0.0276	Tmem132e
ENSMUSG00000028035	4873.58	3638.55	0.42	0.0276	Dnajb4
ENSMUSG00000051316	300.54	351.46	-0.22	0.0276	Taf7
ENSMUSG00000038077	613.25	278.01	1.14	0.0276	Kcna6
ENSMUSG00000045010	6.14	0.90	2.84	0.0276	Gm4779
ENSMUSG00000025959	1966.52	1408.84	0.48	0.0277	Klf7
ENSMUSG00000025498	793.84	1074.24	-0.44	0.0277	Irf7
ENSMUSG00000056854	2.33	0.00	3.52	0.0277	Pou3f4
ENSMUSG00000041538	79.77	161.55	-1.02	0.0277	H2-Ob
ENSMUSG00000047428	15.92	5.54	1.55	0.0280	Dlk2
ENSMUSG00000074899	106.07	191.47	-0.85	0.0280	Sptbn5
ENSMUSG00000031398	519.91	296.13	0.81	0.0281	Plxna3
ENSMUSG00000067231	10.22	0.00	5.64	0.0281	Cyp2c65
ENSMUSG00000111180	0.48	5.16	-3.46	0.0282	Gm39348
ENSMUSG00000068009	0.22	2.92	-3.41	0.0282	Bpifb6
ENSMUSG00000079523	6973.66	4879.72	0.52	0.0282	Tmsb10
ENSMUSG00000086564	12.19	22.96	-0.90	0.0285	Cd101
ENSMUSG00000074102	1398.13	1234.13	0.18	0.0285	Rbm15b
ENSMUSG00000041592	375.82	304.10	0.30	0.0285	Sdk2
ENSMUSG00000045394	12.39	4.09	1.59	0.0285	Epcam
ENSMUSG00000037605	44.52	14.34	1.64	0.0286	Adgrl3

ENSMUSG00000041670	29.28	9.97	1.54	0.0286	Rims1
ENSMUSG00000029161	616.34	361.60	0.77	0.0286	Cgref1
ENSMUSG00000032186	1341.79	667.48	1.01	0.0287	Tmod2
ENSMUSG00000120351	7.00	15.37	-1.10	0.0287	
ENSMUSG00000024330	1442.61	753.41	0.94	0.0287	Col11a2
ENSMUSG00000027457	284.05	32.26	3.14	0.0288	Snph
ENSMUSG00000074171	58.28	92.19	-0.66	0.0289	Gm6658
ENSMUSG00000069309	0.27	2.51	-3.20	0.0290	H2ac22
ENSMUSG00000022756	137.56	55.89	1.30	0.0291	Slc7a4
ENSMUSG00000041707	150.15	121.65	0.30	0.0291	Tmem273
ENSMUSG00000029414	38.45	21.83	0.82	0.0294	Kntc1
ENSMUSG00000106970	15.67	0.49	4.86	0.0294	Gm43377
ENSMUSG00000031840	2210.22	1189.79	0.89	0.0295	Rab3a
ENSMUSG00000024979	8.57	22.00	-1.38	0.0296	Tectb
ENSMUSG00000075023	9.60	20.92	-1.13	0.0296	Accsl
ENSMUSG00000022351	1183.07	534.47	1.15	0.0297	Sqle
ENSMUSG00000034098	308.88	18.87	4.03	0.0297	Fstl5
ENSMUSG00000086148	11.35	4.79	1.21	0.0297	Gm15271
ENSMUSG00000020312	1522.83	946.77	0.69	0.0298	Shc2
ENSMUSG00000036948	1826.35	1267.66	0.53	0.0298	Trappc14
ENSMUSG00000047153	963.58	1105.97	-0.20	0.0299	Khnyn
ENSMUSG00000052560	627.39	530.93	0.24	0.0299	Cpne8
ENSMUSG00000081219	52.20	81.25	-0.63	0.0299	Bambi-ps1
ENSMUSG00000120977	8.89	3.52	1.34	0.0300	
ENSMUSG00000030483	149.03	477.67	-1.68	0.0301	Cyp2b10
ENSMUSG00000103436	3.82	9.10	-1.23	0.0301	Gm36995
ENSMUSG00000041608	392.62	43.87	3.16	0.0301	Entpd3
ENSMUSG00000024968	408.86	541.81	-0.41	0.0302	Rcor2
ENSMUSG00000046093	530.09	102.43	2.37	0.0303	Hpcal4
ENSMUSG00000022962	1262.15	1408.55	-0.16	0.0303	Gart
ENSMUSG00000111977	153.56	68.67	1.16	0.0304	Gm47163
ENSMUSG00000103189	10.58	0.65	4.18	0.0304	Gm37092
ENSMUSG00000115681	2.95	13.67	-2.16	0.0305	Gm2682
ENSMUSG00000086727	15.88	48.56	-1.62	0.0305	4931428L18Rik
ENSMUSG00000022893	6349.53	4452.27	0.51	0.0305	Adamts1
ENSMUSG00000043388	348.88	34.98	3.32	0.0306	Tmem130
ENSMUSG00000066705	2990.90	1489.14	1.01	0.0307	Fxyd6
ENSMUSG00000058503	568.96	496.96	0.20	0.0308	Fam133b
ENSMUSG00000042303	888.38	705.02	0.33	0.0308	Sgsm3
ENSMUSG00000081251	5.78	1.67	1.79	0.0308	Gm12164
ENSMUSG00000000903	4.70	11.66	-1.31	0.0309	Vpreb3
ENSMUSG00000101067	11.49	27.62	-1.27	0.0310	Gm29007
ENSMUSG00000061702	18.33	30.91	-0.75	0.0310	Tmem91
ENSMUSG00000058046	34.86	22.79	0.61	0.0311	4933430I17Rik
ENSMUSG00000084866	12.20	22.10	-0.82	0.0311	A930006K02Rik
ENSMUSG00000071052	494.48	631.65	-0.35	0.0311	Rpl7a-ps5
ENSMUSG00000026011	5.88	22.14	-1.89	0.0312	Ctla4

ENSMUSG00000037321	1068.78	1473.74	-0.46	0.0312	Tap1
ENSMUSG00000027584	108.49	10.51	3.36	0.0313	Oprl1
ENSMUSG00000024986	284.72	362.43	-0.35	0.0313	Hhex
ENSMUSG00000040389	769.01	518.74	0.57	0.0314	Wdr47
ENSMUSG00000109599	8.75	26.20	-1.58	0.0314	Gm31812
ENSMUSG00000048996	5.87	1.52	2.07	0.0314	Olfr1366
ENSMUSG00000037600	0.00	2.00	-3.59	0.0314	Kdf1
ENSMUSG00000060461	2.73	0.30	3.04	0.0314	Dppa5a
ENSMUSG00000096715	1.19	4.41	-1.98	0.0315	Igkv3-4
ENSMUSG00000007659	3788.51	2978.18	0.35	0.0315	Bcl2l1
ENSMUSG00000023070	8.76	1.99	2.16	0.0315	Rgn
ENSMUSG00000014786	213.04	153.44	0.47	0.0315	Slc9a5
ENSMUSG00000059554	407.61	283.91	0.52	0.0315	Ccdc28a
ENSMUSG00000041986	76.38	18.82	2.03	0.0316	Elmod1
ENSMUSG00000034460	199.43	152.01	0.40	0.0316	Six4
ENSMUSG00000053835	216.29	330.58	-0.61	0.0316	H2-T24
ENSMUSG00000028865	20.00	49.53	-1.31	0.0316	Cd164l2
ENSMUSG00000083332	2.83	0.25	3.07	0.0316	Gm7599
ENSMUSG00000025964	514.00	260.15	0.98	0.0317	Adam23
ENSMUSG00000034926	1811.76	744.54	1.28	0.0317	Dhcr24
ENSMUSG00000054727	2.77	0.25	3.05	0.0317	1700013H16Rik
ENSMUSG00000058441	137.94	33.57	2.04	0.0318	Panx2
ENSMUSG00000032826	3093.91	1819.26	0.77	0.0318	Ank2
ENSMUSG00000054457	30.91	5.14	2.57	0.0319	9430021M05Rik
ENSMUSG00000074766	670.33	535.71	0.32	0.0319	Ism1
ENSMUSG00000019947	3397.16	2573.02	0.40	0.0319	Arid5b
ENSMUSG00000029162	556.90	715.23	-0.36	0.0320	Khk
ENSMUSG00000024873	70.14	33.75	1.05	0.0321	Cnih2
ENSMUSG00000041180	36.53	21.27	0.77	0.0321	Hectd2
ENSMUSG00000026841	36.40	4.92	2.87	0.0322	Fibcd1
ENSMUSG00000030865	217.11	280.68	-0.37	0.0322	Chp2
ENSMUSG00000051977	137.31	219.10	-0.67	0.0322	Prdm9
ENSMUSG00000050511	7.29	1.14	2.69	0.0323	Oprd1
ENSMUSG00000032254	110.35	79.90	0.47	0.0323	Kif23
ENSMUSG00000078896	9.35	2.66	1.80	0.0324	Zfp965
ENSMUSG00000020654	1894.37	2218.53	-0.23	0.0324	Adcy3
ENSMUSG00000051855	298.60	128.41	1.22	0.0324	Mest
ENSMUSG00000032231	18624.55	16176.44	0.20	0.0325	Anxa2
ENSMUSG00000049191	318.05	244.91	0.37	0.0325	Rtl5
ENSMUSG00000002459	38.58	8.35	2.20	0.0326	Rgs20
ENSMUSG00000030757	19.43	5.01	1.96	0.0326	Zkscan2
ENSMUSG00000110279	29.78	63.64	-1.10	0.0327	Gm45552
ENSMUSG00000109095	4.79	0.33	3.83	0.0327	Gm44799
ENSMUSG00000020914	374.76	258.86	0.53	0.0327	Top2a
ENSMUSG00000025533	1688.79	917.58	0.88	0.0328	Asl
ENSMUSG00000004668	28.30	15.22	0.88	0.0329	Abca13
ENSMUSG00000032625	399.10	229.14	0.80	0.0329	Thsd7a

ENSMUSG00000032549	4317.09	1938.81	1.15	0.0329	Rab6b
ENSMUSG00000031204	8.40	19.67	-1.21	0.0330	Asb12
ENSMUSG00000025478	172.95	62.48	1.47	0.0330	Dpysl4
ENSMUSG00000054083	35.24	55.64	-0.66	0.0330	Capn12
ENSMUSG00000031255	131.46	170.74	-0.37	0.0331	Sytl4
ENSMUSG00000015709	214.09	106.41	1.01	0.0331	Arnt2
ENSMUSG00000020230	1432.22	933.53	0.62	0.0331	Prmt2
ENSMUSG00000041556	1122.19	464.32	1.27	0.0331	Fbxo2
ENSMUSG00000040640	116.12	9.90	3.55	0.0331	Erc2
ENSMUSG00000019876	187.20	120.85	0.63	0.0331	Pkib
ENSMUSG00000040591	857.07	559.50	0.61	0.0331	1110051M20Rik
ENSMUSG00000027376	10.03	3.35	1.66	0.0332	Prom2
ENSMUSG00000113654	2.38	0.00	3.52	0.0332	Gm30893
ENSMUSG00000116165	132.77	51.06	1.38	0.0333	Pdxp
ENSMUSG00000118456	26.24	41.19	-0.66	0.0334	Gm41381
ENSMUSG00000031099	120.80	64.14	0.91	0.0334	Smarca1
ENSMUSG00000096596	2.98	8.00	-1.40	0.0334	Gm10591
ENSMUSG00000054720	1883.36	1306.32	0.53	0.0334	Lrrc8c
ENSMUSG00000009731	237.84	128.12	0.89	0.0335	Kcnd1
ENSMUSG00000021508	406.14	683.42	-0.75	0.0335	Cxcl14
ENSMUSG00000018362	981.98	818.95	0.26	0.0335	Kpna2
ENSMUSG00000028351	150.36	68.17	1.14	0.0336	Brinp1
ENSMUSG00000108421	4.03	0.00	4.29	0.0337	Gm44718
ENSMUSG00000098097	8.94	0.25	4.73	0.0337	6530403H02Rik
ENSMUSG00000028459	946.40	544.68	0.80	0.0338	Cd72
ENSMUSG00000019878	571.50	475.68	0.27	0.0338	Hsf2
ENSMUSG00000036192	62.60	20.27	1.63	0.0338	Rorb
ENSMUSG00000087651	425.07	284.52	0.58	0.0338	1500009L16Rik
ENSMUSG00000077711	12.85	5.77	1.14	0.0338	AF357399
ENSMUSG00000025058	53.33	72.86	-0.45	0.0339	Tasl
ENSMUSG00000021991	380.97	195.33	0.96	0.0339	Cacna2d3
ENSMUSG00000074872	26.66	3.58	2.90	0.0339	Ctxn2
ENSMUSG00000095127	0.60	4.93	-2.99	0.0340	Ighv1-82
ENSMUSG00000071553	9.59	2.08	2.17	0.0340	Cpa2
ENSMUSG00000018476	2522.82	3014.26	-0.26	0.0340	Kdm6b
ENSMUSG00000107826	5.23	0.30	3.95	0.0340	Gm44242
ENSMUSG00000041658	451.57	230.87	0.97	0.0341	Rragb
ENSMUSG00000098483	6.40	1.79	1.76	0.0341	Mir7078
ENSMUSG00000021365	2238.43	1771.71	0.34	0.0341	Nedd9
ENSMUSG00000021675	70.62	19.90	1.83	0.0341	F2rl2
ENSMUSG00000112657	9.36	3.29	1.57	0.0342	BC106175
ENSMUSG00000040570	92.84	37.59	1.31	0.0343	Rundc3b
ENSMUSG00000104362	17.43	0.98	4.10	0.0343	Gm37928
ENSMUSG00000068227	81.85	127.91	-0.64	0.0343	Il2rb
ENSMUSG00000073079	168.43	109.12	0.62	0.0344	Srp54a
ENSMUSG00000097311	5.89	0.57	3.41	0.0344	Gm26871
ENSMUSG00000029130	27.89	16.51	0.76	0.0344	Rnf32

ENSMUSG00000056418	216.64	268.08	-0.31	0.0344	BC043934
ENSMUSG00000082194	0.00	2.91	-4.13	0.0344	Gm12444
ENSMUSG00000032570	1524.27	1232.03	0.31	0.0346	Atp2c1
ENSMUSG00000019124	1898.40	1100.45	0.79	0.0346	Scrn1
ENSMUSG00000036578	582.13	289.82	1.01	0.0346	Fxyd7
ENSMUSG00000057465	10.45	3.35	1.64	0.0346	Saa2
ENSMUSG00000030518	24.86	3.93	2.65	0.0346	Fam189a1
ENSMUSG00000074217	32.68	21.58	0.59	0.0346	Misp3
ENSMUSG00000025776	716.03	484.06	0.56	0.0346	Crispld1
ENSMUSG00000038128	85.96	36.98	1.21	0.0346	Camk4
ENSMUSG00000024424	362.93	233.69	0.63	0.0347	Ttc39c
ENSMUSG00000064125	66.72	24.57	1.44	0.0349	Prr36
ENSMUSG00000038421	23.93	50.42	-1.06	0.0351	Fcrla
ENSMUSG00000095583	0.32	3.92	-3.21	0.0352	Ighv14-2
ENSMUSG00000031937	96.37	42.83	1.17	0.0353	Vstm5
ENSMUSG00000022704	543.15	482.05	0.18	0.0353	Qtrt2
ENSMUSG00000106641	0.42	3.17	-2.84	0.0353	Gm43534
ENSMUSG00000110588	45.34	27.18	0.74	0.0353	Gm45774
ENSMUSG00000058388	950.17	749.43	0.34	0.0353	Phtf1
ENSMUSG00000071671	4.44	0.85	2.44	0.0354	Gm10343
ENSMUSG00000073102	17.38	5.29	1.69	0.0354	Drc1
ENSMUSG00000039740	1847.84	1004.70	0.88	0.0355	Alg2
ENSMUSG00000034768	28.81	65.68	-1.18	0.0355	Asb16
ENSMUSG00000028277	2875.37	2436.29	0.24	0.0356	Ube2j1
ENSMUSG00000031712	185.81	222.48	-0.26	0.0356	Il15
ENSMUSG00000100150	1.56	6.65	-1.99	0.0357	Gm19585
ENSMUSG00000040086	72.84	139.13	-0.93	0.0357	Tnni3k
ENSMUSG00000022860	29.65	2.94	3.35	0.0357	Chodl
ENSMUSG00000090330	169.05	217.95	-0.37	0.0358	9130221H12Rik
ENSMUSG00000097545	78.90	6.34	3.64	0.0359	Mir124a-1hg
ENSMUSG00000044201	25.88	14.16	0.88	0.0359	Cdc25c
ENSMUSG00000044820	3.93	0.65	2.80	0.0361	AY074887
ENSMUSG00000120176	32.30	54.51	-0.76	0.0362	
ENSMUSG00000104713	123.87	220.43	-0.83	0.0362	Gbp6
ENSMUSG00000035580	27.85	7.44	1.90	0.0362	Kcnh8
ENSMUSG00000070337	20.48	6.16	1.72	0.0362	Gpr179
ENSMUSG00000029359	707.25	350.07	1.01	0.0362	Tesc
ENSMUSG00000035547	2604.12	1455.76	0.84	0.0364	Capn5
ENSMUSG00000049252	28.43	8.37	1.76	0.0364	Lrp1b
ENSMUSG00000044176	23.51	1.77	3.71	0.0364	Spink10
ENSMUSG00000021288	10171.98	5894.13	0.79	0.0364	Klc1
ENSMUSG00000064360	653.08	1444.45	-1.15	0.0364	mt-Nd3
ENSMUSG00000103809	0.00	2.68	-4.03	0.0364	Gm37061
ENSMUSG00000086109	34.10	20.63	0.74	0.0365	Gm13391
ENSMUSG00000028785	63.28	17.57	1.85	0.0366	Hpca
ENSMUSG00000056596	1867.13	922.25	1.02	0.0366	Trnp1
ENSMUSG00000021071	43.59	11.48	1.92	0.0366	Trim9

ENSMUSG00000028730	6.86	1.82	1.98	0.0367	Cfap57
ENSMUSG00000019146	116.09	5.17	4.48	0.0367	Cacng2
ENSMUSG00000037892	172.95	221.95	-0.35	0.0367	Pcdh18
ENSMUSG00000026587	290.45	29.12	3.32	0.0367	Astn1
ENSMUSG00000020181	195.28	140.47	0.48	0.0368	Nav3
ENSMUSG00000020542	278.33	203.60	0.45	0.0368	Myocd
ENSMUSG00000036198	45.83	22.89	0.98	0.0368	Arhgap36
ENSMUSG00000033717	909.93	399.55	1.19	0.0368	Adra2a
ENSMUSG00000045009	40.75	15.30	1.40	0.0368	Prrt3
ENSMUSG00000094006	0.00	4.40	-4.73	0.0369	Igkv4-59
ENSMUSG00000016541	3827.62	2807.71	0.45	0.0370	Atxn10
ENSMUSG00000044667	53.29	16.07	1.73	0.0371	Plppr4
ENSMUSG00000010362	840.65	994.39	-0.24	0.0371	Rdm1
ENSMUSG00000039911	4744.89	3727.96	0.35	0.0373	Spsb1
ENSMUSG00000036834	6.09	1.15	2.46	0.0373	Plch1
ENSMUSG00000116604	12.54	21.92	-0.81	0.0373	Gm49745
ENSMUSG00000052751	1038.45	1225.61	-0.24	0.0373	Repin1
ENSMUSG00000068874	2901.07	3578.36	-0.30	0.0373	Selenbp1
ENSMUSG00000078606	44.88	77.25	-0.77	0.0373	Gvin2
ENSMUSG00000034987	48.10	73.57	-0.62	0.0373	Hrh2
ENSMUSG00000039989	1637.33	2093.71	-0.36	0.0374	Cbx4
ENSMUSG00000020524	422.75	197.41	1.10	0.0374	Gria1
ENSMUSG00000002055	68.62	46.50	0.55	0.0374	Spag5
ENSMUSG00000028712	0.00	2.02	-3.60	0.0375	Cyp4a31
ENSMUSG00000060548	13.67	26.86	-0.95	0.0375	Tnfrsf19
ENSMUSG00000036466	71.97	27.57	1.38	0.0376	Megf11
ENSMUSG00000049420	60.51	13.56	2.16	0.0376	Tmem200a
ENSMUSG00000036291	525.75	654.84	-0.31	0.0378	Ap5m1
ENSMUSG00000039488	8.73	0.33	4.69	0.0380	Cntn5
ENSMUSG00000018398	4612.96	3956.62	0.22	0.0380	Septin8
ENSMUSG00000068617	23.60	3.98	2.56	0.0382	Efcab1
ENSMUSG00000063887	16.00	5.53	1.56	0.0383	Nlgn1
ENSMUSG00000033161	23617.67	14544.99	0.70	0.0383	Atp1a1
ENSMUSG00000097362	3.06	0.33	3.16	0.0383	Gm26544
ENSMUSG00000028417	45.27	27.96	0.70	0.0384	Tal2
ENSMUSG00000034639	141.05	177.67	-0.33	0.0384	Setmar
ENSMUSG00000036158	772.86	919.45	-0.25	0.0384	Prickle1
ENSMUSG00000113848	68.45	41.07	0.72	0.0384	Gm16419
ENSMUSG00000104508	2.68	7.91	-1.47	0.0385	Gm37735
ENSMUSG00000037466	81.68	58.56	0.48	0.0385	Tedc1
ENSMUSG00000061298	8.21	0.88	3.26	0.0385	Agbl4
ENSMUSG00000043931	11.30	26.74	-1.24	0.0387	Gimap7
ENSMUSG00000018012	470.34	335.23	0.49	0.0387	Rac3
ENSMUSG00000040537	384.34	206.39	0.90	0.0387	Adam22
ENSMUSG00000059873	5.49	1.56	1.75	0.0387	Olfr1029
ENSMUSG00000035615	299.97	43.86	2.77	0.0388	Frmpd1
ENSMUSG00000046694	971.24	598.90	0.70	0.0388	Tent5b

ENSMUSG00000041471	141.57	189.98	-0.42	0.0388	Shld2
ENSMUSG00000068452	10.73	21.96	-0.99	0.0389	Duox2
ENSMUSG00000103767	3.06	8.55	-1.51	0.0390	Gm37858
ENSMUSG00000104649	5.66	1.48	2.02	0.0392	Gm43712
ENSMUSG00000024642	635.03	463.62	0.45	0.0393	Tle4
ENSMUSG00000029915	234.03	185.99	0.33	0.0393	Clec5a
ENSMUSG00000053846	56.67	24.67	1.20	0.0394	Lipg
ENSMUSG00000037375	169.31	123.20	0.45	0.0394	Hhat
ENSMUSG00000017639	643.08	279.13	1.20	0.0394	Rab11fip4
ENSMUSG00000030770	10615.81	6828.53	0.64	0.0394	Parva
ENSMUSG00000039661	384.96	122.59	1.65	0.0394	Dusp26
ENSMUSG00000090698	119.92	207.78	-0.79	0.0394	Apold1
ENSMUSG00000047085	466.15	214.69	1.12	0.0394	Lrrc4b
ENSMUSG00000073295	186.41	86.85	1.10	0.0395	Nudt11
ENSMUSG00000055177	2.34	0.00	3.53	0.0395	Cstl1
ENSMUSG00000000296	450.79	310.06	0.54	0.0396	Tpd52l1
ENSMUSG00000034282	297.14	147.65	1.01	0.0396	Evpl
ENSMUSG00000085928	0.00	3.43	-4.36	0.0397	4933427I22Rik
ENSMUSG00000036646	3133.41	2798.73	0.16	0.0397	Man1b1
ENSMUSG00000120820	2.03	7.58	-1.97	0.0398	
ENSMUSG00000040746	2984.58	3291.06	-0.14	0.0398	Rnf167
ENSMUSG00000076514	1.38	7.62	-2.47	0.0399	Igkv17-121
ENSMUSG00000027603	771.65	367.14	1.07	0.0399	Ggt7
ENSMUSG00000116946	80.43	123.72	-0.62	0.0399	Gm41442
ENSMUSG00000033446	418.93	657.33	-0.65	0.0399	Lpar6
ENSMUSG00000118084	16.58	6.81	1.29	0.0400	AA388235
ENSMUSG00000039231	429.97	345.10	0.32	0.0400	Suv39h1
ENSMUSG00000043488	12.90	26.51	-1.04	0.0400	Frmd8os
ENSMUSG00000032259	61.46	9.45	2.69	0.0401	Drd2
ENSMUSG00000106391	12.56	22.10	-0.80	0.0401	Gm42690
ENSMUSG00000121080	4.19	0.00	4.36	0.0402	
ENSMUSG00000039145	486.18	370.54	0.39	0.0403	Camk1d
ENSMUSG00000051378	62.46	42.15	0.57	0.0403	Kif18b
ENSMUSG00000117592	2.46	7.89	-1.61	0.0405	Gm46603
ENSMUSG00000018965	10964.66	6725.13	0.71	0.0405	Ywhah
ENSMUSG00000066072	1.36	5.70	-2.06	0.0406	Cyp4a10
ENSMUSG00000056486	656.23	338.97	0.95	0.0406	Chn1
ENSMUSG00000028177	1.20	5.34	-2.05	0.0407	1810013D15Rik
ENSMUSG00000100826	268.79	146.05	0.88	0.0407	Snhg14
ENSMUSG00000114051	2.15	6.21	-1.59	0.0408	Gm48008
ENSMUSG00000107750	3.99	9.36	-1.26	0.0408	Gm44013
ENSMUSG00000017715	1558.25	1214.73	0.36	0.0408	Pgs1
ENSMUSG00000056812	122.41	12.27	3.32	0.0408	St8sia3
ENSMUSG00000041272	161.31	77.47	1.06	0.0410	Tox
ENSMUSG00000089818	0.97	3.99	-2.13	0.0410	Gm15950
ENSMUSG00000004892	227.61	63.34	1.84	0.0411	Bcan
ENSMUSG00000045967	213.63	14.14	3.92	0.0413	Gpr158

ENSMUSG00000020889	9013.70	12318.08	-0.45	0.0414	Nr1d1
ENSMUSG00000028453	345.55	289.88	0.25	0.0414	Fancg
ENSMUSG00000096336	4.21	12.35	-1.52	0.0415	Igkv1-135
ENSMUSG00000055452	166.59	128.83	0.37	0.0415	Gm7353
ENSMUSG00000027894	675.89	399.21	0.76	0.0416	Slc6a17
ENSMUSG00000031256	516.69	432.74	0.26	0.0417	Cstf2
ENSMUSG00000032715	218.92	304.99	-0.48	0.0417	Trib3
ENSMUSG00000035455	56.82	40.24	0.50	0.0417	Fignl1
ENSMUSG00000106995	30.97	2.54	3.59	0.0418	Gm33167
ENSMUSG00000007476	1673.82	1395.13	0.26	0.0418	Lrrc8a
ENSMUSG00000042225	220.55	169.83	0.38	0.0418	Ammecr1
ENSMUSG00000039533	338.04	119.32	1.50	0.0419	Mmd2
ENSMUSG00000030657	623.23	506.52	0.30	0.0420	Xylt1
ENSMUSG00000027347	169.10	83.61	1.02	0.0420	Rasgrp1
ENSMUSG00000021048	1246.54	1420.81	-0.19	0.0421	Mthfd1
ENSMUSG00000068196	3103.20	2046.28	0.60	0.0421	Col8a1
ENSMUSG00000032244	1385.77	1061.02	0.38	0.0421	Fem1b
ENSMUSG00000049985	38.63	17.54	1.14	0.0421	Ankrd55
ENSMUSG00000063522	0.53	3.01	-2.73	0.0421	Ly6m
ENSMUSG00000120874	22.77	36.09	-0.66	0.0421	
ENSMUSG00000051537	143.06	58.52	1.29	0.0422	Gm5124
ENSMUSG00000069805	21.68	8.30	1.41	0.0422	Fbp1
ENSMUSG00000109651	0.22	2.29	-3.06	0.0422	Gm45463
ENSMUSG00000041565	29.27	5.74	2.34	0.0423	L3mbtl4
ENSMUSG00000022621	362.07	295.52	0.29	0.0423	Rabl2
ENSMUSG00000022391	2860.43	1907.32	0.58	0.0423	Rangap1
ENSMUSG00000060882	38.89	8.44	2.22	0.0424	Kcnd2
ENSMUSG00000037872	299.44	152.29	0.98	0.0425	Ackr1
ENSMUSG00000048215	1.51	9.10	-2.46	0.0425	A630023P12Rik
ENSMUSG00000001657	68.51	161.67	-1.24	0.0426	Hoxc8
ENSMUSG00000051146	342.69	163.48	1.07	0.0427	Camk2n2
ENSMUSG00000037509	243.88	130.82	0.90	0.0428	Arhgef4
ENSMUSG00000038453	290.42	98.63	1.56	0.0429	Srcin1
ENSMUSG00000120130	16.55	9.44	0.82	0.0430	
ENSMUSG00000041216	39.01	9.20	2.08	0.0430	Clvs1
ENSMUSG00000049971	49.04	10.21	2.26	0.0431	Glt1d1
ENSMUSG00000042942	103.85	60.55	0.77	0.0431	Greb1l
ENSMUSG00000101581	37.00	18.30	1.02	0.0432	C430002N11Rik
ENSMUSG00000032392	421.14	588.99	-0.48	0.0433	Parp16
ENSMUSG00000046032	1681.37	1443.62	0.22	0.0433	Snx12
ENSMUSG00000102269	6.79	14.19	-1.08	0.0434	Gm7357
ENSMUSG00000029403	465.79	256.03	0.86	0.0434	Cdkl2
ENSMUSG00000060371	19.39	5.43	1.83	0.0434	Caln1
ENSMUSG00000086150	50.30	32.45	0.63	0.0434	Bach2os
ENSMUSG00000108325	2.50	0.00	3.61	0.0435	Gm44898
ENSMUSG00000026442	1057.77	585.91	0.85	0.0435	Nfasc
ENSMUSG00000024186	408.57	264.32	0.63	0.0435	Rgs11

ENSMUSG00000017417	365.39	269.86	0.44	0.0435	Plxdc1
ENSMUSG00000090925	6.59	1.29	2.28	0.0437	1810064F22Rik
ENSMUSG00000030079	987.45	857.82	0.20	0.0438	Ruvbl1
ENSMUSG00000047307	53.34	37.43	0.51	0.0439	Pcdhb13
ENSMUSG00000089661	4.55	1.06	2.06	0.0439	Mia
ENSMUSG00000031302	232.62	130.91	0.83	0.0440	Nlgn3
ENSMUSG00000030257	963.95	570.65	0.76	0.0440	Srgap3
ENSMUSG00000117010	9.13	17.76	-0.94	0.0440	Gm49933
ENSMUSG00000087242	24.16	6.95	1.78	0.0440	C78197
ENSMUSG00000091890	0.22	2.93	-3.41	0.0441	A830073O21Rik
ENSMUSG00000085181	17.46	4.16	2.07	0.0442	Gm12709
ENSMUSG00000085657	7.61	18.42	-1.29	0.0443	Gm15984
ENSMUSG00000078521	17.62	8.63	1.04	0.0443	Aunip
ENSMUSG00000039070	3.30	0.00	4.01	0.0443	Cpa4
ENSMUSG00000079259	12.55	1.60	2.95	0.0443	Trim71
ENSMUSG00000049907	1910.25	1372.96	0.48	0.0444	Rasl11b
ENSMUSG00000007216	295.02	347.78	-0.24	0.0445	Zfp775
ENSMUSG00000085971	76.09	107.64	-0.50	0.0445	Gm15411
ENSMUSG00000028633	1945.37	1495.36	0.38	0.0447	Ctps
ENSMUSG00000109957	28.88	11.26	1.36	0.0447	Gm45353
ENSMUSG00000038668	1148.79	868.96	0.40	0.0448	Lpar1
ENSMUSG00000073234	9.66	0.82	3.55	0.0448	Gm8773
ENSMUSG00000085953	8.57	0.00	5.39	0.0449	Gm15496
ENSMUSG00000024462	2498.67	1554.44	0.68	0.0449	Gabbr1
ENSMUSG00000046834	9.46	3.84	1.31	0.0449	Krt1
ENSMUSG00000051228	17.94	28.49	-0.69	0.0450	Nyx
ENSMUSG00000004031	151.61	34.16	2.15	0.0450	Brinp2
ENSMUSG00000033900	196.64	96.38	1.03	0.0450	Map9
ENSMUSG00000030228	1.08	4.93	-2.15	0.0452	Pik3c2g
ENSMUSG00000097974	93.21	50.08	0.89	0.0452	Gm10605
ENSMUSG00000061601	187.35	73.22	1.36	0.0452	Pclo
ENSMUSG00000069919	12729.88	30082.40	-1.24	0.0452	Hba-a1
ENSMUSG00000021986	82.72	7.86	3.39	0.0453	Amer2
ENSMUSG00000097845	1.43	5.88	-1.98	0.0453	A230020J21Rik
ENSMUSG00000053141	87.36	20.14	2.12	0.0453	Ptprt
ENSMUSG00000023277	1082.31	1348.76	-0.32	0.0453	Twf2
ENSMUSG00000037610	1.97	0.00	3.27	0.0453	Kcnmb2
ENSMUSG00000051435	55.21	32.51	0.76	0.0453	Fhad1
ENSMUSG00000082100	5.77	12.97	-1.16	0.0454	Glns-ps1
ENSMUSG00000051650	127.52	85.51	0.57	0.0454	B3gnt2
ENSMUSG00000121141	1877.60	1423.75	0.40	0.0454	
ENSMUSG00000097290	229.36	279.78	-0.28	0.0455	1300002E11Rik
ENSMUSG00000027570	349.57	134.60	1.37	0.0455	Col9a3
ENSMUSG00000029154	9.57	2.59	1.91	0.0455	Cwh43
ENSMUSG00000026220	8.70	3.14	1.50	0.0456	Slc16a14
ENSMUSG00000030516	2503.02	2859.45	-0.19	0.0456	Tjp1
ENSMUSG00000005447	213.93	133.28	0.68	0.0456	Pafah1b3

ENSMUSG00000006850	467.20	535.75	-0.20	0.0456	Tmco6
ENSMUSG00000027490	365.32	293.82	0.31	0.0457	E2f1
ENSMUSG00000105419	58.43	92.55	-0.66	0.0458	Gm43205
ENSMUSG00000047414	890.43	713.48	0.32	0.0458	Flrt2
ENSMUSG00000034848	435.97	337.96	0.37	0.0458	Ttc21b
ENSMUSG00000051238	432.26	538.70	-0.32	0.0458	Swsap1
ENSMUSG00000117286	23.88	3.98	2.59	0.0459	Gm1043
ENSMUSG00000030510	2.55	8.18	-1.70	0.0460	Cers3
ENSMUSG00000015619	244.38	128.59	0.93	0.0461	Gata3
ENSMUSG00000120849	121.25	89.71	0.43	0.0462	
ENSMUSG00000040464	388.94	483.56	-0.31	0.0463	Gtpbp10
ENSMUSG00000038605	392.98	226.91	0.79	0.0463	Samd10
ENSMUSG00000105128	0.38	3.17	-2.85	0.0463	Gm42870
ENSMUSG00000031139	16.18	1.86	3.11	0.0463	Mcf2
ENSMUSG00000042743	301.14	152.74	0.98	0.0464	Sgtb
ENSMUSG00000046287	24.89	3.10	3.01	0.0464	Pnma3
ENSMUSG00000064342	4.33	12.84	-1.55	0.0464	mt-Ti
ENSMUSG00000049092	100.16	49.35	1.02	0.0464	Gpr137c
ENSMUSG00000111669	7.51	0.00	5.20	0.0465	Gm31698
ENSMUSG00000020635	286.93	170.84	0.75	0.0465	Fkbp1b
ENSMUSG00000045348	955.82	528.33	0.85	0.0465	Nyap1
ENSMUSG00000020847	220.16	132.01	0.74	0.0466	Rph3al
ENSMUSG00000114399	3.28	7.63	-1.23	0.0468	Gm47994
ENSMUSG00000087484	159.12	202.30	-0.34	0.0469	2900089D17Rik
ENSMUSG00000079055	296.95	184.50	0.68	0.0469	Slc8a3
ENSMUSG00000100691	306.71	397.04	-0.37	0.0470	2010320M18Rik
ENSMUSG00000121075	27.23	39.67	-0.53	0.0470	
ENSMUSG00000034382	81.30	56.88	0.51	0.0471	AI661453
ENSMUSG00000090141	0.71	4.57	-2.73	0.0471	Gm614
ENSMUSG00000023000	368.54	268.33	0.46	0.0471	Dhh
ENSMUSG00000032332	1125.68	929.85	0.28	0.0471	Col12a1
ENSMUSG00000026426	4885.39	3511.67	0.48	0.0472	Arl8a
ENSMUSG00000036298	437.98	296.21	0.56	0.0472	Slc2a13
ENSMUSG00000041439	1829.57	1294.32	0.50	0.0472	Mfsd6
ENSMUSG00000064371	0.44	3.27	-2.88	0.0472	mt-Tt
ENSMUSG00000051502	361.99	187.72	0.95	0.0473	Ufsp1
ENSMUSG00002074868	2.51	0.33	2.91	0.0474	
ENSMUSG00000117239	36.52	17.27	1.06	0.0474	Gpr31c
ENSMUSG00000052305	18278.05	41438.32	-1.18	0.0474	Hbb-bs
ENSMUSG00000030844	778.73	627.45	0.31	0.0474	Rgs10
ENSMUSG00000031644	806.98	424.49	0.93	0.0475	Nek1
ENSMUSG00000112941	28.95	16.41	0.81	0.0475	Gm48623
ENSMUSG00000000869	174.31	106.24	0.71	0.0476	Il4
ENSMUSG00000039841	200.35	165.18	0.28	0.0476	Zfp800
ENSMUSG00000043126	4.09	12.62	-1.58	0.0477	D830039M14Rik
ENSMUSG00000030307	9.50	0.55	4.10	0.0477	Slc6a11
ENSMUSG00000085017	0.26	2.75	-3.31	0.0477	Gm13412

ENSMUSG00000109669	54.15	35.93	0.59	0.0477	Gm45472
ENSMUSG00000078143	8.92	18.11	-1.05	0.0478	Gm17344
ENSMUSG00000030607	2624.08	1816.95	0.53	0.0478	Acan
ENSMUSG00000019923	5367.73	2718.79	0.98	0.0479	Zwint
ENSMUSG00000095197	0.00	6.79	-5.36	0.0479	Ighv1-59
ENSMUSG00000028410	5408.04	4334.08	0.32	0.0479	Dnaja1
ENSMUSG00000096950	13.65	6.98	0.99	0.0480	Gm9530
ENSMUSG00000035835	321.47	211.89	0.60	0.0480	Plppr3
ENSMUSG00000026494	980.55	629.39	0.64	0.0480	Kif26b
ENSMUSG00000087652	3.79	0.24	3.48	0.0480	Gm15918
ENSMUSG00000056941	1306.30	1173.06	0.15	0.0481	Commd7
ENSMUSG00000021589	563.22	474.45	0.25	0.0481	Rhobtb3
ENSMUSG00000021687	1867.05	1302.60	0.52	0.0482	Scamp1
ENSMUSG00000027887	90.32	193.72	-1.10	0.0482	Sypl2
ENSMUSG00000099309	0.26	2.96	-3.43	0.0482	Mir7085
ENSMUSG00000112711	0.00	2.59	-3.97	0.0482	Gm32552
ENSMUSG00000104235	0.77	3.81	-2.45	0.0483	Gm37589
ENSMUSG00000097006	140.28	208.36	-0.57	0.0485	9530082P21Rik
ENSMUSG00000029769	533.06	303.54	0.81	0.0486	Ccdc136
ENSMUSG00000027568	54.27	0.49	6.66	0.0486	Ntsr1
ENSMUSG00000030226	9.11	2.27	1.95	0.0486	Lmo3
ENSMUSG00000020669	118.96	81.04	0.56	0.0487	Sh3yl1
ENSMUSG00000120465	13.90	24.12	-0.78	0.0487	
ENSMUSG00000114069	2.42	0.24	2.87	0.0488	E130119H09Rik
ENSMUSG00000039081	1250.97	1601.12	-0.36	0.0488	Zfp503
ENSMUSG00000106871	1.80	6.36	-1.83	0.0488	Gm3289
ENSMUSG00000039934	297.53	219.44	0.44	0.0489	Gsap
ENSMUSG00000116380	5.65	11.86	-1.05	0.0489	Gm39556
ENSMUSG00000030699	88.64	111.63	-0.34	0.0490	Tbx6
ENSMUSG00000056367	327.85	161.09	1.03	0.0490	Actr3b
ENSMUSG00000045327	11.86	23.01	-0.93	0.0490	6330549D23Rik
ENSMUSG00000097150	8.44	16.46	-0.94	0.0491	Gm26513
ENSMUSG00000056755	119.59	7.28	4.04	0.0491	Grm7
ENSMUSG00000085852	18.88	9.22	1.01	0.0492	Gm13807
ENSMUSG00000047935	5.73	0.55	3.36	0.0492	Sox1ot
ENSMUSG00000005357	41.47	21.75	0.93	0.0493	Slc1a6
ENSMUSG00000026311	774.34	556.07	0.48	0.0493	Asb1
ENSMUSG00000062554	664.81	834.60	-0.33	0.0494	Gm12751
ENSMUSG00000044906	457.71	383.60	0.26	0.0494	4930503L19Rik
ENSMUSG00000024593	136.02	72.37	0.91	0.0494	Megf10
ENSMUSG00000028678	63.69	43.29	0.54	0.0494	Kif2c
ENSMUSG00000040740	716.47	1309.78	-0.87	0.0495	Slc25a34
ENSMUSG00000092167	2.45	0.33	2.85	0.0495	Gm3696
ENSMUSG00000120039	26.21	6.54	1.99	0.0495	
ENSMUSG00000045404	200.20	155.62	0.36	0.0495	Kcnk13
ENSMUSG00000089809	433.49	315.26	0.46	0.0496	Rasgef1b
ENSMUSG00000032719	83.95	62.17	0.42	0.0496	Sbspon

ENSMUSG00000048304	11.72	1.34	3.11	0.0496	Slitrk3
ENSMUSG00000061527	9.58	1.45	2.73	0.0497	Krt5
ENSMUSG00000108722	0.44	2.82	-2.68	0.0498	Gm34225
ENSMUSG00000046982	1211.49	1380.43	-0.19	0.0500	Tshz1
